# Supplementary material for: Pediatric Myalgic Encephalomyelitis/Chronic Fatigue Syndrome (ME/CFS): A Diagnostic and Communication Case Study for Health Care Providers in Training
Source: MedEdPORTAL. 2025 Mar 14;21:11507. doi: 10.15766/mep_2374-8265.11507 (PMC11906784; doi:10.15766/mep_2374-8265.11507)
Supplement: Supplementary file 1 — MECFS Presentation.pptxMECFS Part 1.mp4MECFS Part 2.mp4Survey Questions.docx [file mep_2374-8265.11507-s001.zip › D. Survey Questions.docx]

**Survey Questions for ME/CFS Pediatric Case Study**

Facilitator: The survey questions are for the pre- and post-tests. The pre-test should be given before viewing the slides and videos (preferred to see videos first but order is at the discretion of the facilitator). The pre-test measures the baseline before viewing of the videos and slides, and therefore there are no incorrect answers. The post-test measures changes from baseline in conceptual areas of attitudes and beliefs, and knowledge and application. While the “correct” responses for the post-test will vary, the answers are given for each statement or question. Some of concepts intended to be captured in the question and are 5-point Likert scale for level of familiarity or exposure rather than correct or incorrect. The facilitator can then look for improvement between pre- and post-test.

**Answers**

**PRE-TEST**

Part 1: Demographic and participant data is optional.

Part 2: Statements and questions assess attitudes and beliefs. No correct answers for open text and answers will vary. Correct answer given where applicable.

Part 3: Statements and questions assess knowledge and application. No correct answers for open text and scale questions. Answers will vary. Correct answer given where applicable.

**POST-TEST**

Part 1: Demographic and participant data is optional.

Part 2: Statements and questions assess attitudes and beliefs. While answers for open text will vary, a version of acceptable correct content is outlined. Correct answer given where applicable. After completing the slide show and videos, facilitator should look for an increase in the number of correct answers as compared to the individual’s pre-test.

Part 3: Statements and questions assess knowledge and application. While answers for open text will vary, a version of acceptable correct content is outlined. Correct answer given where applicable. After completing the slide show and videos, facilitator should look for an increase in the number of correct answers as compared to the individual’s pre-test.

**PRE-TEST SURVEY (To be administered/taken ONCE prior to viewing ME/CFS Pediatric Case Study Videos Part 1 and Part 2)**

***PART 1:***

**Demographics and participant data**

1. Age range
   - 1. 20-29
     2. 30--39
     3. 40-49
     4. 50-59
     5. 60 and up
2. Sex
   - 1. Male
     2. Female
     3. Other

3. Year in MD program

1. 1^st^ year
2. 2^nd^ year
3. 3^rd^ year
4. 4^th^ year

***PART 2:***

**Attitudes and Beliefs**

4***.*** I think ‘PEM (post-exertional malaise)’ means… (open text)

5. When a patient complains of fatigue, I think….(open text)

6. Myalgic Encephalomyelitis/Chronic Fatigue Syndrome (ME/CFS) is… (choose one) **ANSWER**: Medical condition

- - 1. A medical condition
    2. A psychiatric condition
    3. both medical and psychiatric
    4. not sure

7. When communicating with a pediatric patient with ME/CFS it is important to….(open text)

8. When managing a pediatric patient with ME/CFS it is important to … (open text)

***PART 3:***

***Knowledge and Application***

9. When in your curriculum do you learn about ME/CFS? (drop down menu – check all that apply)

- 1. 1^st^ year
  2. 2^nd^ year
  3. 3^rd^ year
  4. 4^th^ year
  5. never

10. Please indicate your level of personal familiarity with ME/CFS (scale 0 = not at all familiar; 5 = highly familiar)

0 1 2 3 4 5

11. Please indicate your level of clinical exposure to ME/CFS (scale 0 = never exposed; 5 = highly exposed)

- - - - 1. 1 2 3 4 5

12. Who is affected by ME/CFS? (open text)

1. When should ME/CFS be considered in a child or adolescent? (open text)
2. Three important things I know about pediatric ME/CFS are…(open text)
3. ME/CFS affects children and adolescents in the following ways… (check all that apply). **ANSWER**: all the following are correct.
   1. Decreases physical and cognitive abilities
   2. Disrupts social activities and school
   3. Affects patient’s ability to maintain friendships and activities outside the house
   4. Can make children and adolescents housebound
   5. Reduces educational achievement, employment status and income as an adult
4. What 4 steps are required to diagnose ME/CFS? (open text)
5. Which of the following principles are important for diagnosing and managing ME/CFS? (check all that apply). **ANSWER**: all the following are correct.
   1. Rule out other conditions with similar symptoms and known causes.
   2. Treat underlying/comorbid conditions.
   3. Use laboratory diagnostic efforts to identify underlying conditions and pathophysiologic mechanisms.
   4. Treatment and management aims for symptom alleviation or elimination.
   5. Non-pharmacologic therapy and rehabilitation may assist in symptom relief.
6. Who should be part of a multidisciplinary care team for pediatric patients with ME/CFS? (check all that apply). **ANSWER**: all the following are correct.
   1. Pediatrician and other health professionals
   2. School nurse
   3. Education professionals
   4. Families and caregivers
   5. Other (please clarify) – open text
7. On a scale of 0-5, I would rate my ability to diagnose a pediatric patient with ME/CFS as…… **ANSWER**: No correct answer as it is self-rated.

0 1 2 3 4 5

1. On a scale of 0-5 I would rate my confidence to communicate with a pediatric patient with ME/CFS as…. **ANSWER**: No correct answer as it is self-rated.

0 1 2 3 4 5

**Survey Questions for ME/CFS Pediatric Case Study**

**POST-TEST SURVEY (To be administered /taken ONCE after viewing ME/CFS Pediatric Case Study Videos Part 1 and Part 2, and the slides)**

***PART 1: Demographics and participant data (please check)***

1. Age range

1. 20-29
2. 30--39
3. 40-49
4. 50-59
5. 60 and up

2. Sex

- - 1. Male
    2. Female
    3. Other

3. Year in MD program

- - 1. 1^st^ year
    2. 2^nd^ year
    3. 3^rd^ year
    4. 4^th^ year
    5. Other (please indicate)

***PART 2:***

***Attitudes and Beliefs***

4. After viewing/reading the modules, I think ‘PEM (post-exertional malaise)’ means… (open text)

- Malaise includes feeling bad (sick) as well as fatigued
- Patients describe this as “crash” or “relapse” of illness, because all symptoms are worsened, not just fatigue
- Exertion could be physical or mental
- The malaise persists for more than 24 hours
- Leads to additional limitation in activities

5. When a patient complains of fatigue, I think….(open text)

A substantial reduction or impairment in ability to engage in pre-illness levels of activity (occupational, educational, social, or personal life) lasting for more than 6 months that is accompanied by profound, new onset fatigue that is not result of ongoing or unusual excessive exertion, and not substantially alleviated by rest.

6. Myalgic Encephalomyelitis/Chronic Fatigue Syndrome (ME/CFS) is… (choose one)

**ANSWER**: Medical condition.

- 1. A medical condition
  2. A psychiatric condition
  3. both medical and psychiatric
  4. not sure

7. When communicating with a pediatric patient with ME/CFS it is important to….(open text)

Children and adolescents should be encouraged to describe their illness; parents or caregivers may add their observations in the process.

8. When managing a pediatric patient with ME/CFS it is important to … (open text)

- Consider other conditions with similar symptoms and known causes
- Treat underlying/comorbid conditions
- Laboratory diagnostic efforts aim to identify underlying conditions and pathophysiologic mechanisms
- Treatment and management aim to improve symptoms
- Non-pharmacologic therapy and rehabilitation may assist in relief of symptoms

***PART 3:***

***Knowledge and Application***

9. Who is affected by ME/CFS? (open text)

Adults and children are both affected by ME/CFS. ME/CFS is more common in adolescents than children.

10. When should ME/CFS be considered in a child or adolescent? (open text)

Consider ME/CFS if patient or family notes the following symptoms: increased fatigue, cognitive problems, unrefreshing sleep, muscle and/or joint aches, headaches, sore throat, lymph node tenderness

- - Note: fatigue may not always be the primary symptom; children may often report dizziness and/or abdominal aches
- Symptoms increase following activity (post-exertional malaise)
- Symptoms have been present for at least 3 months
- Consequences include reduction in level of previous activities (social, educational, play/sports)
- Onset is new, and other diagnoses are not evident

11. Three important things I know about pediatric ME/CFS are…(open text)

- Using the 2015 IOM ME/CFS Case Definition to make a diagnosis;
- Fatigue is not always the primary symptom; children often report dizziness and/or abdominal aches;
- Consequences include reduction in level of previous activities (social, educational, play/sports).

12. ME/CFS affects children and adolescents in the following ways… (check all that apply). **ANSWER**: all the following are correct.

- 1. Decreases physical and cognitive abilities
  2. Disrupts social activities and school
  3. Affects patient’s ability to maintain friendships and activities outside the house
  4. Can make children and adolescents housebound
  5. Reduces educational achievement, employment status and income as an adult.

13. What 4 steps are required to diagnose ME/CFS? (open text)

1. Use the 2015 IOM ME/CFS case definition diagnostic criteria with core symptoms to guide assessment.

2. Obtain a detailed medical and psychiatric history.

3. Make a thorough physical and mental status examination.

4. Order laboratory screening tests to help identify or rule out other possible conditions that could be treated.

14. Which of the following principles are important for diagnosing and managing ME/CFS? (check all that apply). **ANSWER**: all the following are correct.

- 1. Rule out other conditions with similar symptoms and known causes.
  2. Treat underlying/comorbid conditions.
  3. Use laboratory diagnostic efforts to identify underlying conditions and pathophysiologic mechanisms.
  4. Treatment and management aim for symptom alleviation or elimination.
  5. Non-pharmacologic therapy and rehabilitation may assist in symptom relief.

15. Who should be part of a multidisciplinary care team for pediatric patients with ME/CFS? (check all that apply). **ANSWER**: all the following are correct.

- 1. Pediatrician and other health professionals
  2. School nurse
  3. Education professionals
  4. Families and caregivers
  5. Other (please clarify) – open text

17. On a scale of 0-5, I would rate my ability to diagnose a pediatric patient with ME/CFS as…… **ANSWER**: No correct answer as it is self-rated.

0 1 2 3 4 5

18. On a scale of 0-5 I would rate my confidence to communicate with a pediatric patient with ME/CFS as…. **ANSWER**: No correct answer as it is self-rated.

0 1 2 3 4 5

19. Please list three reasons why you feel more confident in managing a pediatric patient with ME/CFS. (open text)
