# Supplementary material for: Pediatric Myalgic Encephalomyelitis/Chronic Fatigue Syndrome (ME/CFS): A Diagnostic and Communication Case Study for Health Care Providers in Training
Source: MedEdPORTAL. 2025 Mar 14;21:11507. doi: 10.15766/mep_2374-8265.11507 (PMC11906784; doi:10.15766/mep_2374-8265.11507)
Supplement: Supplementary file 1 — MECFS Presentation.pptxMECFS Part 1.mp4MECFS Part 2.mp4Survey Questions.docx [file mep_2374-8265.11507-s001.zip › A. MECFS Presentation.pptx]

## Slide 1
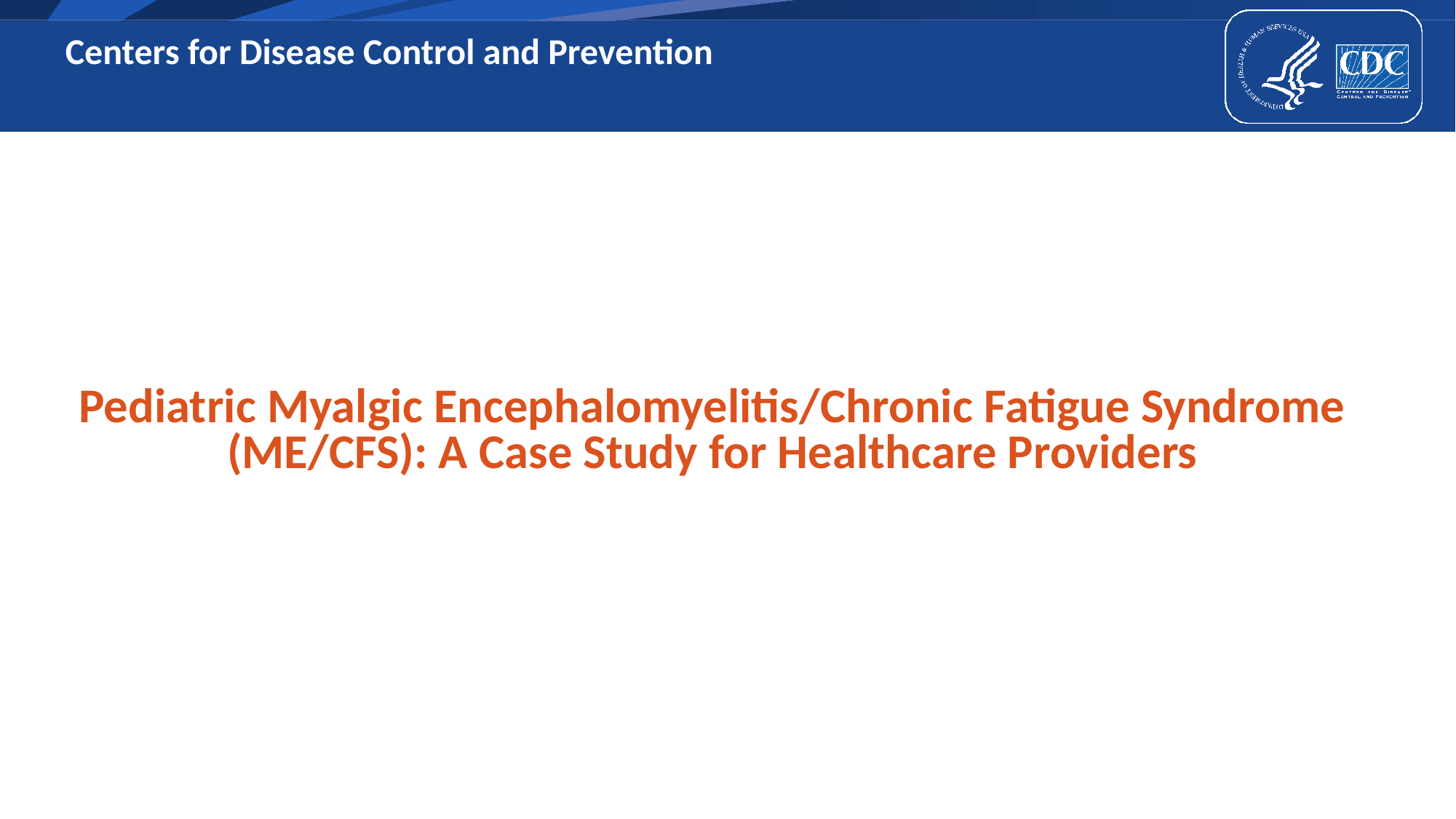

# Pediatric Myalgic Encephalomyelitis/Chronic Fatigue Syndrome (ME/CFS): A Case Study for Healthcare Providers

## Slide 2
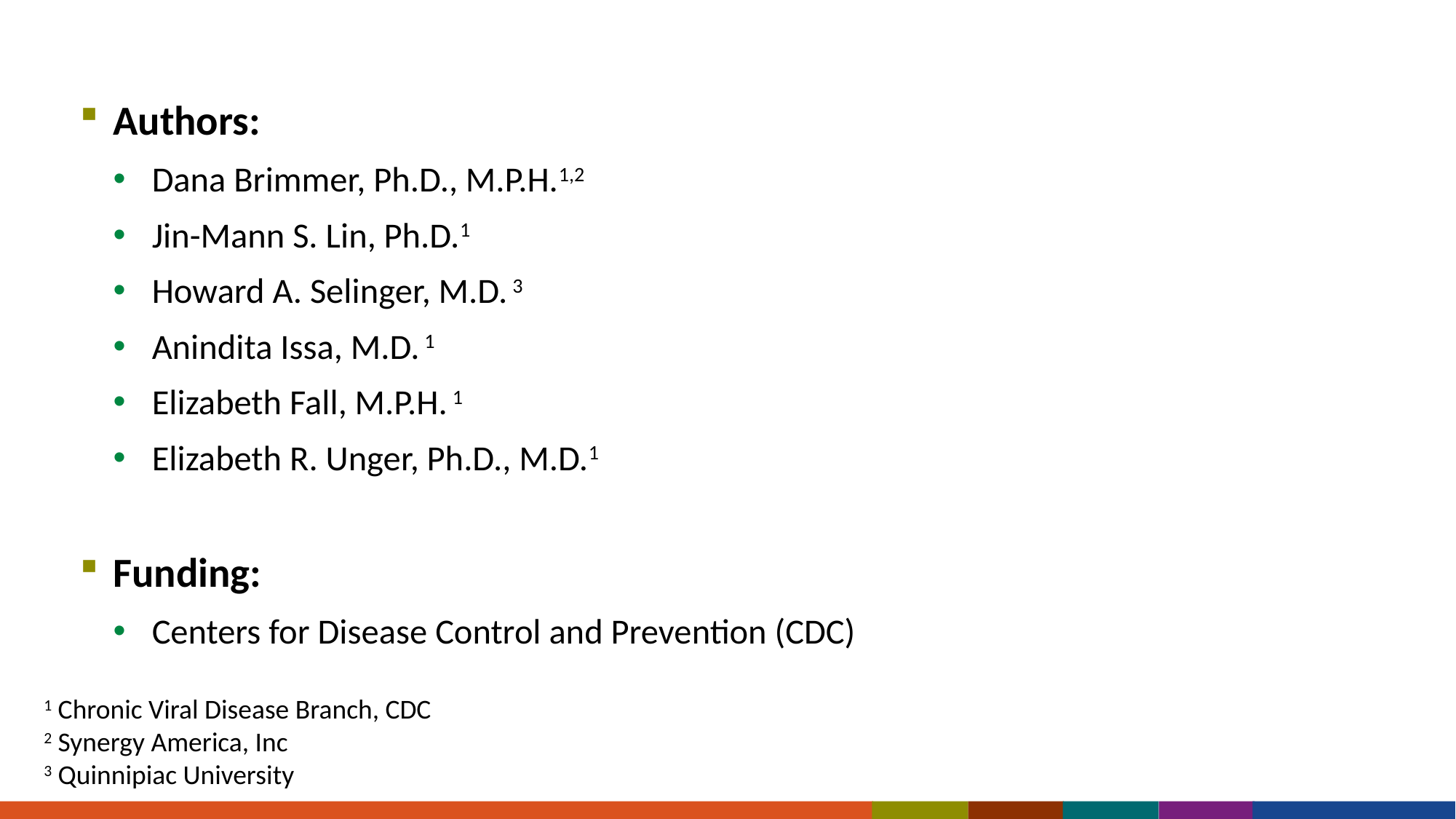

Authors:
Dana Brimmer, Ph.D., M.P.H.1,2
Jin-Mann S. Lin, Ph.D.1
Howard A. Selinger, M.D. 3
Anindita Issa, M.D. 1
Elizabeth Fall, M.P.H. 1
Elizabeth R. Unger, Ph.D., M.D.1
Funding:
Centers for Disease Control and Prevention (CDC)
1 Chronic Viral Disease Branch, CDC
2 Synergy America, Inc
3 Quinnipiac University

## Slide 3
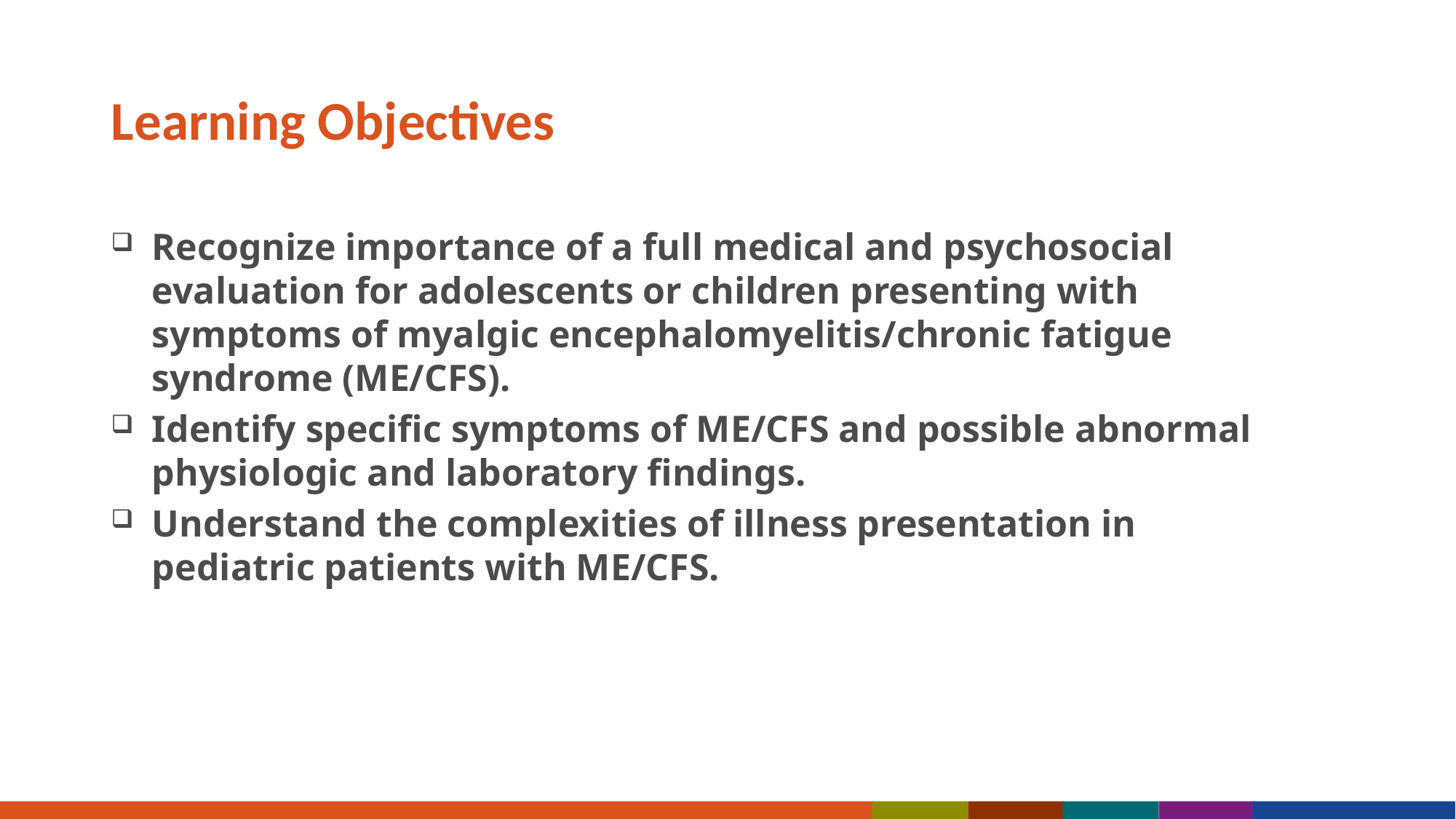

# Learning Objectives
Recognize importance of a full medical and psychosocial evaluation for adolescents or children presenting with symptoms of myalgic encephalomyelitis/chronic fatigue syndrome (ME/CFS).
Identify specific symptoms of ME/CFS and possible abnormal physiologic and laboratory findings.
Understand the complexities of illness presentation in pediatric patients with ME/CFS.

## Slide 4
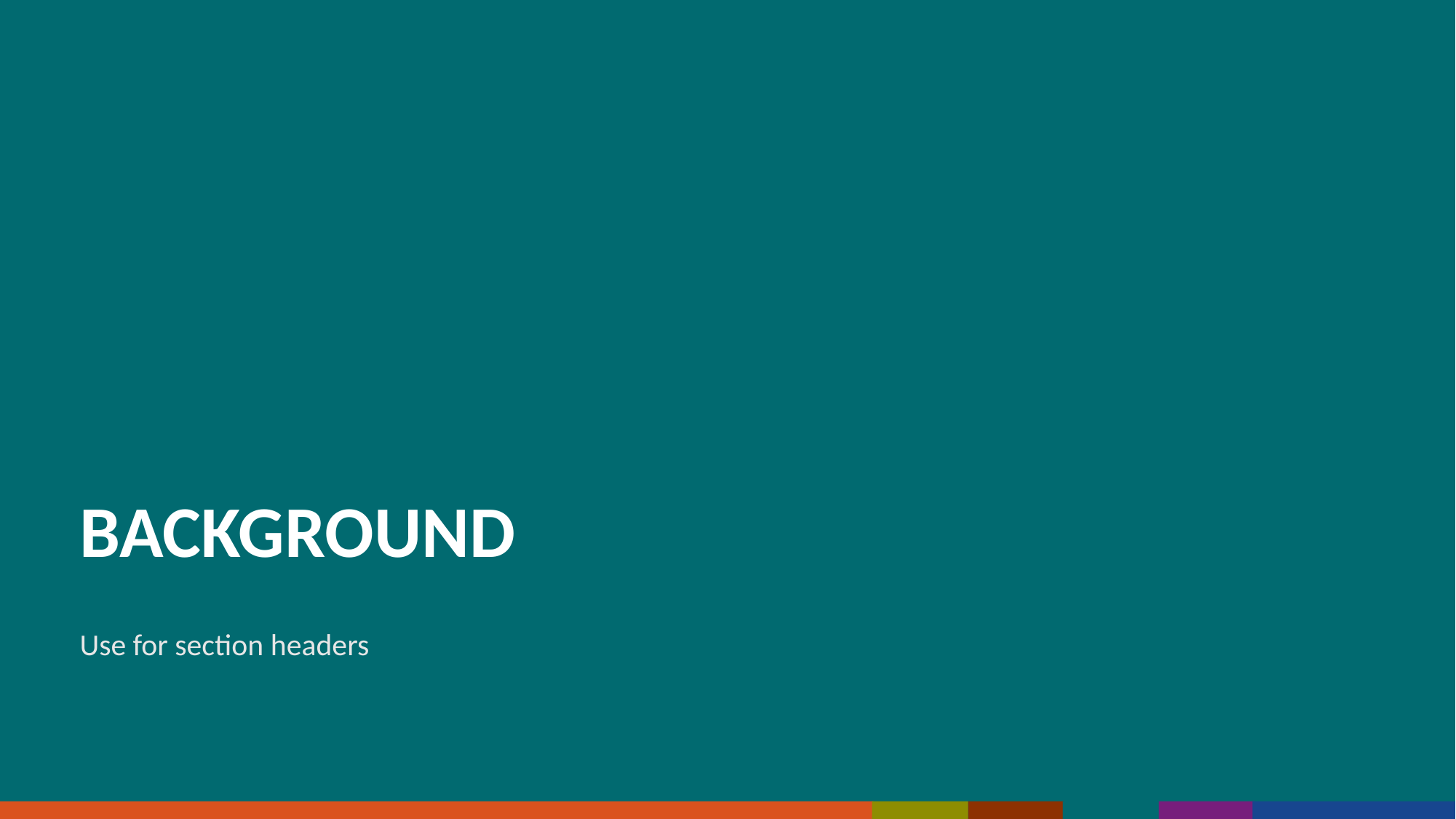

BACKGROUND
Use for section headers

## Slide 5
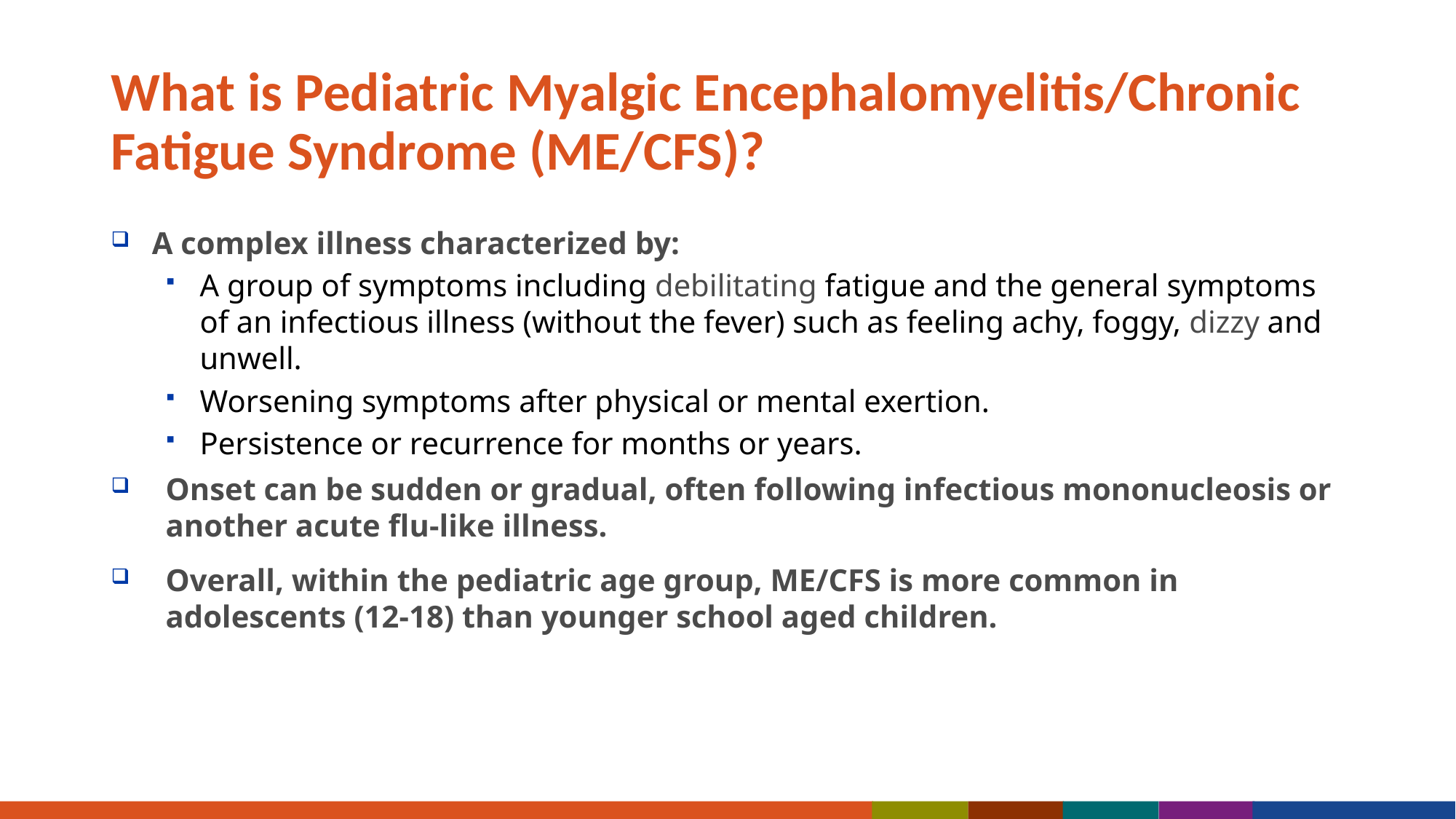

# What is Pediatric Myalgic Encephalomyelitis/Chronic Fatigue Syndrome (ME/CFS)?
A complex illness characterized by:
A group of symptoms including debilitating fatigue and the general symptoms of an infectious illness (without the fever) such as feeling achy, foggy, dizzy and unwell.
Worsening symptoms after physical or mental exertion.
Persistence or recurrence for months or years.
Onset can be sudden or gradual, often following infectious mononucleosis or another acute flu-like illness.
Overall, within the pediatric age group, ME/CFS is more common in adolescents (12-18) than younger school aged children.

## Slide 6
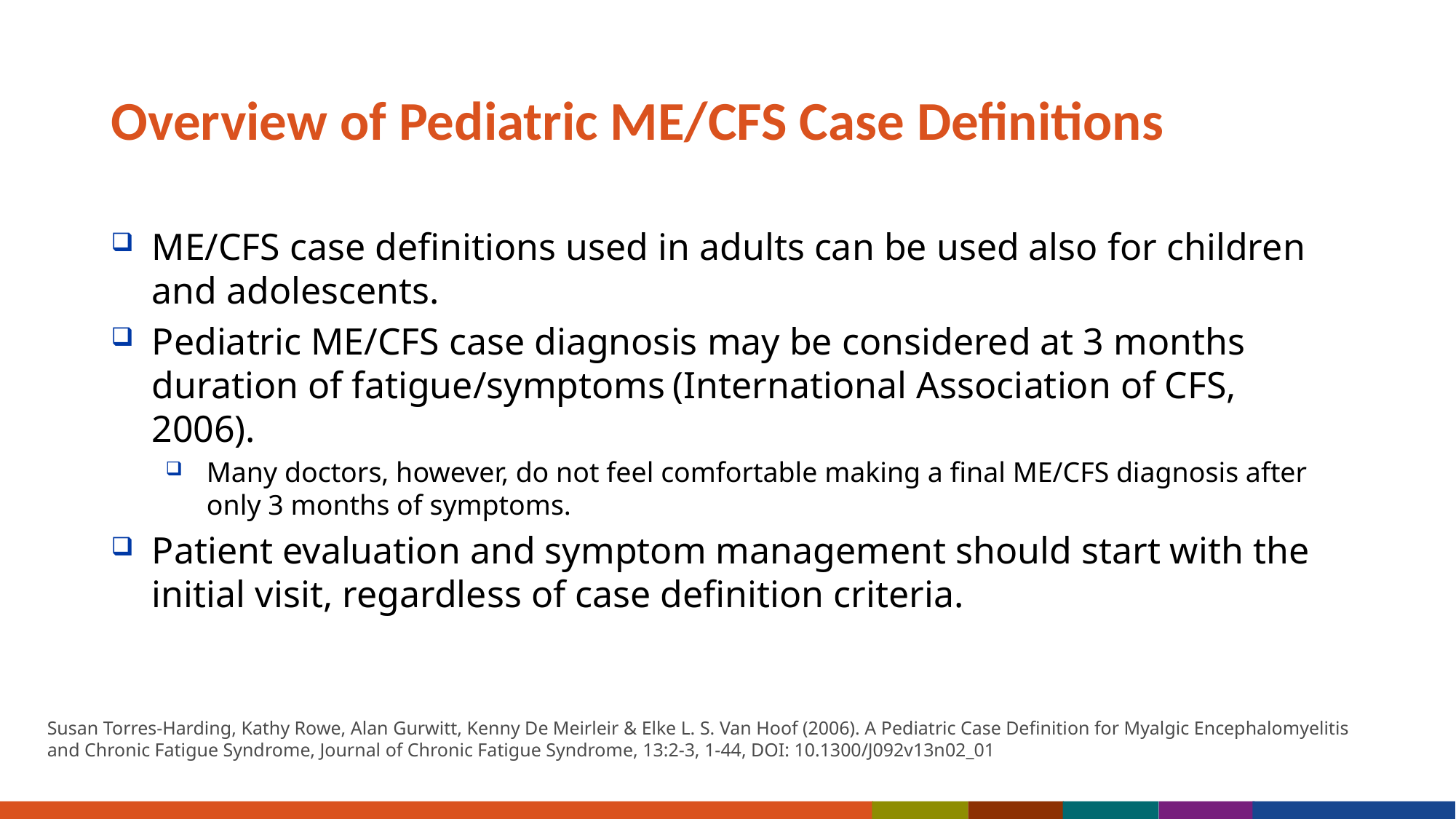

# Overview of Pediatric ME/CFS Case Definitions
ME/CFS case definitions used in adults can be used also for children and adolescents.
Pediatric ME/CFS case diagnosis may be considered at 3 months duration of fatigue/symptoms (International Association of CFS, 2006).
Many doctors, however, do not feel comfortable making a final ME/CFS diagnosis after only 3 months of symptoms.
Patient evaluation and symptom management should start with the initial visit, regardless of case definition criteria.
Susan Torres-Harding, Kathy Rowe, Alan Gurwitt, Kenny De Meirleir & Elke L. S. Van Hoof (2006). A Pediatric Case Definition for Myalgic Encephalomyelitis and Chronic Fatigue Syndrome, Journal of Chronic Fatigue Syndrome, 13:2-3, 1-44, DOI: 10.1300/J092v13n02_01

## Slide 7
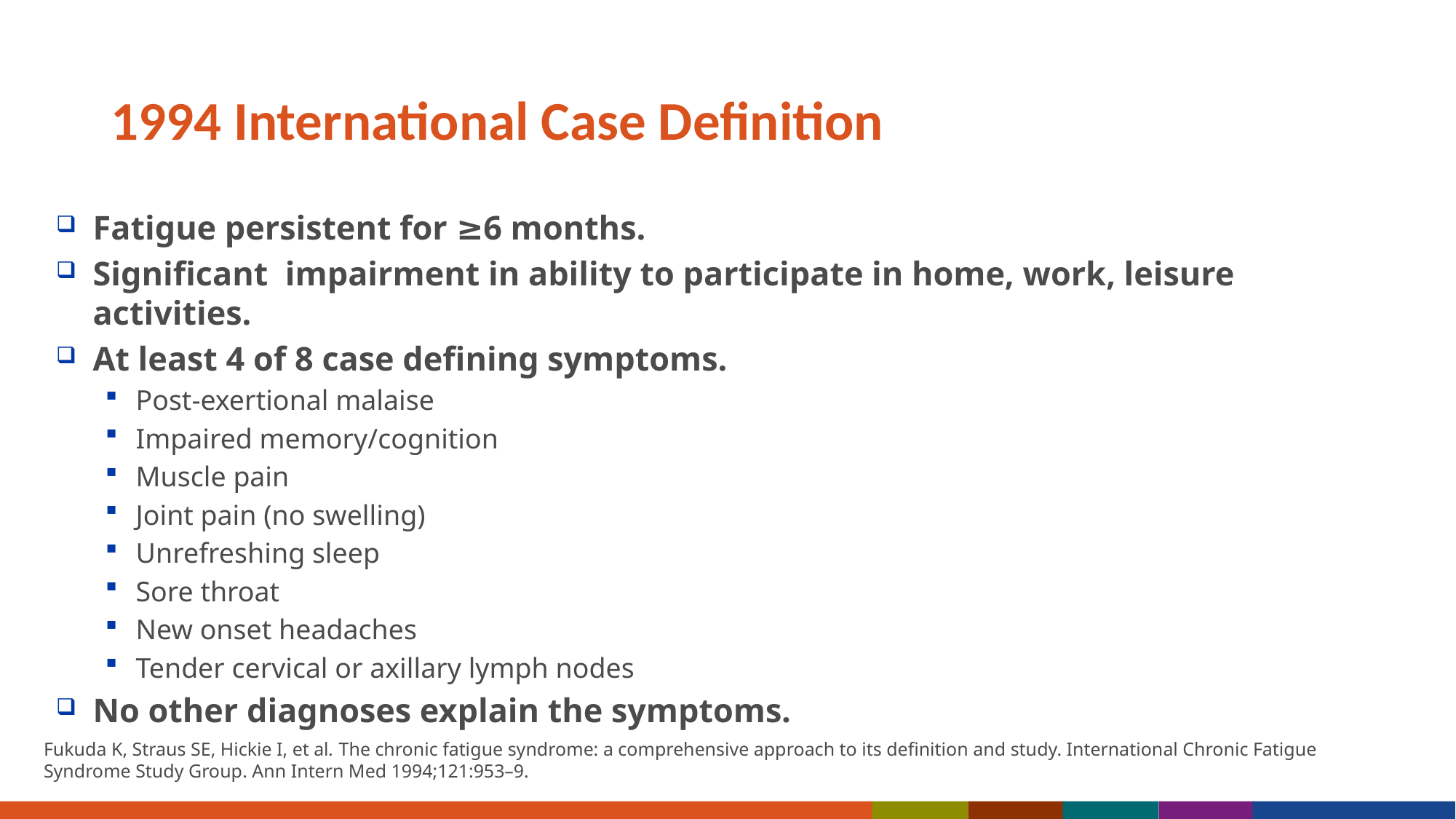

# 1994 International Case Definition
Fatigue persistent for ≥6 months.
Significant impairment in ability to participate in home, work, leisure activities.
At least 4 of 8 case defining symptoms.
Post-exertional malaise
Impaired memory/cognition
Muscle pain
Joint pain (no swelling)
Unrefreshing sleep
Sore throat
New onset headaches
Tender cervical or axillary lymph nodes
No other diagnoses explain the symptoms.
Fukuda K, Straus SE, Hickie I, et al. The chronic fatigue syndrome: a comprehensive approach to its definition and study. International Chronic Fatigue Syndrome Study Group. Ann Intern Med 1994;121:953–9.

## Slide 8
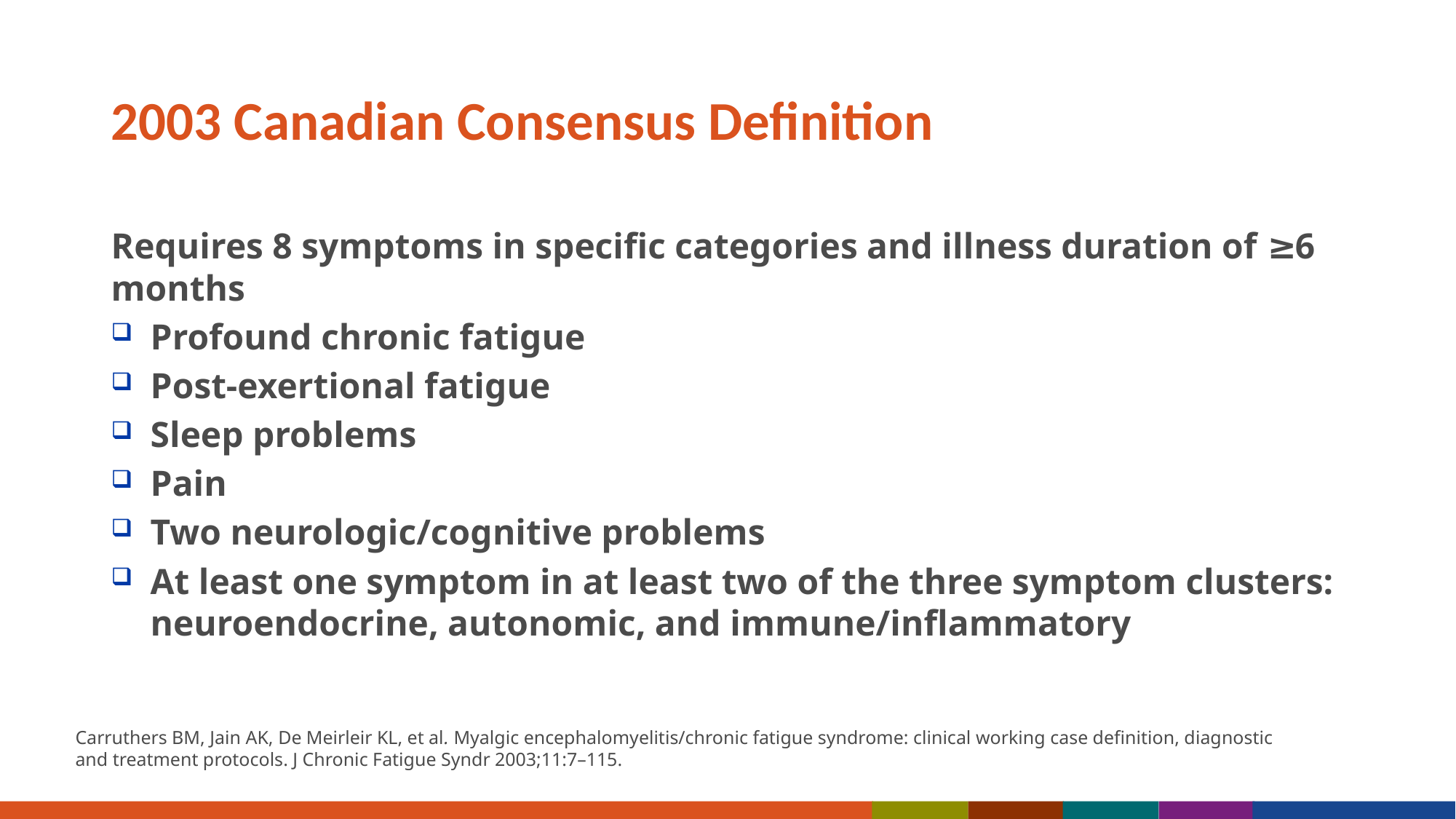

# 2003 Canadian Consensus Definition
Requires 8 symptoms in specific categories and illness duration of ≥6 months
Profound chronic fatigue
Post-exertional fatigue
Sleep problems
Pain
Two neurologic/cognitive problems
At least one symptom in at least two of the three symptom clusters: neuroendocrine, autonomic, and immune/inflammatory
Carruthers BM, Jain AK, De Meirleir KL, et al. Myalgic encephalomyelitis/chronic fatigue syndrome: clinical working case definition, diagnostic and treatment protocols. J Chronic Fatigue Syndr 2003;11:7–115.

## Slide 9
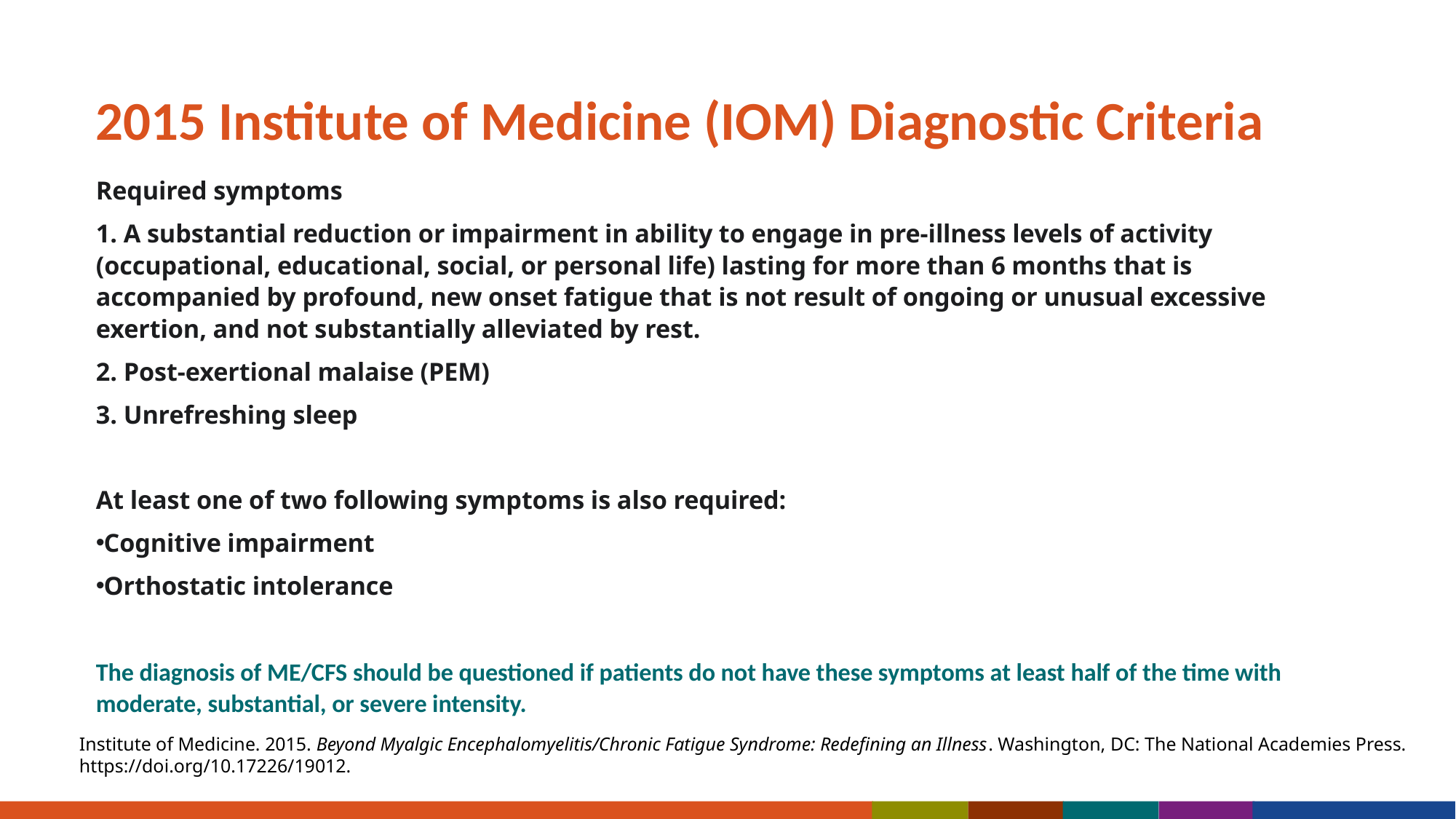

# 2015 Institute of Medicine (IOM) Diagnostic Criteria
Required symptoms
1. A substantial reduction or impairment in ability to engage in pre-illness levels of activity (occupational, educational, social, or personal life) lasting for more than 6 months that is accompanied by profound, new onset fatigue that is not result of ongoing or unusual excessive exertion, and not substantially alleviated by rest.
2. Post-exertional malaise (PEM)
3. Unrefreshing sleep
At least one of two following symptoms is also required:
Cognitive impairment
Orthostatic intolerance
The diagnosis of ME/CFS should be questioned if patients do not have these symptoms at least half of the time with moderate, substantial, or severe intensity.
Institute of Medicine. 2015. Beyond Myalgic Encephalomyelitis/Chronic Fatigue Syndrome: Redefining an Illness. Washington, DC: The National Academies Press. https://doi.org/10.17226/19012.

## Slide 10
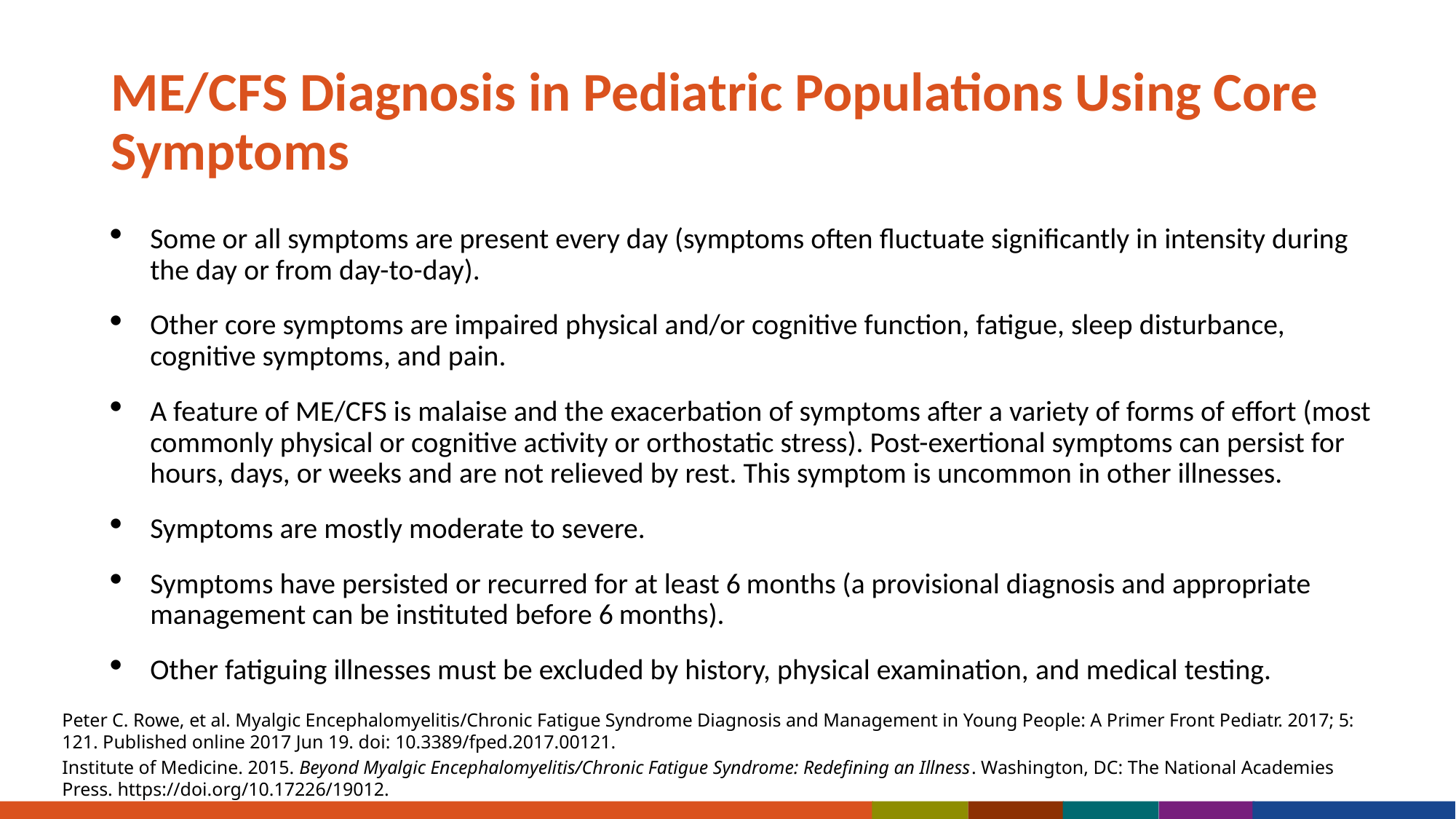

# ME/CFS Diagnosis in Pediatric Populations Using Core Symptoms
Some or all symptoms are present every day (symptoms often fluctuate significantly in intensity during the day or from day-to-day).
Other core symptoms are impaired physical and/or cognitive function, fatigue, sleep disturbance, cognitive symptoms, and pain.
A feature of ME/CFS is malaise and the exacerbation of symptoms after a variety of forms of effort (most commonly physical or cognitive activity or orthostatic stress). Post-exertional symptoms can persist for hours, days, or weeks and are not relieved by rest. This symptom is uncommon in other illnesses.
Symptoms are mostly moderate to severe.
Symptoms have persisted or recurred for at least 6 months (a provisional diagnosis and appropriate management can be instituted before 6 months).
Other fatiguing illnesses must be excluded by history, physical examination, and medical testing.
Peter C. Rowe, et al. Myalgic Encephalomyelitis/Chronic Fatigue Syndrome Diagnosis and Management in Young People: A Primer Front Pediatr. 2017; 5: 121. Published online 2017 Jun 19. doi: 10.3389/fped.2017.00121.
Institute of Medicine. 2015. Beyond Myalgic Encephalomyelitis/Chronic Fatigue Syndrome: Redefining an Illness. Washington, DC: The National Academies Press. https://doi.org/10.17226/19012.

## Slide 11
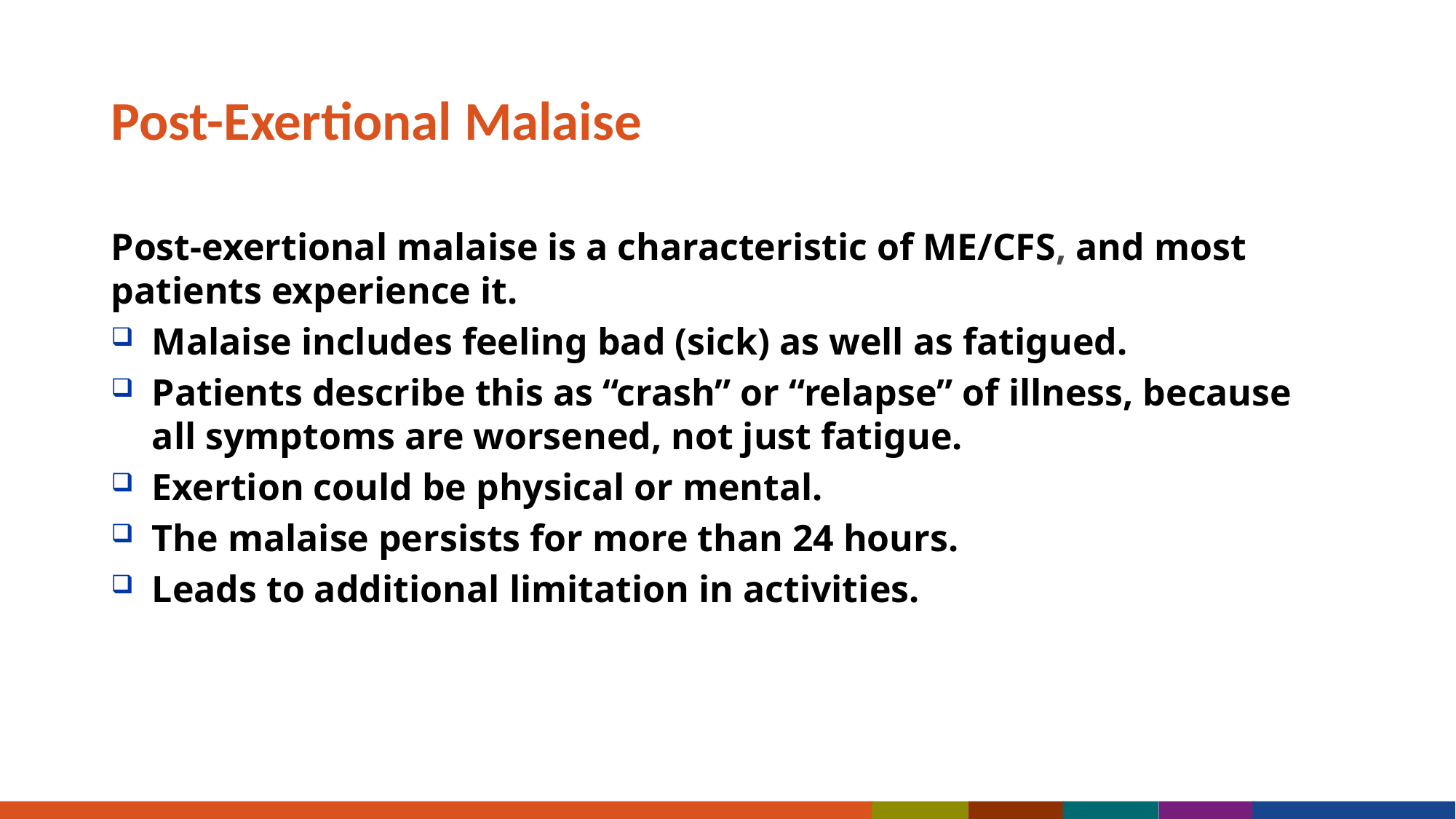

# Post-Exertional Malaise
Post-exertional malaise is a characteristic of ME/CFS, and most patients experience it.
Malaise includes feeling bad (sick) as well as fatigued.
Patients describe this as “crash” or “relapse” of illness, because all symptoms are worsened, not just fatigue.
Exertion could be physical or mental.
The malaise persists for more than 24 hours.
Leads to additional limitation in activities.

## Slide 12
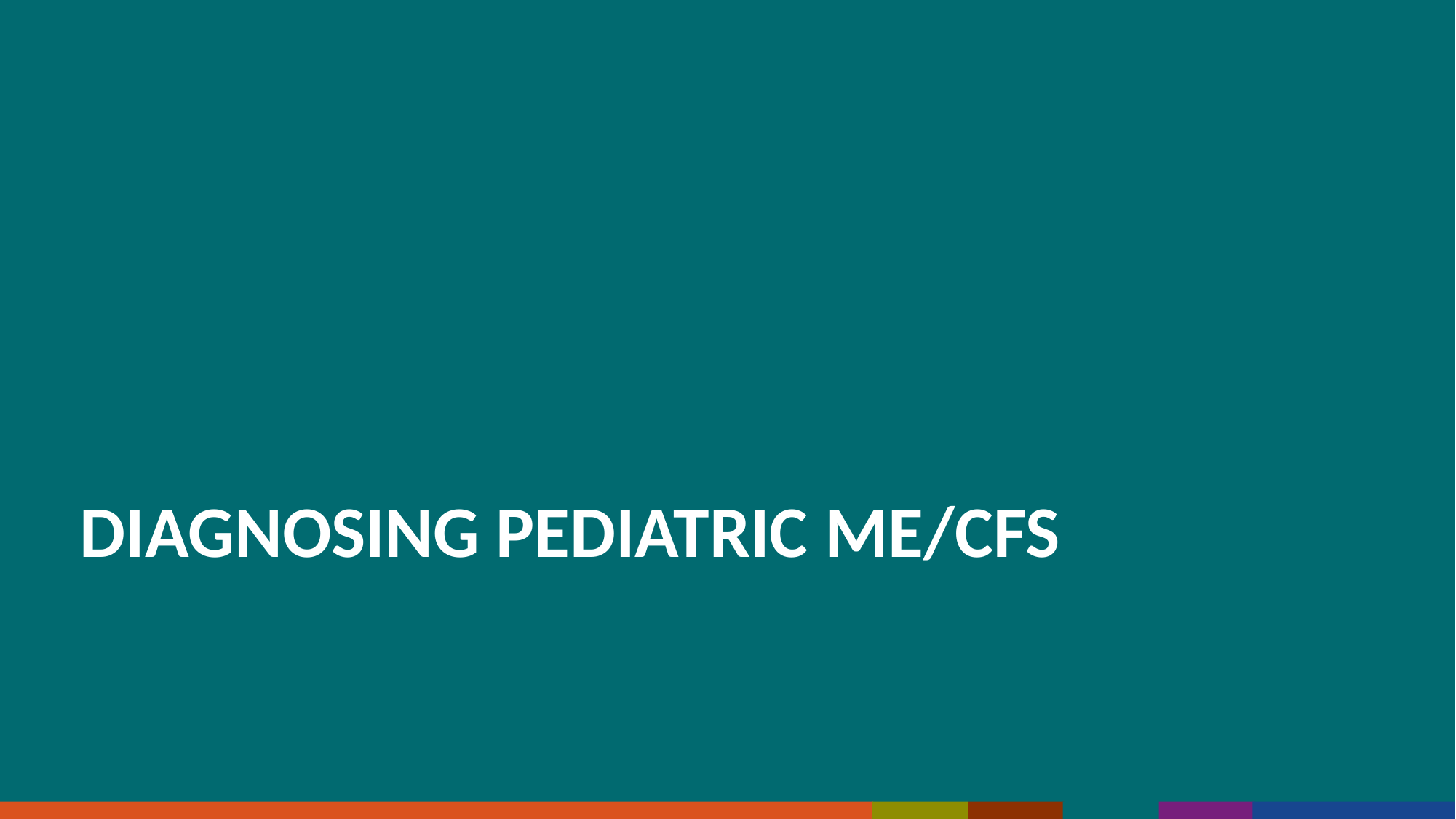

Diagnosing pediatric ME/cfs

## Slide 13
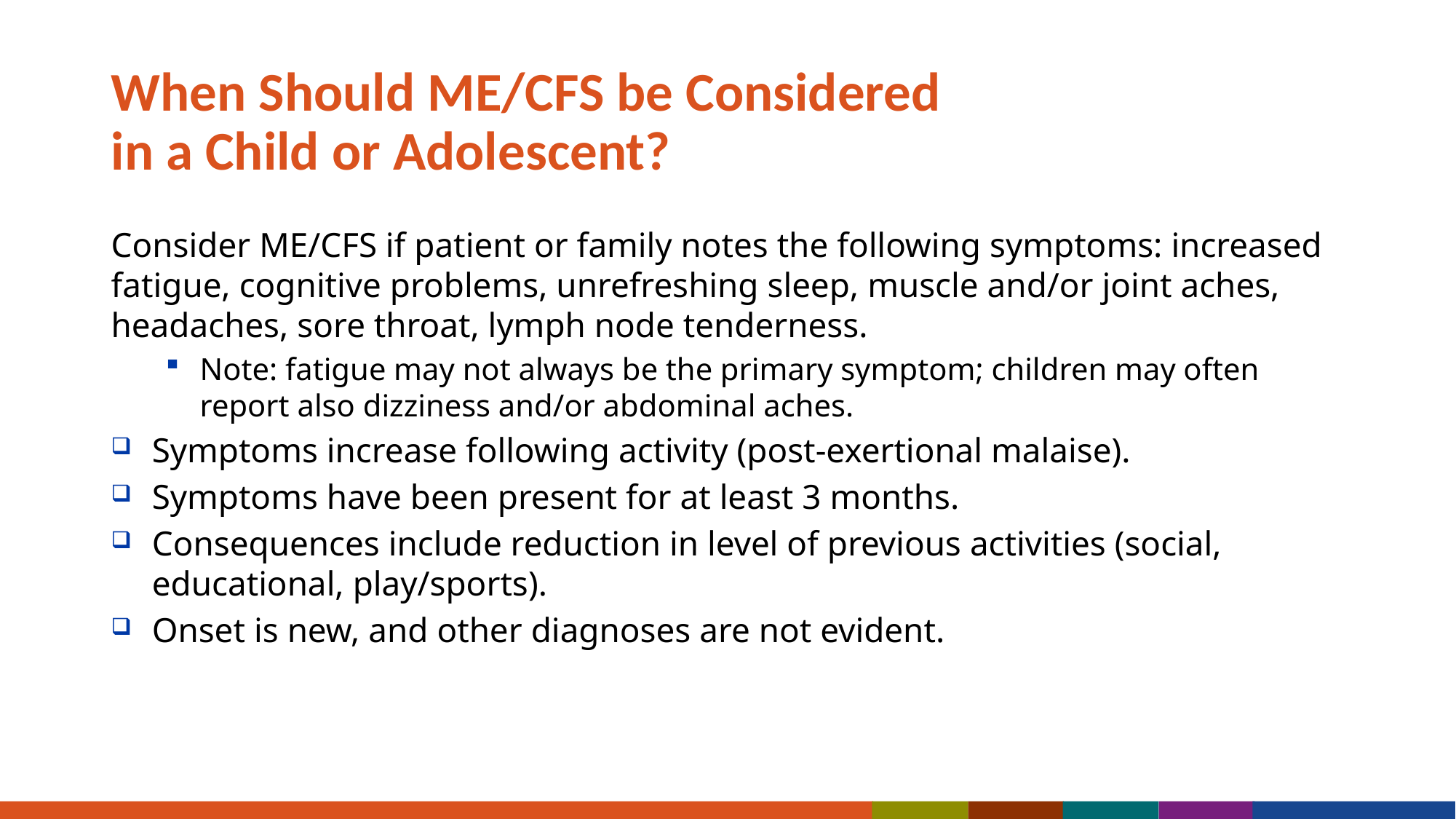

# When Should ME/CFS be Considered in a Child or Adolescent?
Consider ME/CFS if patient or family notes the following symptoms: increased fatigue, cognitive problems, unrefreshing sleep, muscle and/or joint aches, headaches, sore throat, lymph node tenderness.
Note: fatigue may not always be the primary symptom; children may often report also dizziness and/or abdominal aches.
Symptoms increase following activity (post-exertional malaise).
Symptoms have been present for at least 3 months.
Consequences include reduction in level of previous activities (social, educational, play/sports).
Onset is new, and other diagnoses are not evident.

## Slide 14
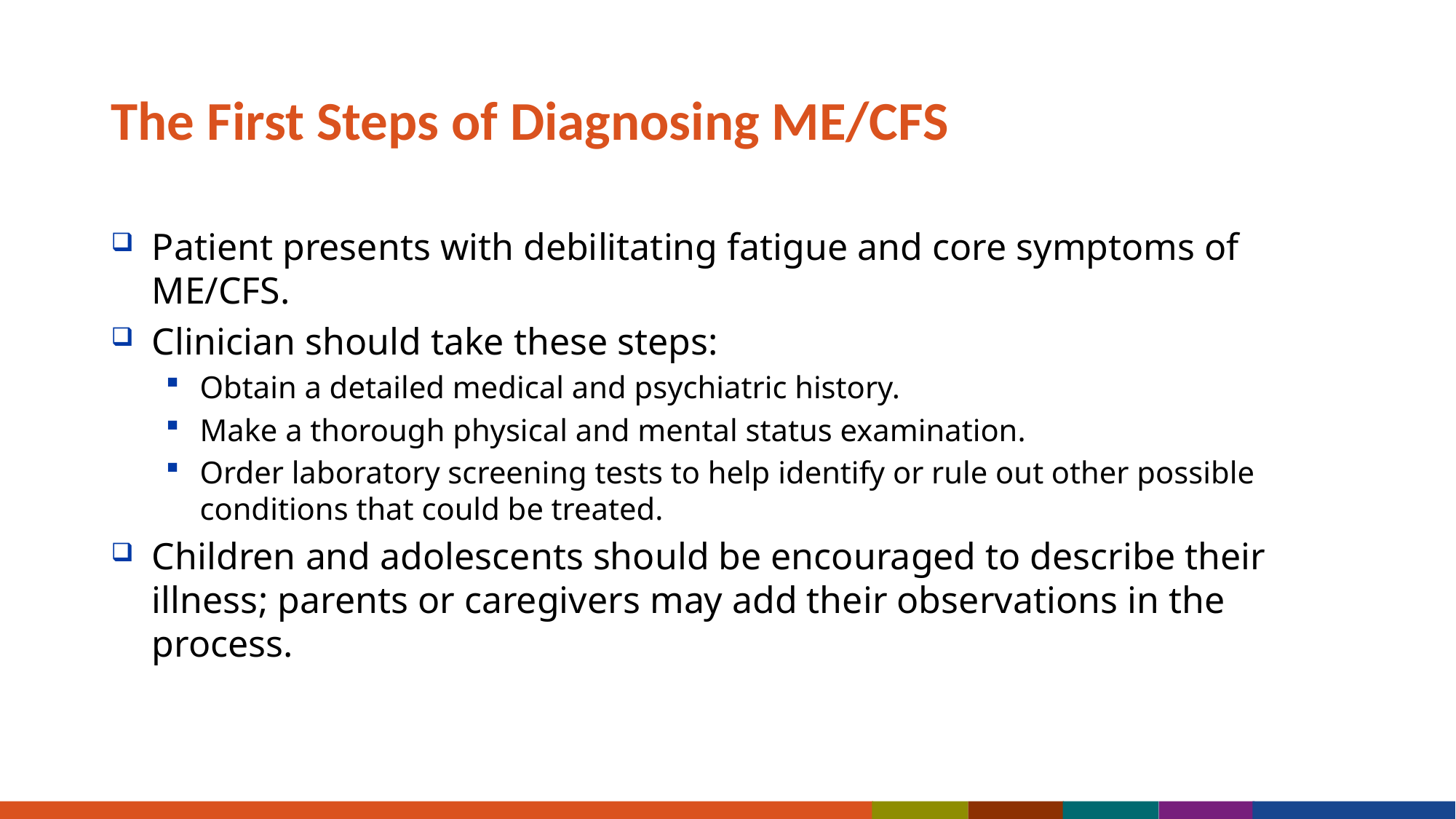

# The First Steps of Diagnosing ME/CFS
Patient presents with debilitating fatigue and core symptoms of ME/CFS.
Clinician should take these steps:
Obtain a detailed medical and psychiatric history.
Make a thorough physical and mental status examination.
Order laboratory screening tests to help identify or rule out other possible conditions that could be treated.
Children and adolescents should be encouraged to describe their illness; parents or caregivers may add their observations in the process.

## Slide 15
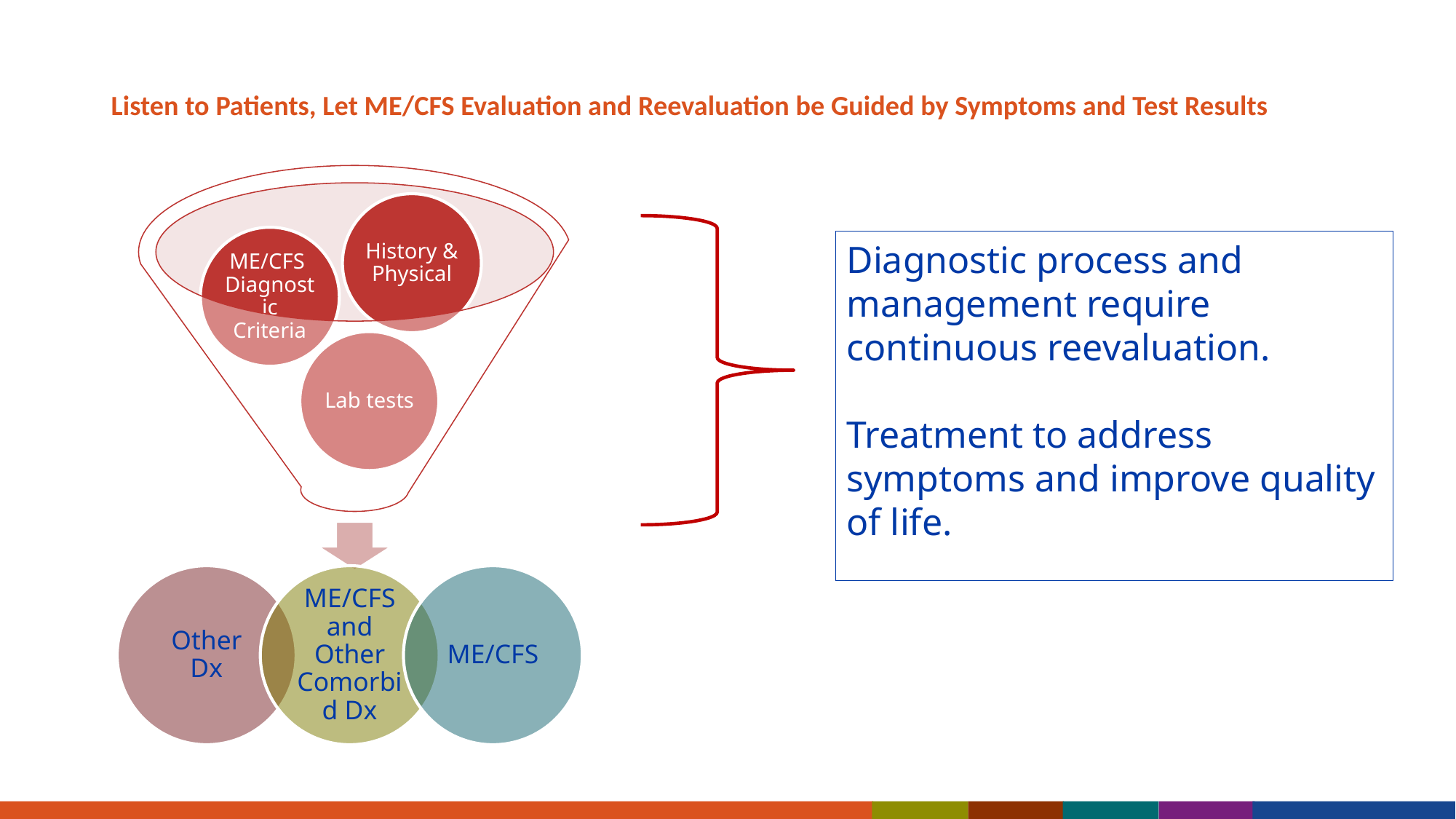

# Listen to Patients, Let ME/CFS Evaluation and Reevaluation be Guided by Symptoms and Test Results
Diagnostic process and management require continuous reevaluation.
Treatment to address symptoms and improve quality of life.

## Slide 16
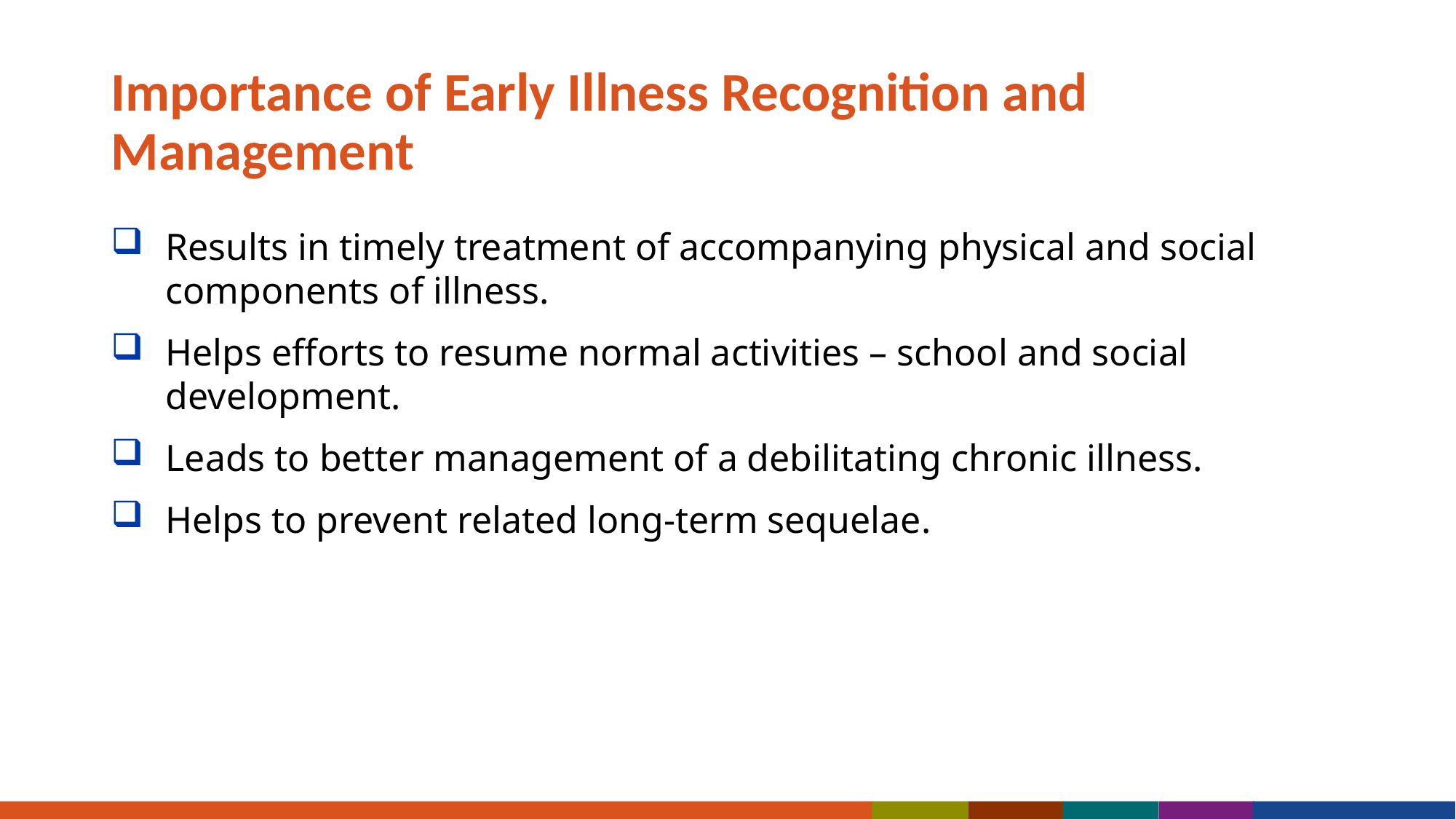

# Importance of Early Illness Recognition and Management
Results in timely treatment of accompanying physical and social components of illness.
Helps efforts to resume normal activities – school and social development.
Leads to better management of a debilitating chronic illness.
Helps to prevent related long-term sequelae.

## Slide 17
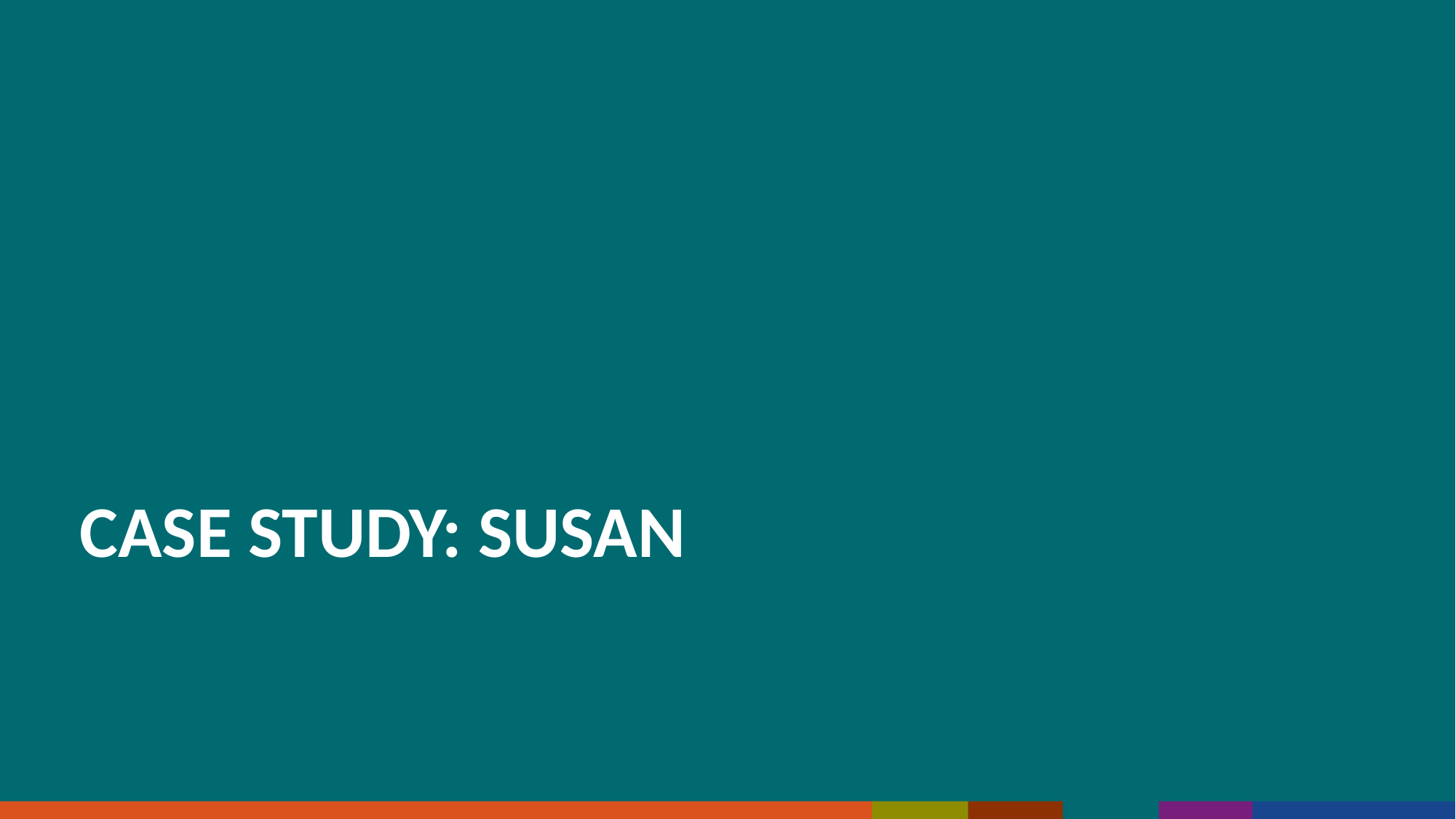

Case Study: susan

## Slide 18
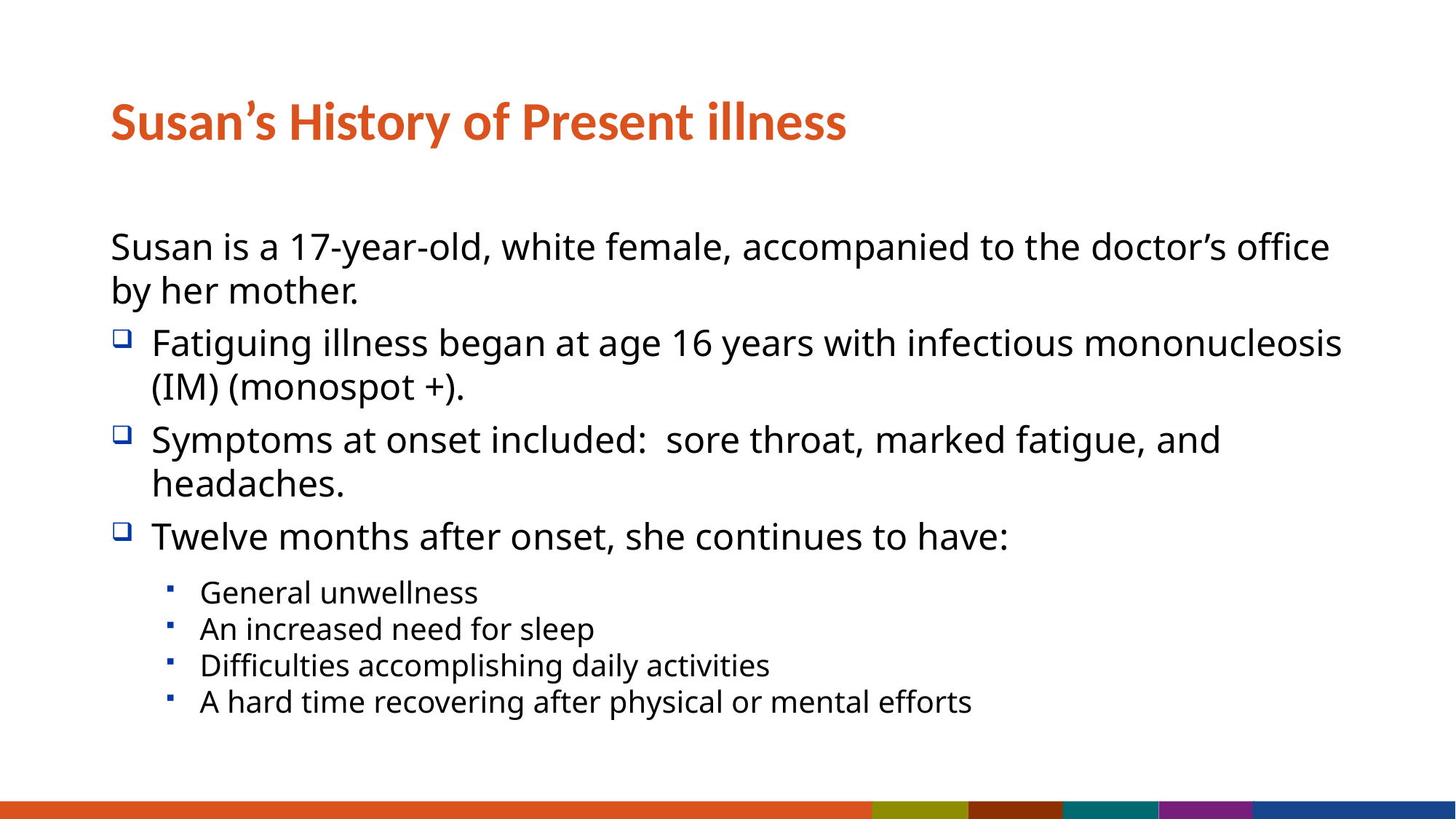

# Susan’s History of Present illness
Susan is a 17-year-old, white female, accompanied to the doctor’s office by her mother.
Fatiguing illness began at age 16 years with infectious mononucleosis (IM) (monospot +).
Symptoms at onset included: sore throat, marked fatigue, and headaches.
Twelve months after onset, she continues to have:
General unwellness
An increased need for sleep
Difficulties accomplishing daily activities
A hard time recovering after physical or mental efforts

## Slide 19
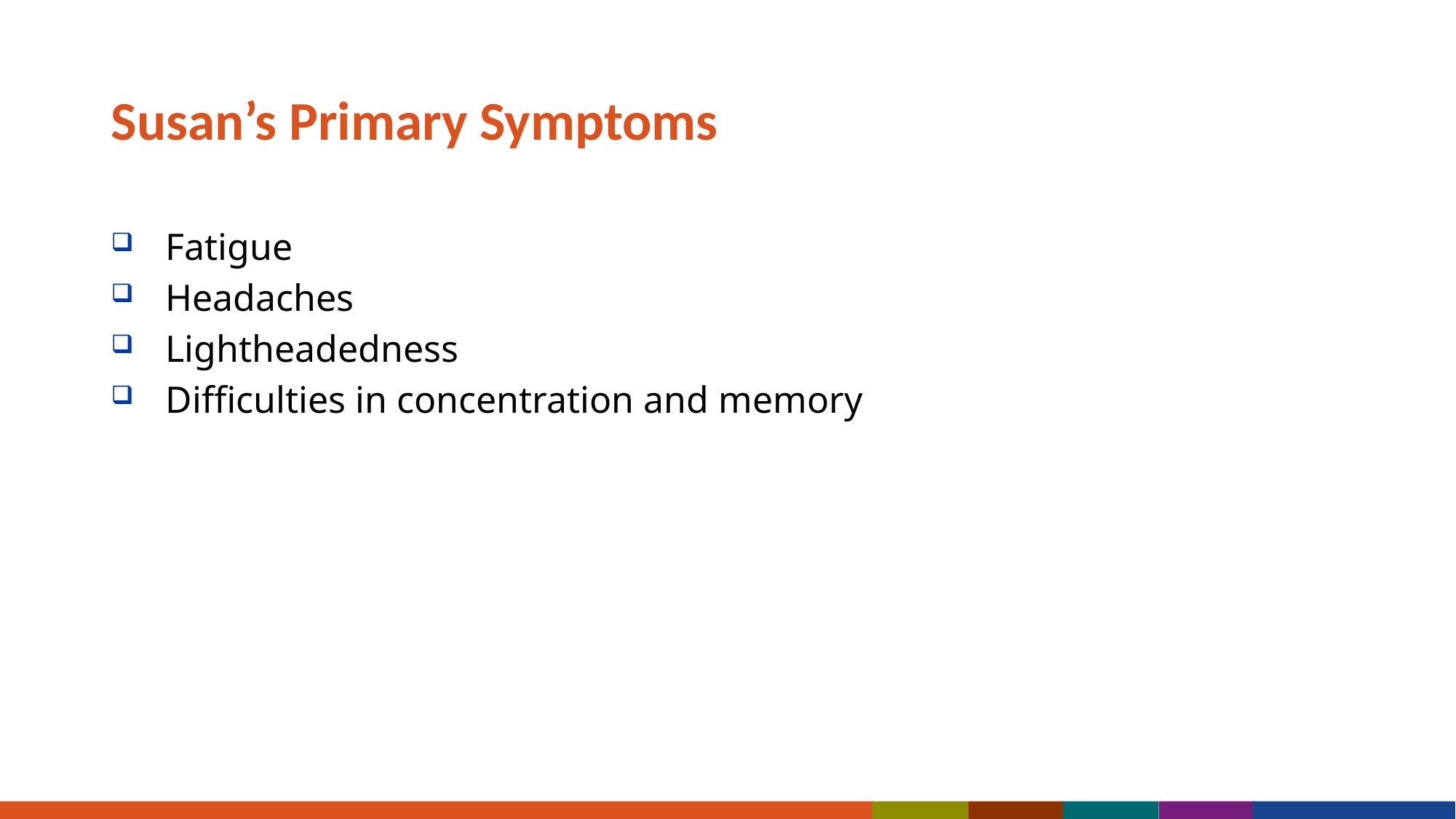

# Susan’s Primary Symptoms
Fatigue
Headaches
Lightheadedness
Difficulties in concentration and memory

## Slide 20
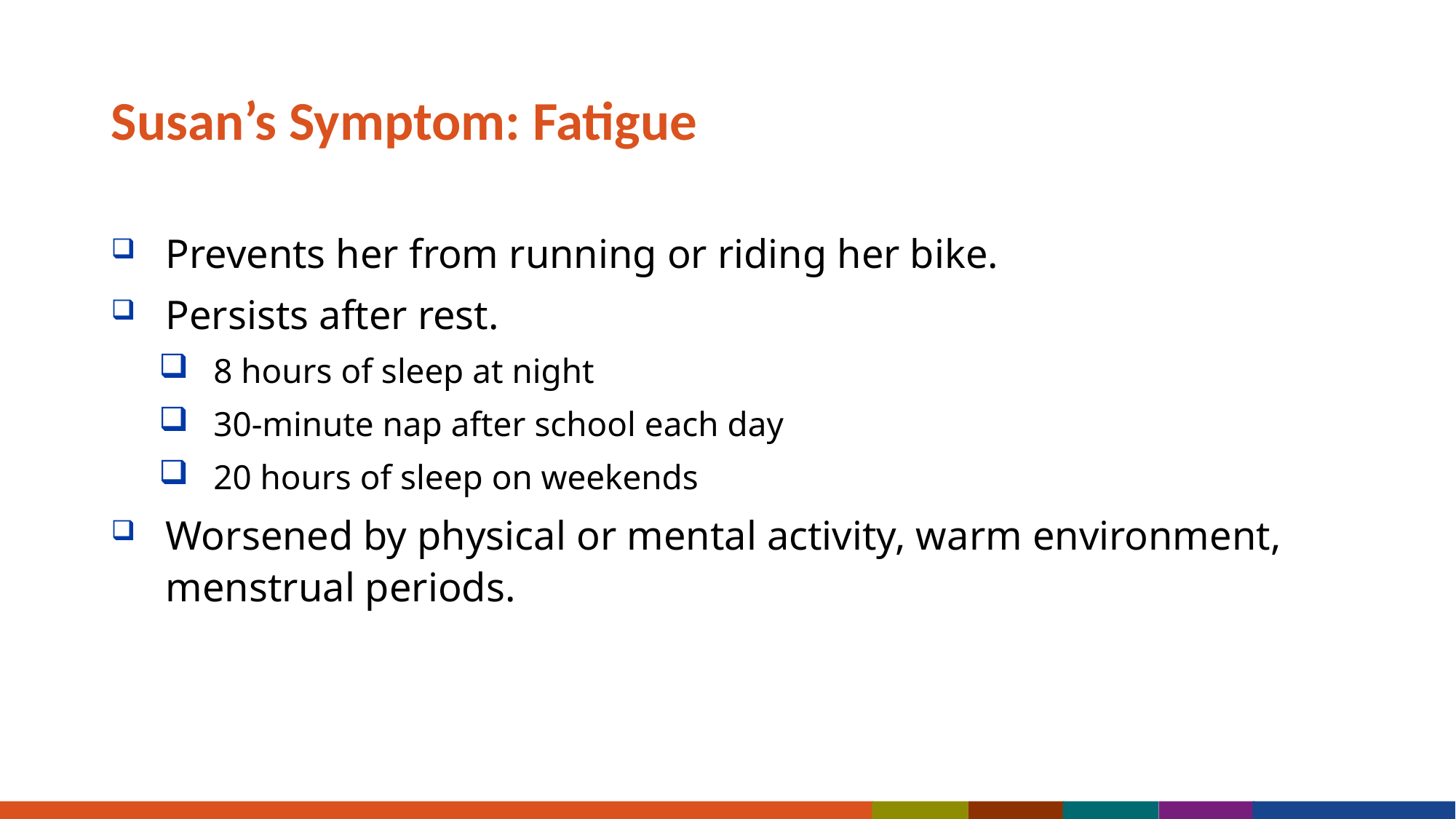

# Susan’s Symptom: Fatigue
Prevents her from running or riding her bike.
Persists after rest.
8 hours of sleep at night
30-minute nap after school each day
20 hours of sleep on weekends
Worsened by physical or mental activity, warm environment, menstrual periods.

## Slide 21
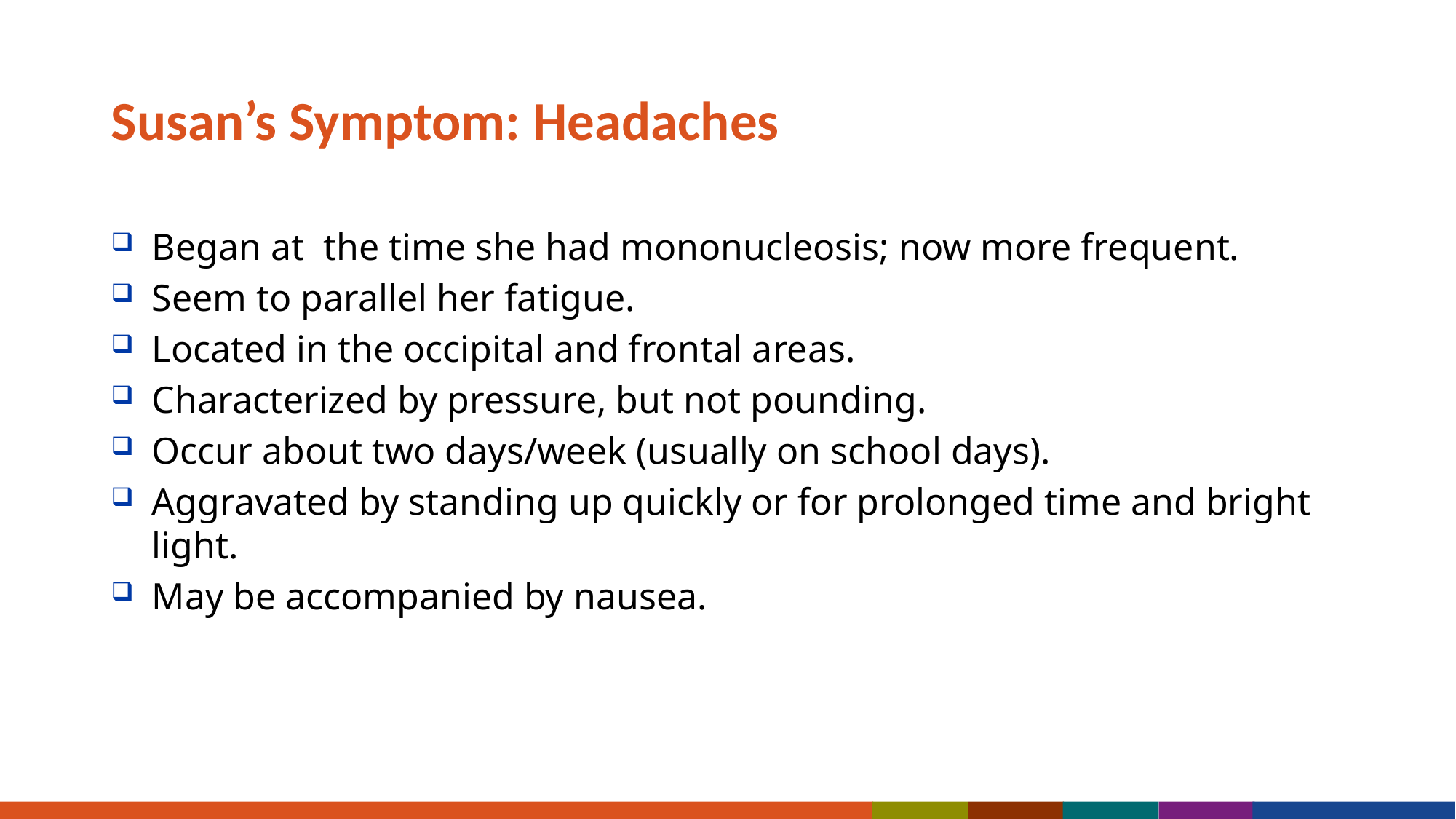

# Susan’s Symptom: Headaches
Began at the time she had mononucleosis; now more frequent.
Seem to parallel her fatigue.
Located in the occipital and frontal areas.
Characterized by pressure, but not pounding.
Occur about two days/week (usually on school days).
Aggravated by standing up quickly or for prolonged time and bright light.
May be accompanied by nausea.

## Slide 22
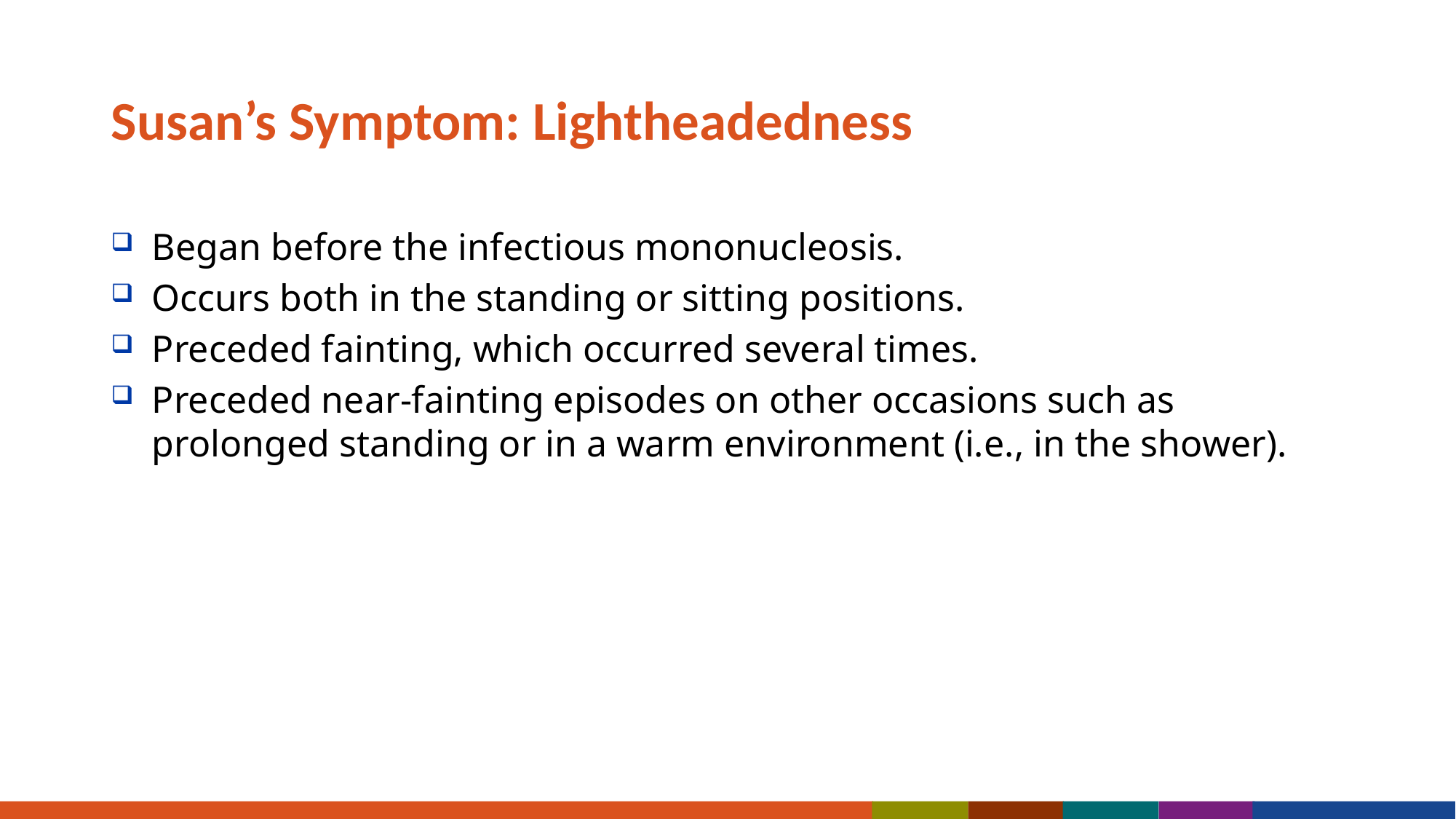

# Susan’s Symptom: Lightheadedness
Began before the infectious mononucleosis.
Occurs both in the standing or sitting positions.
Preceded fainting, which occurred several times.
Preceded near-fainting episodes on other occasions such as prolonged standing or in a warm environment (i.e., in the shower).

## Slide 23
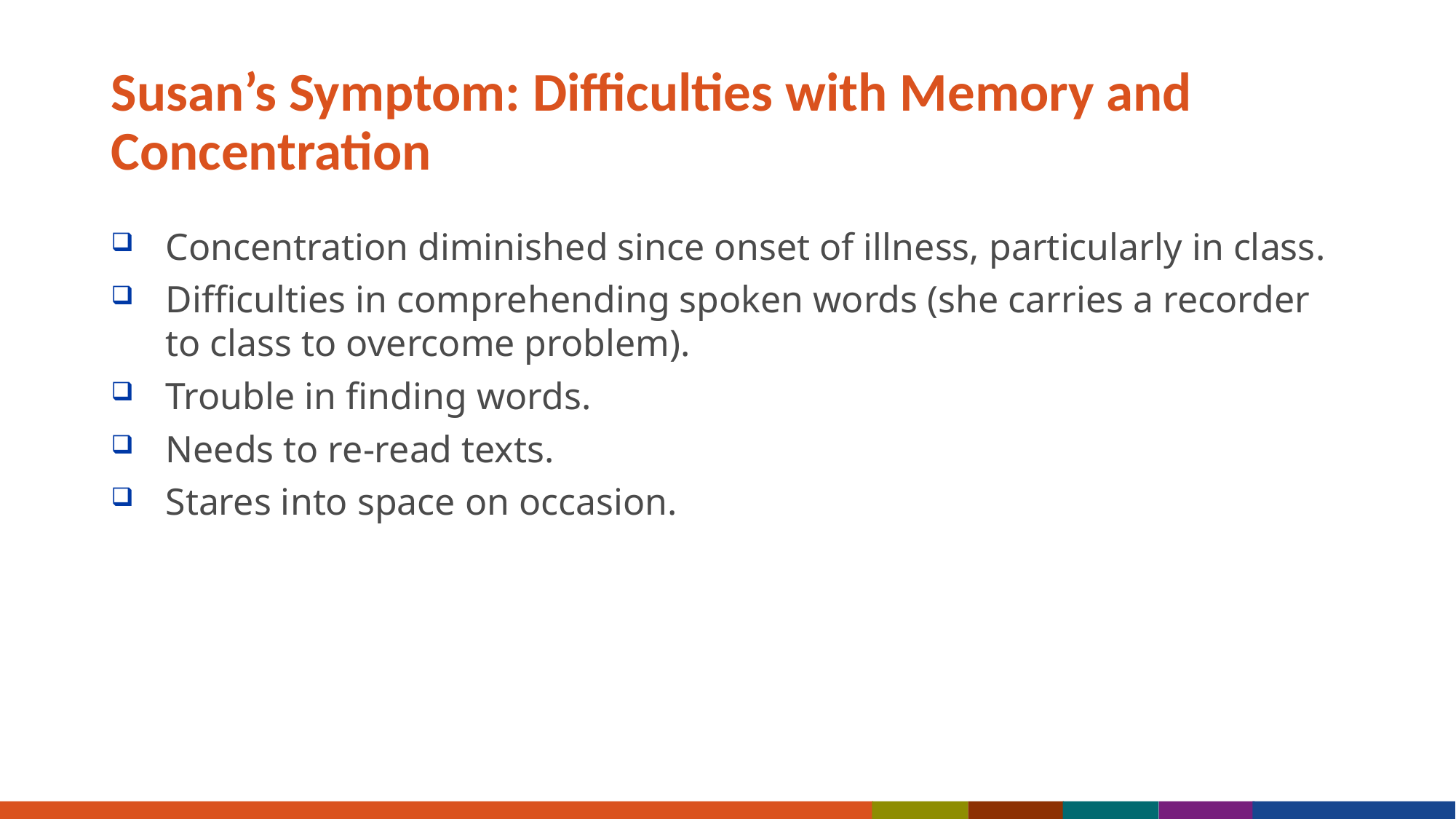

# Susan’s Symptom: Difficulties with Memory and Concentration
Concentration diminished since onset of illness, particularly in class.
Difficulties in comprehending spoken words (she carries a recorder to class to overcome problem).
Trouble in finding words.
Needs to re-read texts.
Stares into space on occasion.

## Slide 24
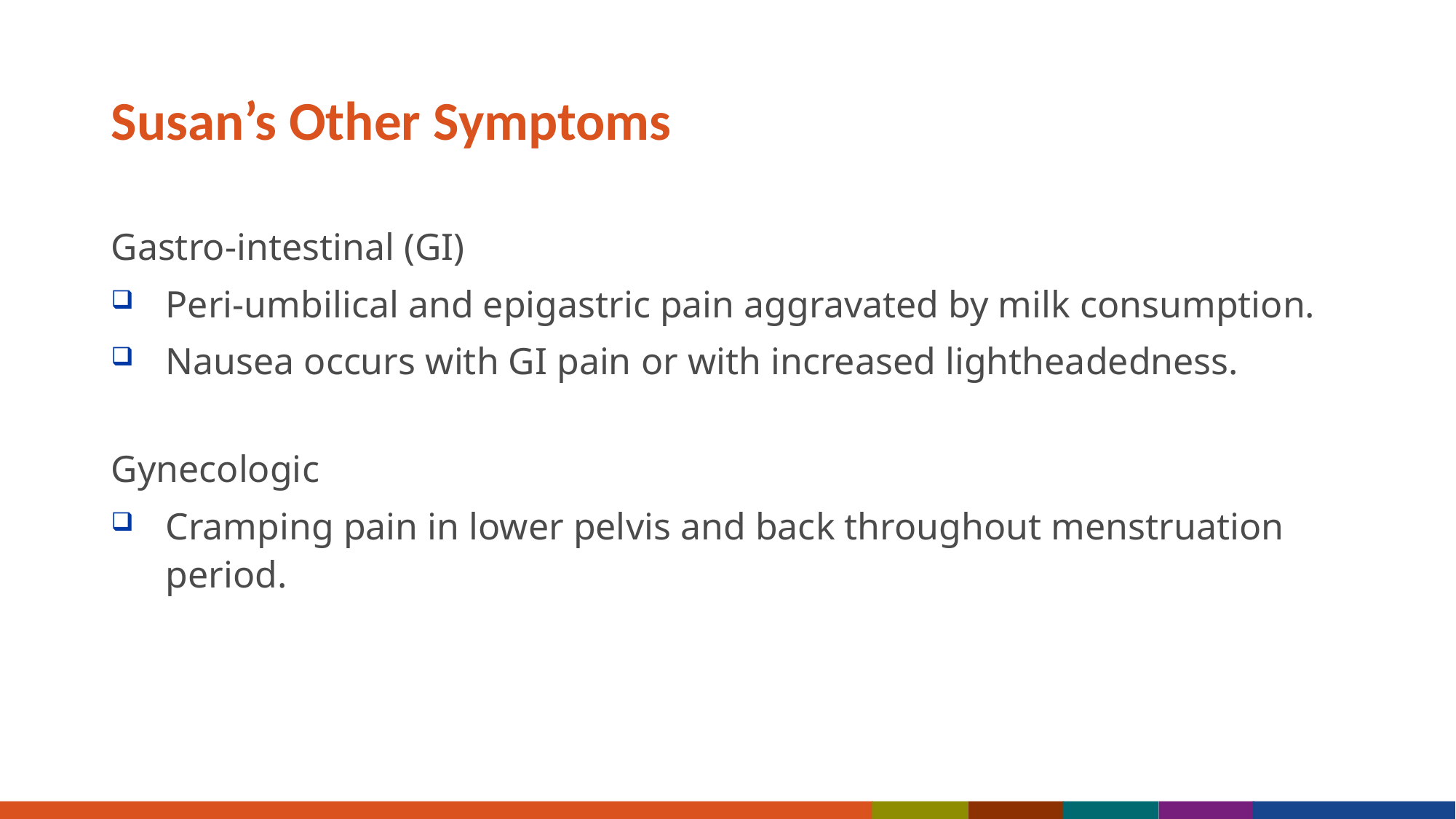

# Susan’s Other Symptoms
Gastro-intestinal (GI)
Peri-umbilical and epigastric pain aggravated by milk consumption.
Nausea occurs with GI pain or with increased lightheadedness.
Gynecologic
Cramping pain in lower pelvis and back throughout menstruation period.

## Slide 25
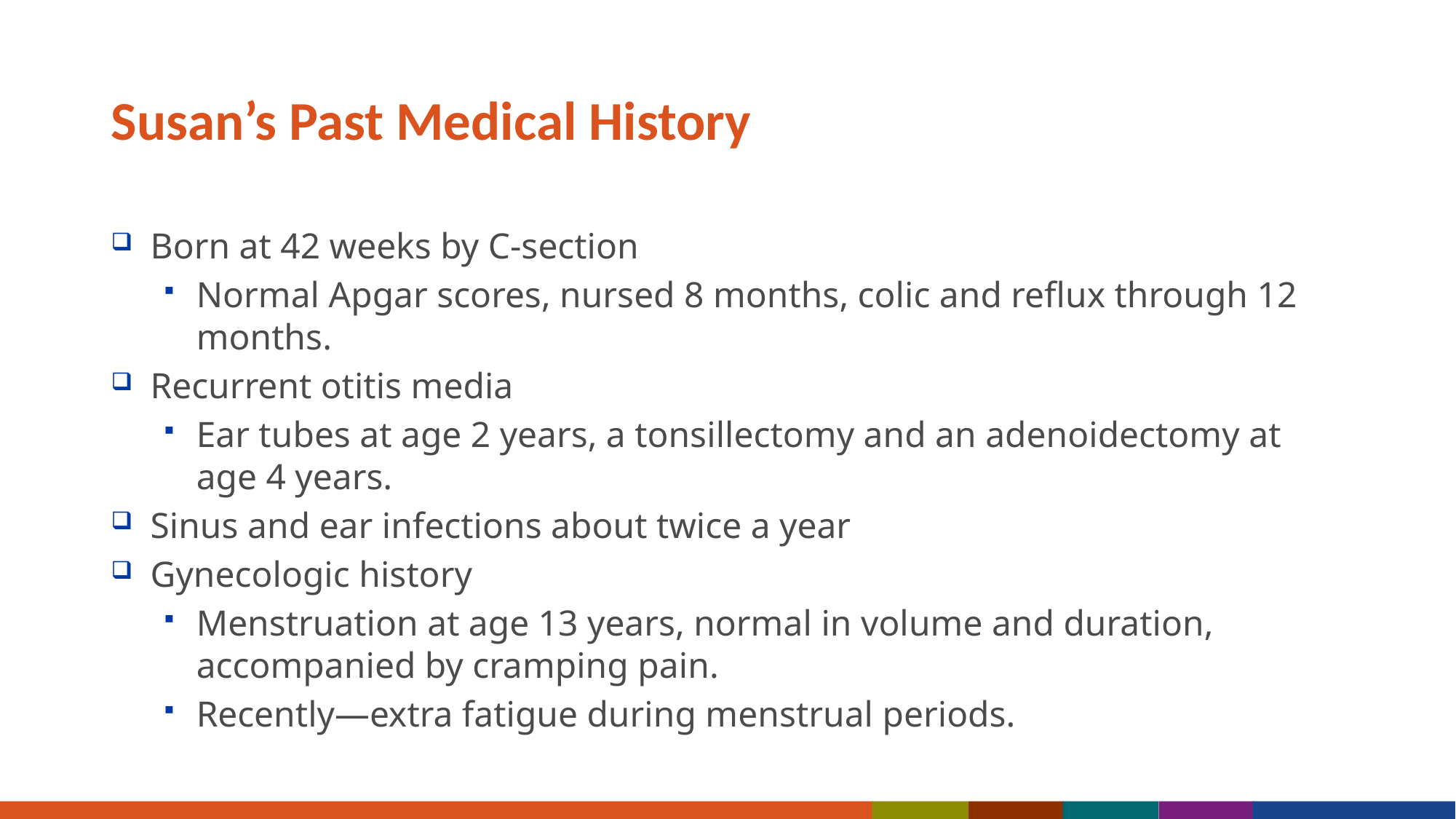

# Susan’s Past Medical History
Born at 42 weeks by C-section
Normal Apgar scores, nursed 8 months, colic and reflux through 12 months.
Recurrent otitis media
Ear tubes at age 2 years, a tonsillectomy and an adenoidectomy at age 4 years.
Sinus and ear infections about twice a year
Gynecologic history
Menstruation at age 13 years, normal in volume and duration, accompanied by cramping pain.
Recently—extra fatigue during menstrual periods.

## Slide 26
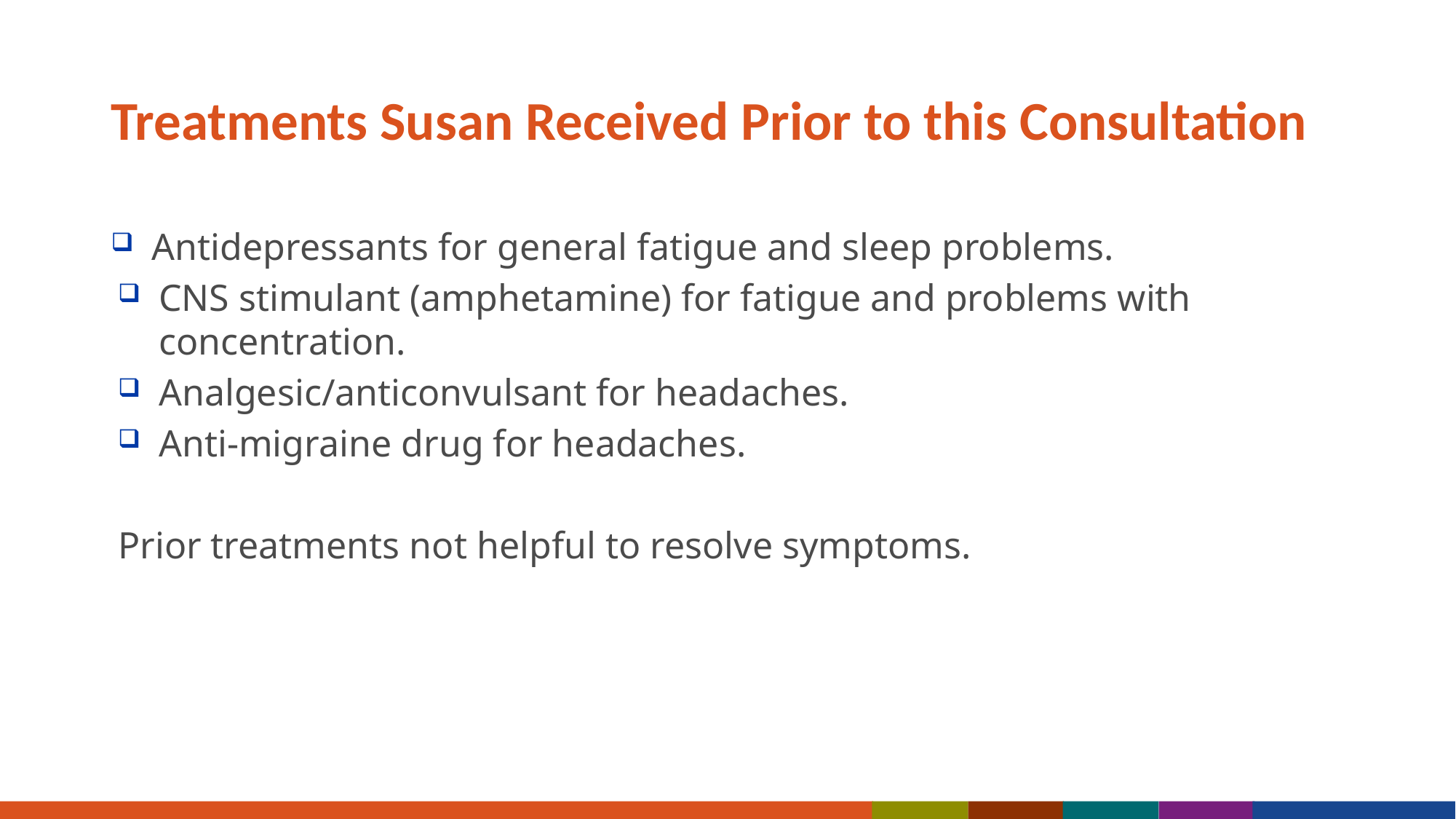

# Treatments Susan Received Prior to this Consultation
Antidepressants for general fatigue and sleep problems.
CNS stimulant (amphetamine) for fatigue and problems with concentration.
Analgesic/anticonvulsant for headaches.
Anti-migraine drug for headaches.
Prior treatments not helpful to resolve symptoms.

## Slide 27
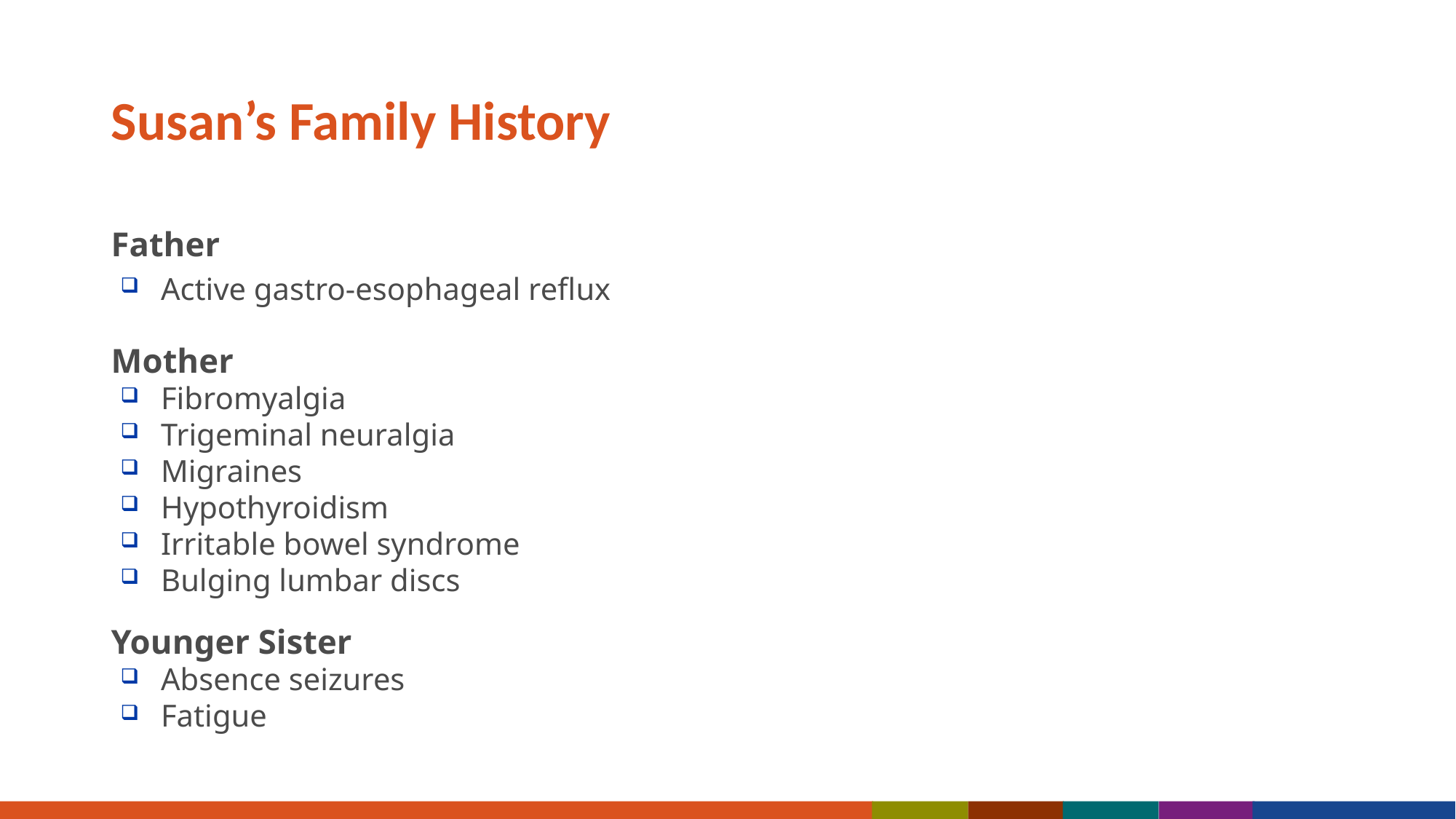

# Susan’s Family History
Father
Active gastro-esophageal reflux
Mother
Fibromyalgia
Trigeminal neuralgia
Migraines
Hypothyroidism
Irritable bowel syndrome
Bulging lumbar discs
Younger Sister
Absence seizures
Fatigue

## Slide 28
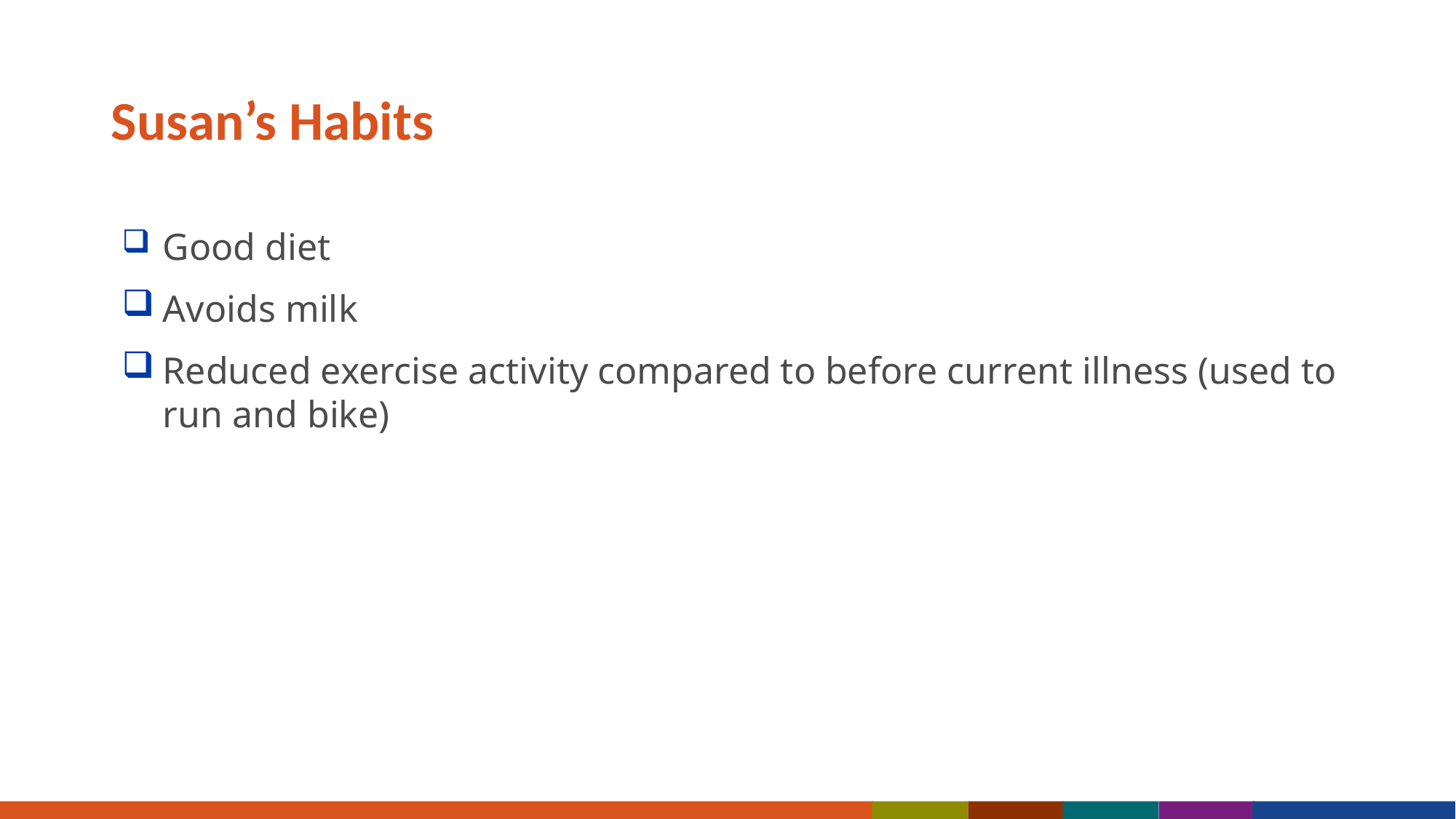

# Susan’s Habits
Good diet
Avoids milk
Reduced exercise activity compared to before current illness (used to run and bike)

## Slide 29
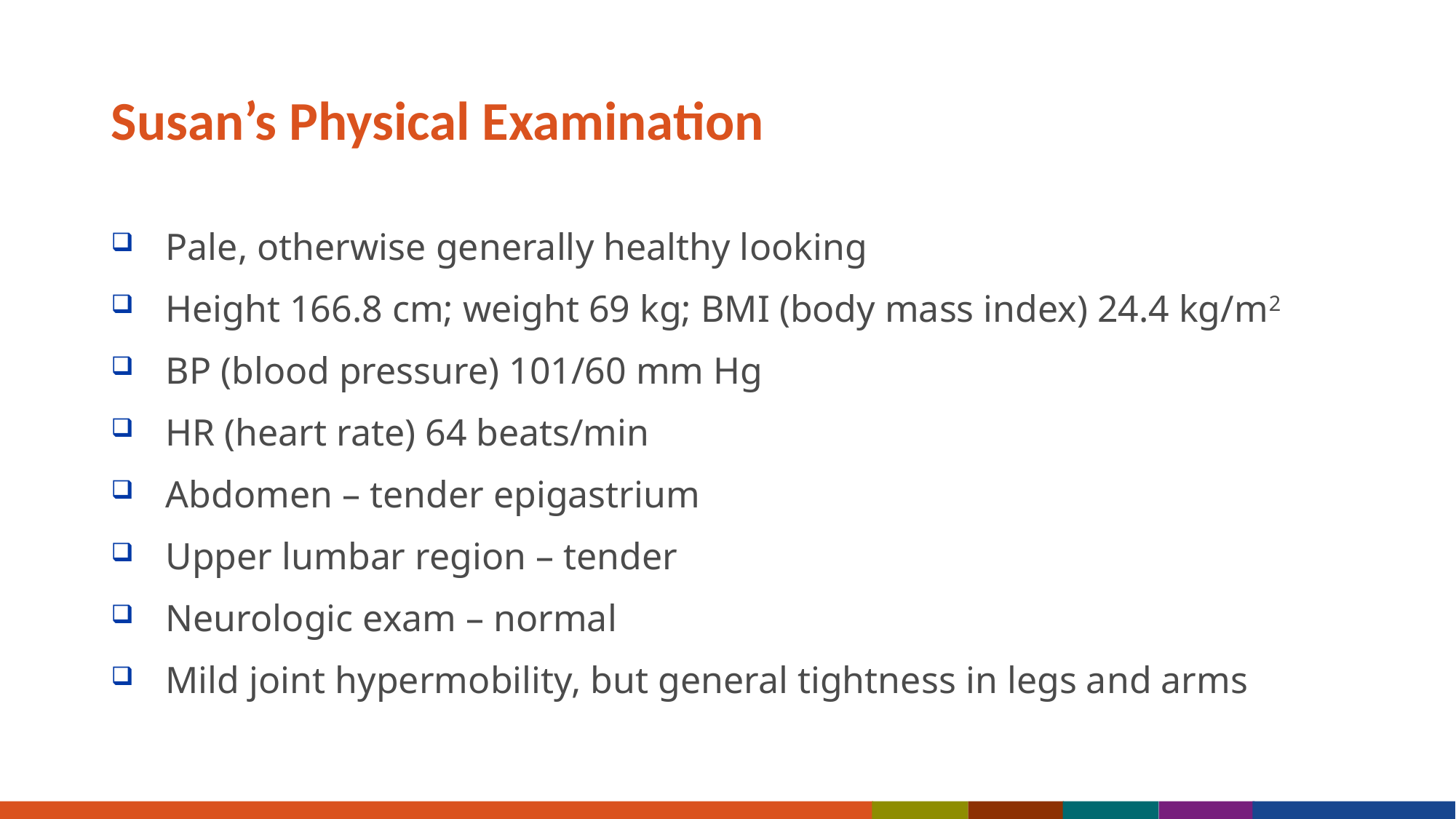

# Susan’s Physical Examination
Pale, otherwise generally healthy looking
Height 166.8 cm; weight 69 kg; BMI (body mass index) 24.4 kg/m2
BP (blood pressure) 101/60 mm Hg
HR (heart rate) 64 beats/min
Abdomen – tender epigastrium
Upper lumbar region – tender
Neurologic exam – normal
Mild joint hypermobility, but general tightness in legs and arms

## Slide 30
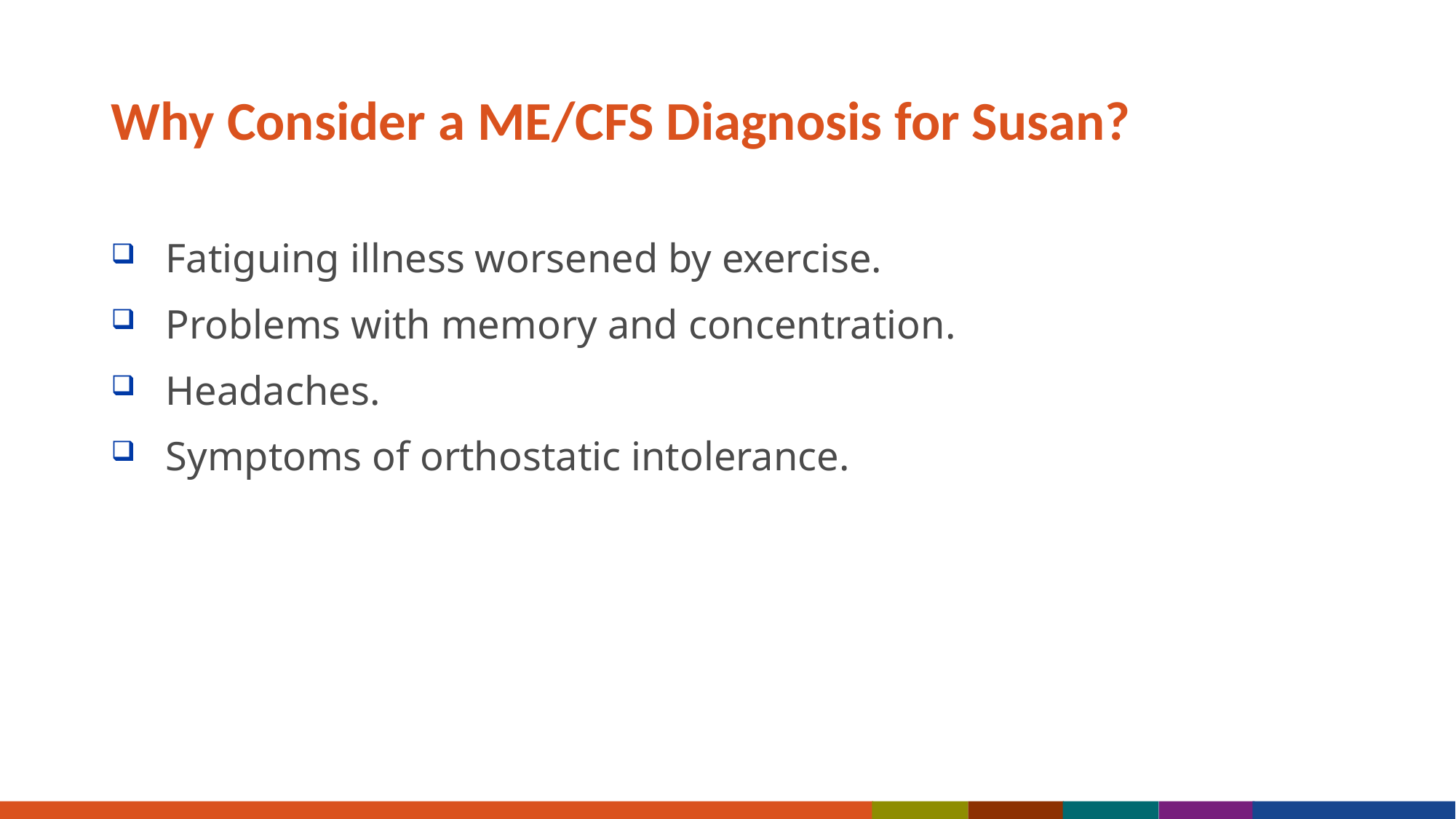

# Why Consider a ME/CFS Diagnosis for Susan?
Fatiguing illness worsened by exercise.
Problems with memory and concentration.
Headaches.
Symptoms of orthostatic intolerance.

## Slide 31
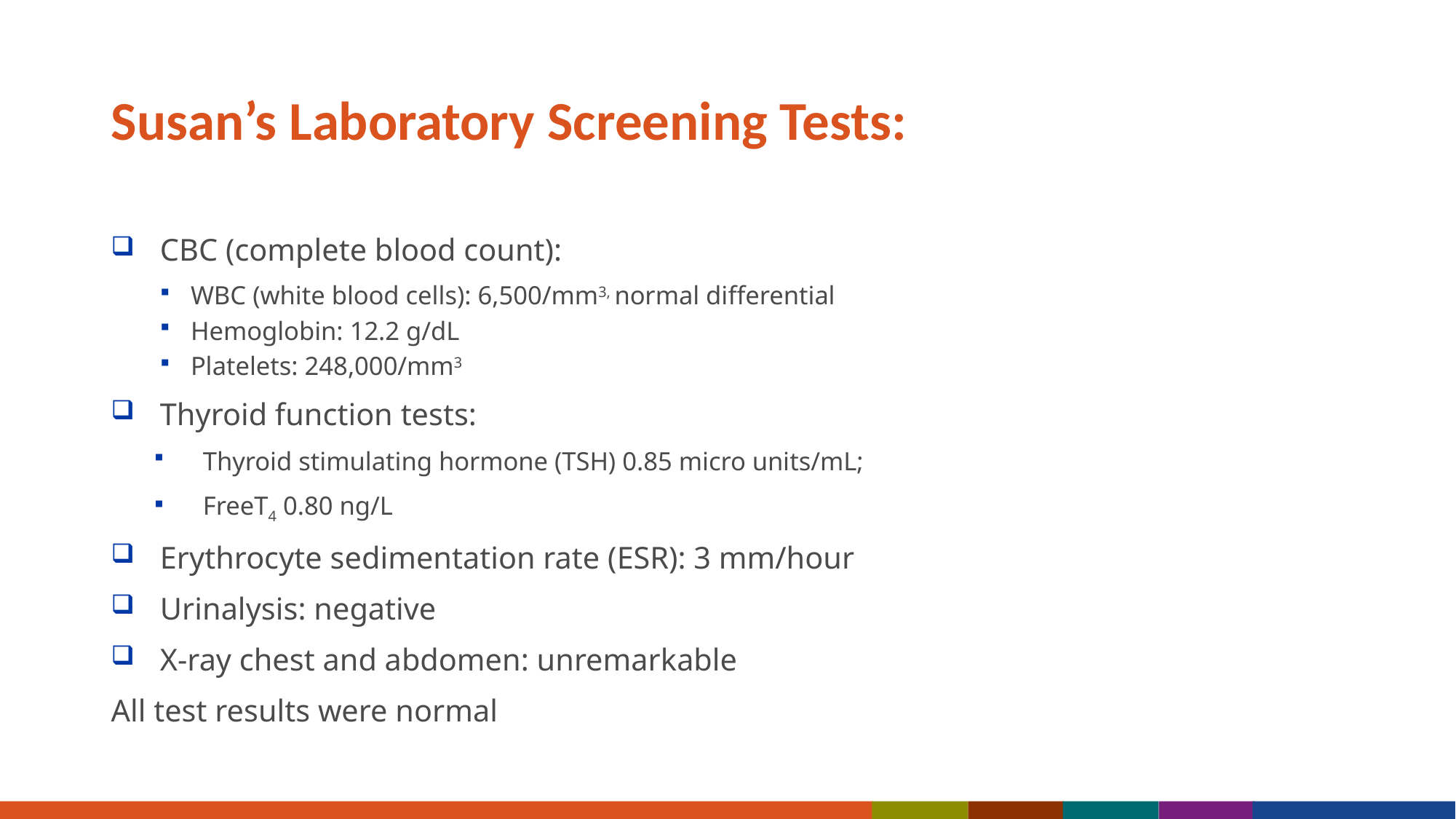

# Susan’s Laboratory Screening Tests:
CBC (complete blood count):
WBC (white blood cells): 6,500/mm3, normal differential
Hemoglobin: 12.2 g/dL
Platelets: 248,000/mm3
Thyroid function tests:
Thyroid stimulating hormone (TSH) 0.85 micro units/mL;
FreeT4 0.80 ng/L
Erythrocyte sedimentation rate (ESR): 3 mm/hour
Urinalysis: negative
X-ray chest and abdomen: unremarkable
All test results were normal

## Slide 32
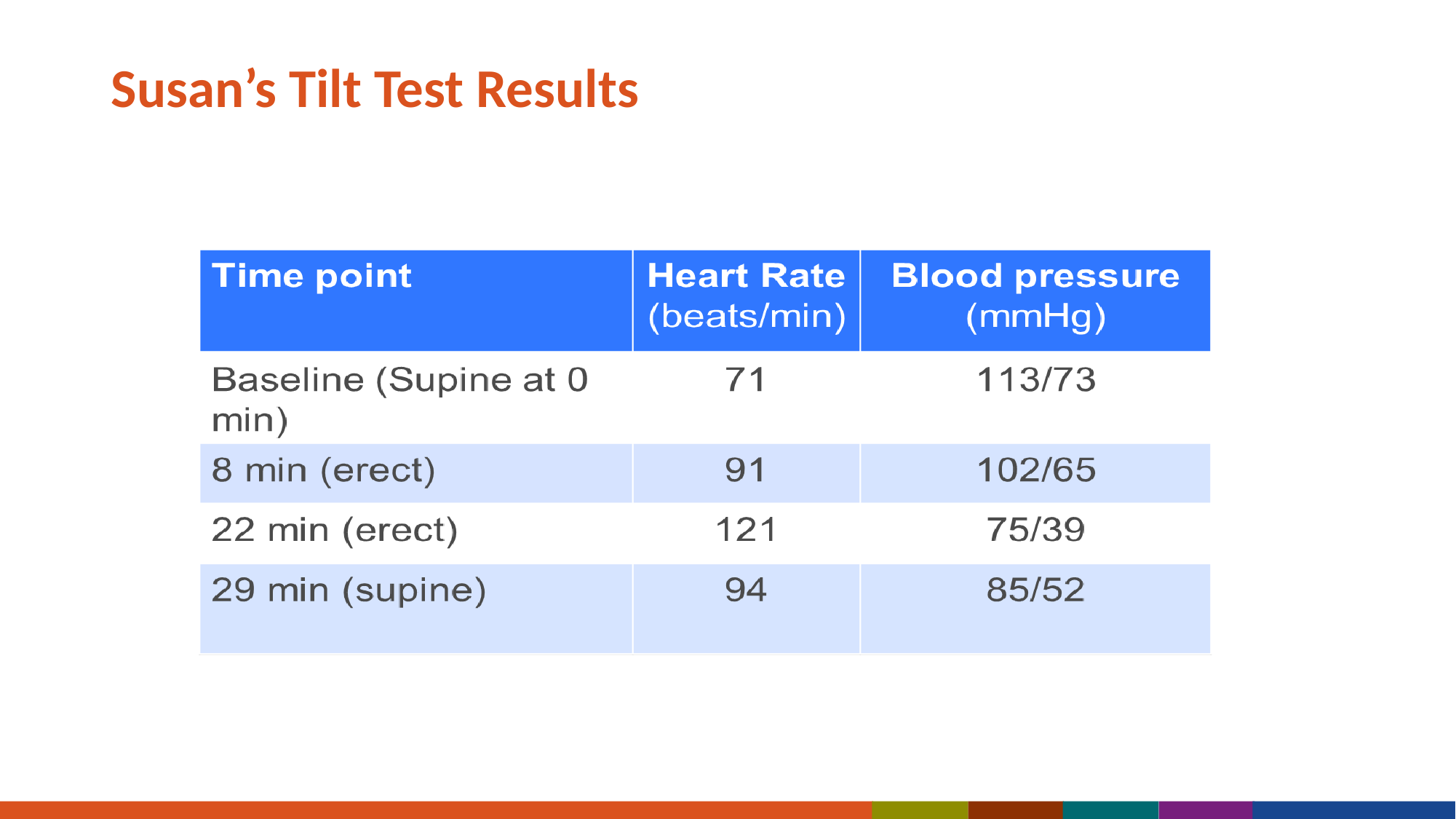

# Susan’s Tilt Test Results

## Slide 33
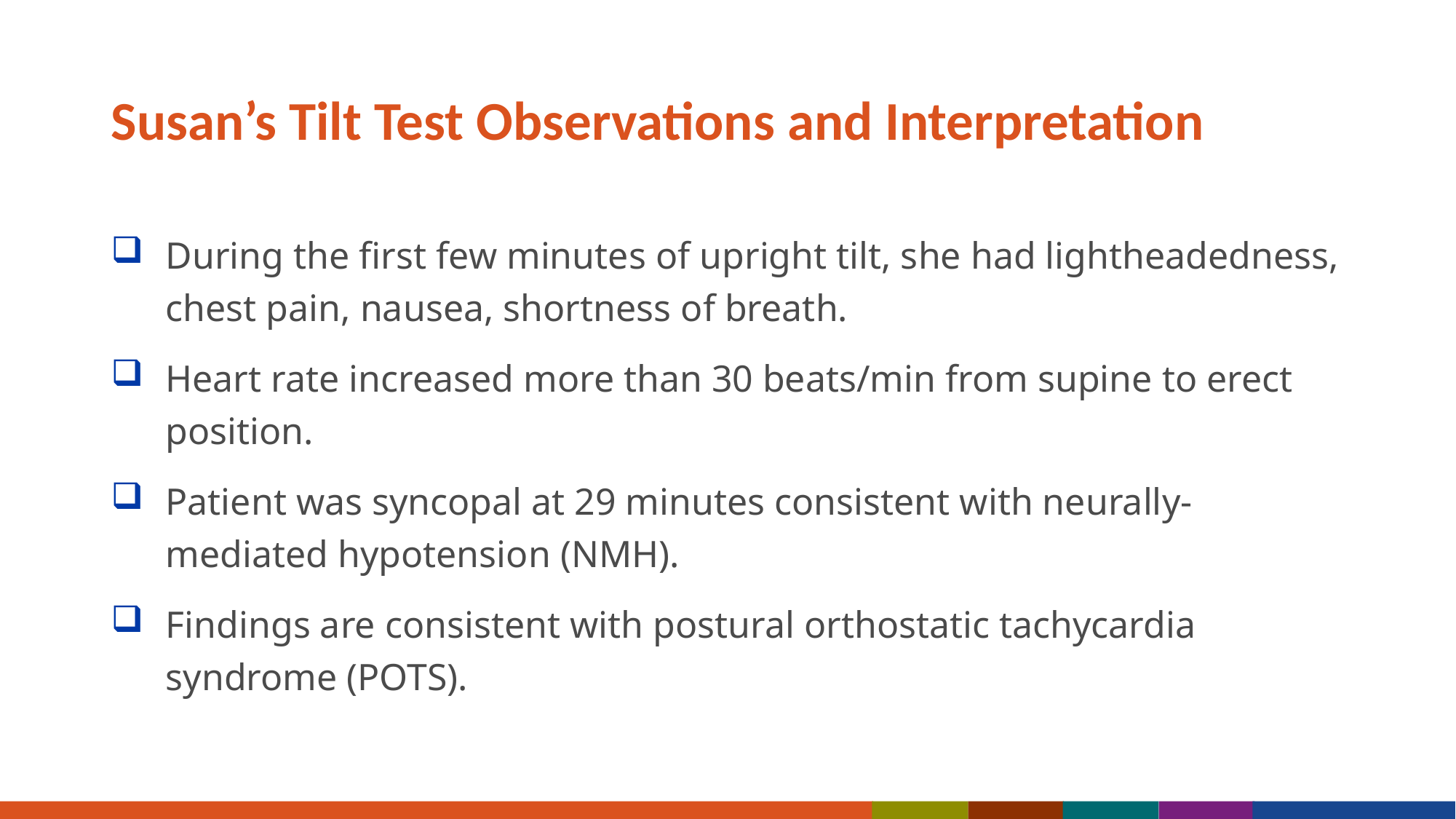

# Susan’s Tilt Test Observations and Interpretation
During the first few minutes of upright tilt, she had lightheadedness, chest pain, nausea, shortness of breath.
Heart rate increased more than 30 beats/min from supine to erect position.
Patient was syncopal at 29 minutes consistent with neurally-mediated hypotension (NMH).
Findings are consistent with postural orthostatic tachycardia syndrome (POTS).

## Slide 34
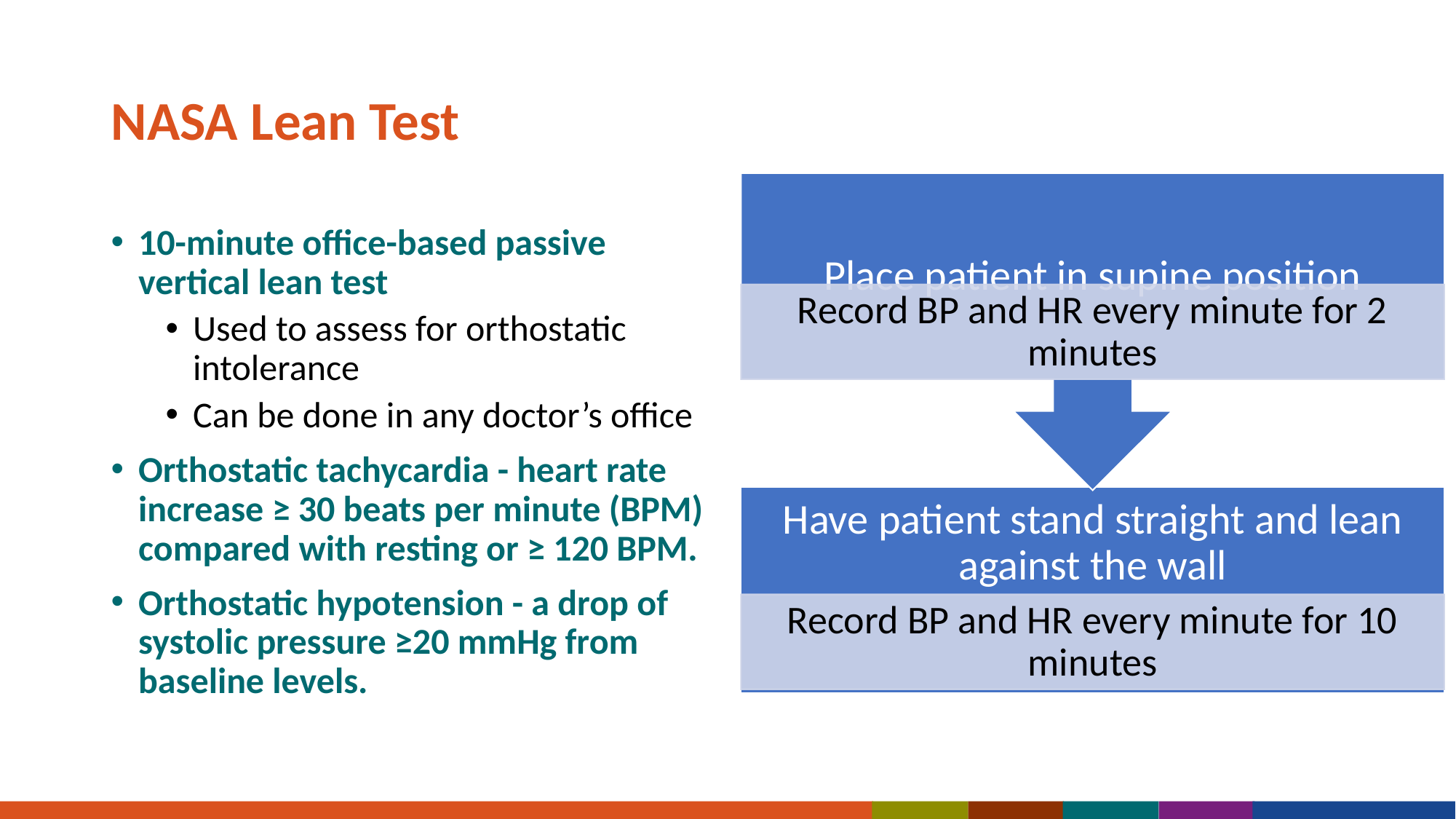

# NASA Lean Test
10-minute office-based passive vertical lean test
Used to assess for orthostatic intolerance
Can be done in any doctor’s office
Orthostatic tachycardia - heart rate increase ≥ 30 beats per minute (BPM) compared with resting or ≥ 120 BPM.
Orthostatic hypotension - a drop of systolic pressure ≥20 mmHg from baseline levels.

## Slide 35
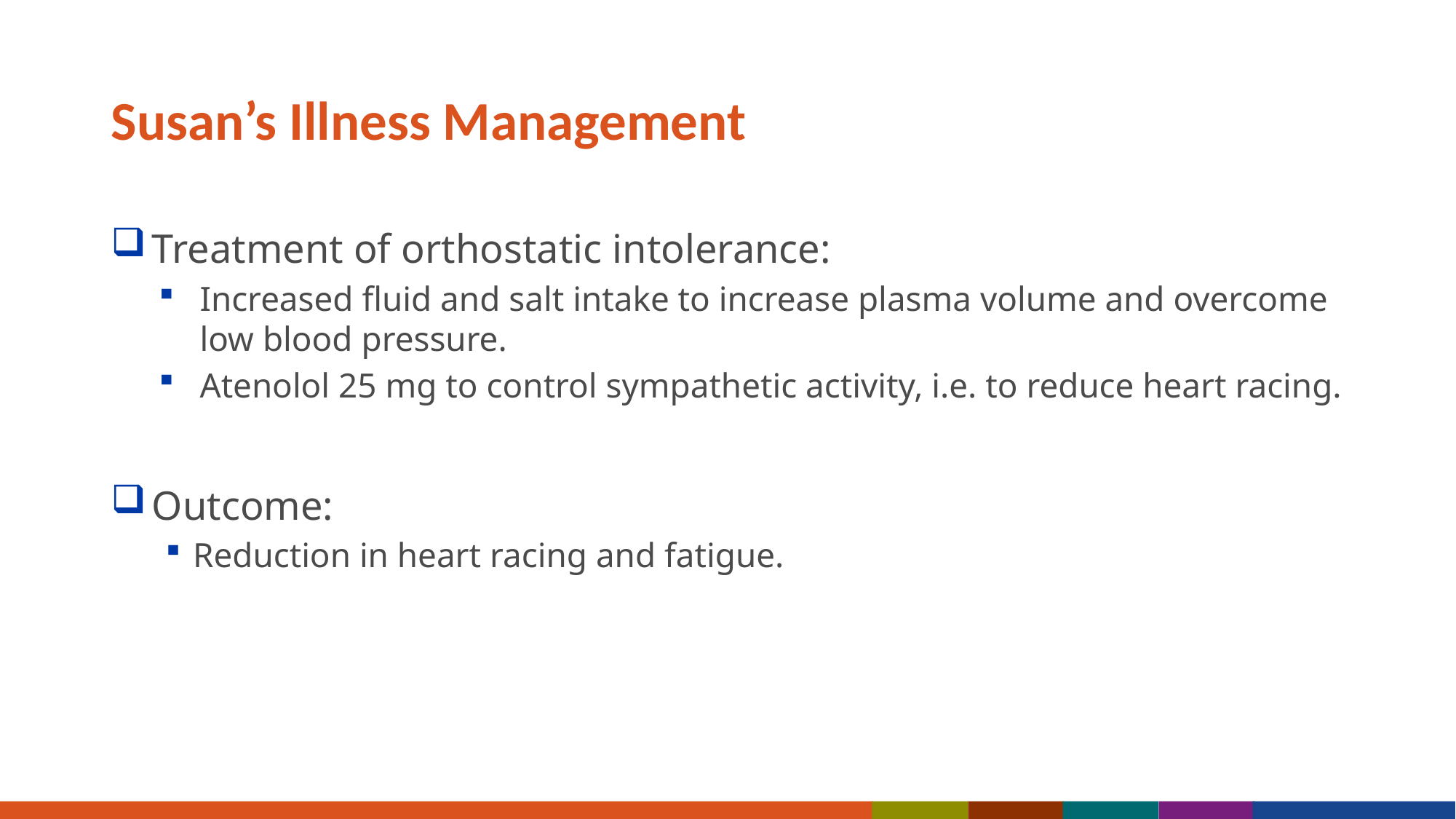

# Susan’s Illness Management
Treatment of orthostatic intolerance:
Increased fluid and salt intake to increase plasma volume and overcome low blood pressure.
Atenolol 25 mg to control sympathetic activity, i.e. to reduce heart racing.
Outcome:
Reduction in heart racing and fatigue.

## Slide 36
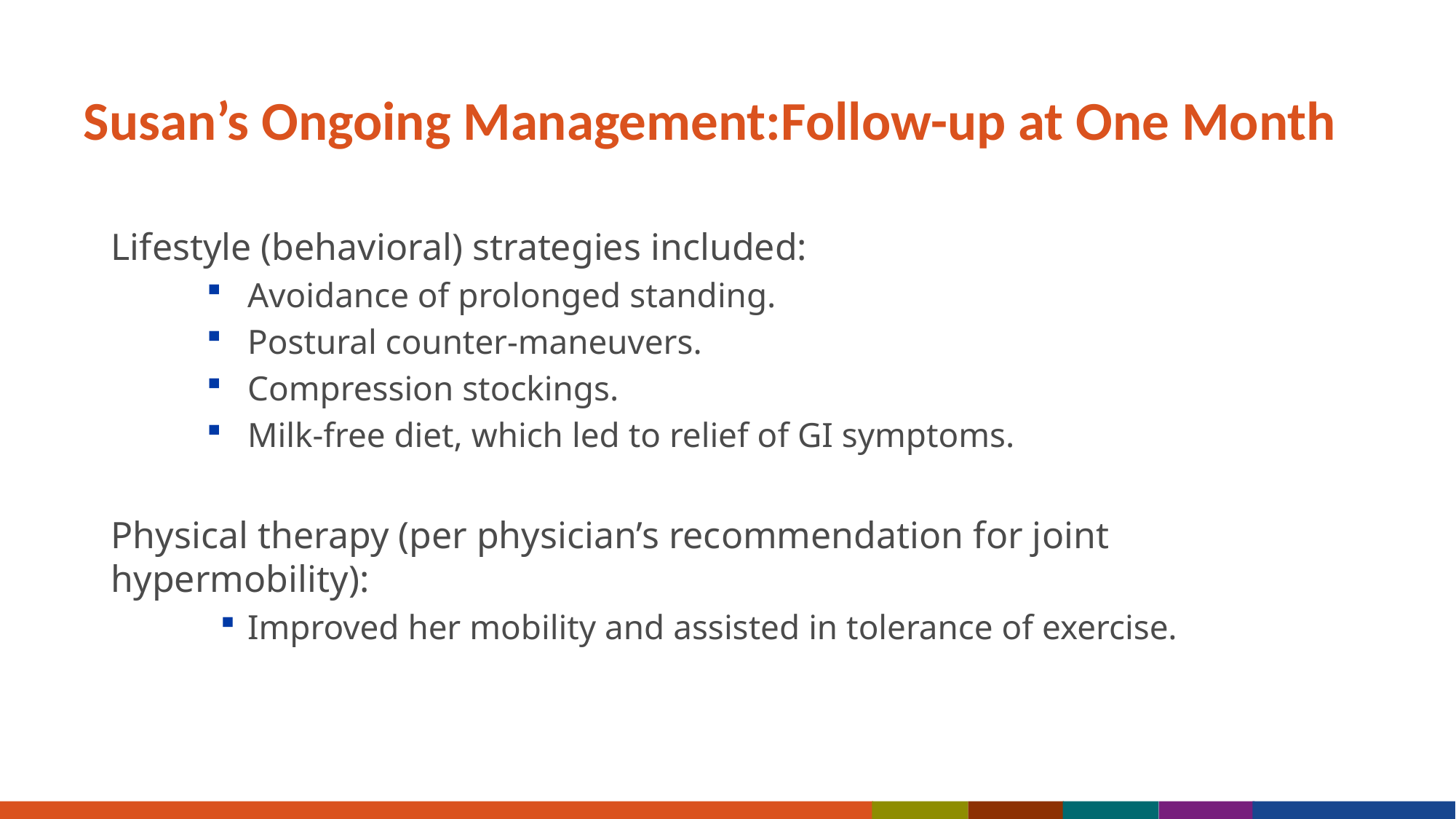

# Susan’s Ongoing Management:Follow-up at One Month
Lifestyle (behavioral) strategies included:
Avoidance of prolonged standing.
Postural counter-maneuvers.
Compression stockings.
Milk-free diet, which led to relief of GI symptoms.
Physical therapy (per physician’s recommendation for joint hypermobility):
Improved her mobility and assisted in tolerance of exercise.

## Slide 37
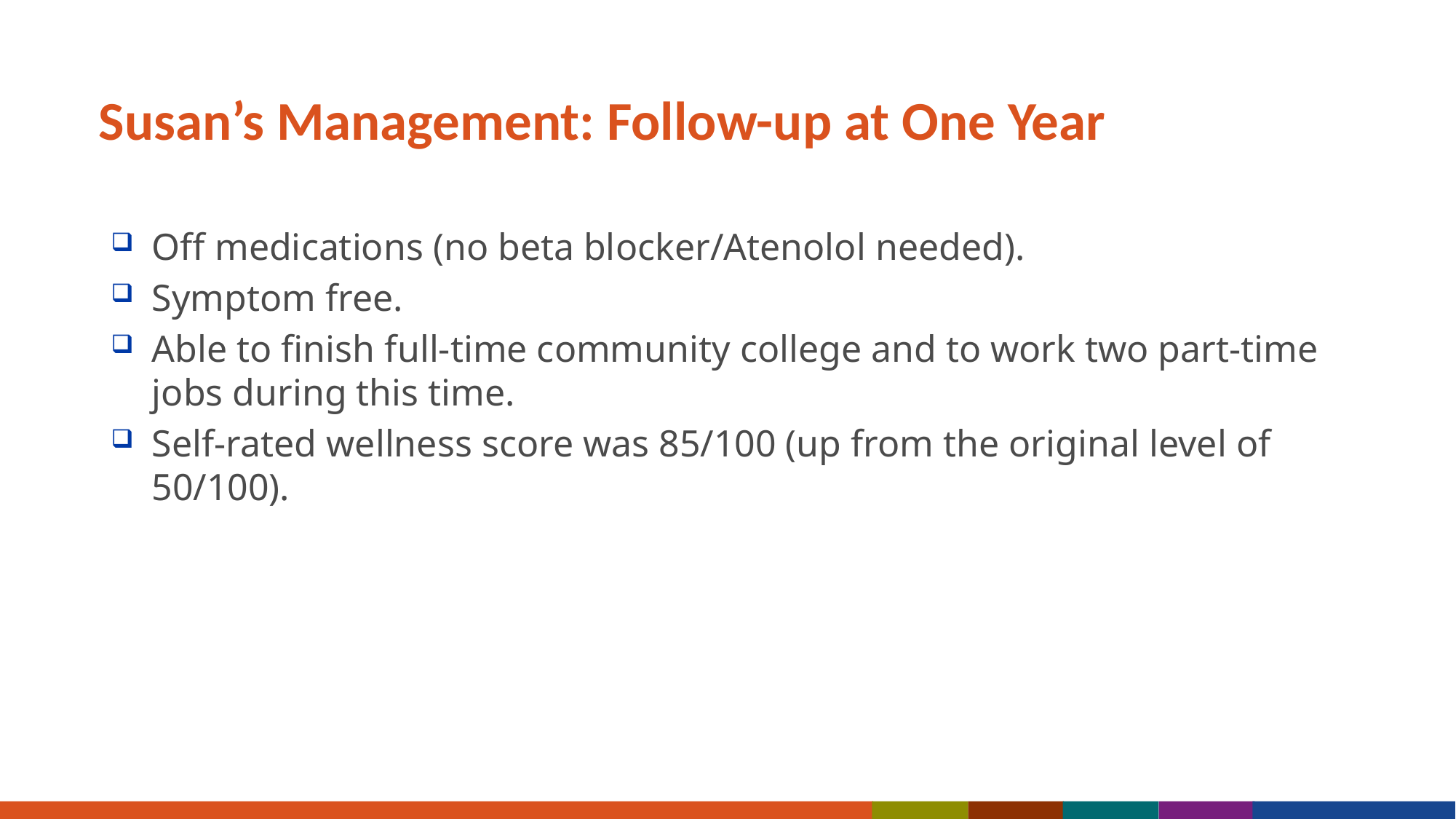

# Susan’s Management: Follow-up at One Year
Off medications (no beta blocker/Atenolol needed).
Symptom free.
Able to finish full-time community college and to work two part-time jobs during this time.
Self-rated wellness score was 85/100 (up from the original level of 50/100).

## Slide 38
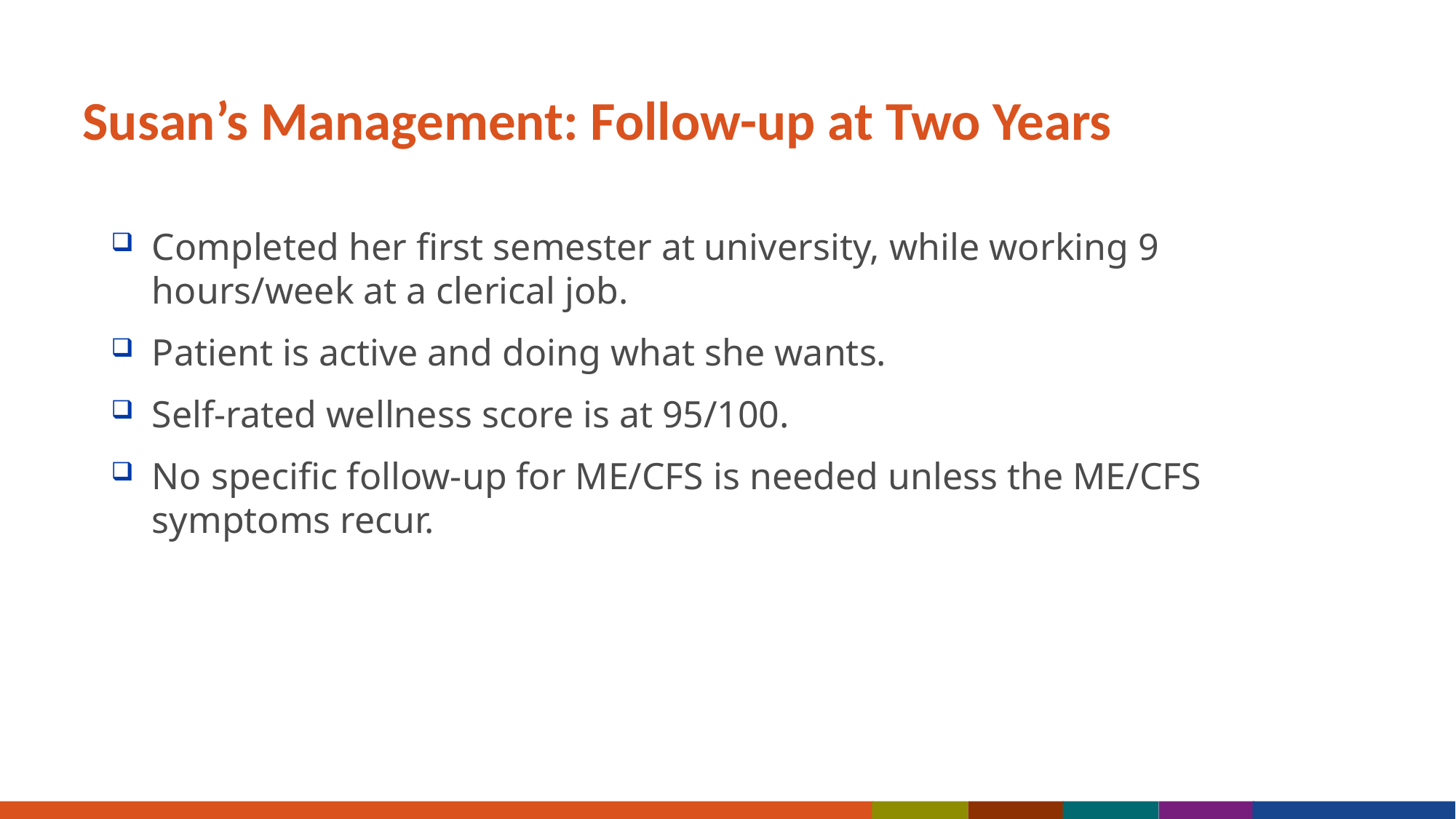

# Susan’s Management: Follow-up at Two Years
Completed her first semester at university, while working 9 hours/week at a clerical job.
Patient is active and doing what she wants.
Self-rated wellness score is at 95/100.
No specific follow-up for ME/CFS is needed unless the ME/CFS symptoms recur.

## Slide 39
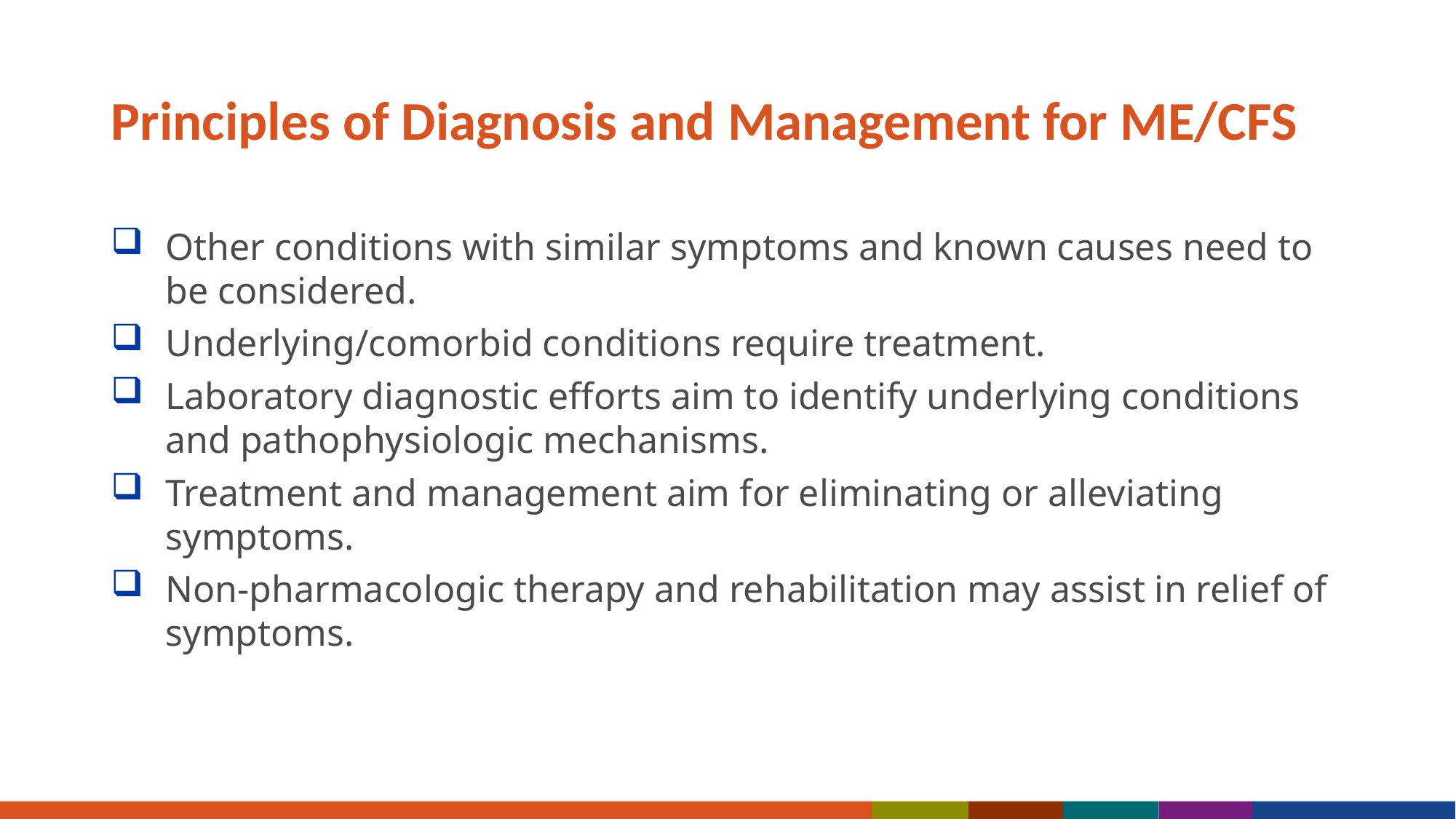

# Principles of Diagnosis and Management for ME/CFS
Other conditions with similar symptoms and known causes need to be considered.
Underlying/comorbid conditions require treatment.
Laboratory diagnostic efforts aim to identify underlying conditions and pathophysiologic mechanisms.
Treatment and management aim for eliminating or alleviating symptoms.
Non-pharmacologic therapy and rehabilitation may assist in relief of symptoms.

## Slide 40
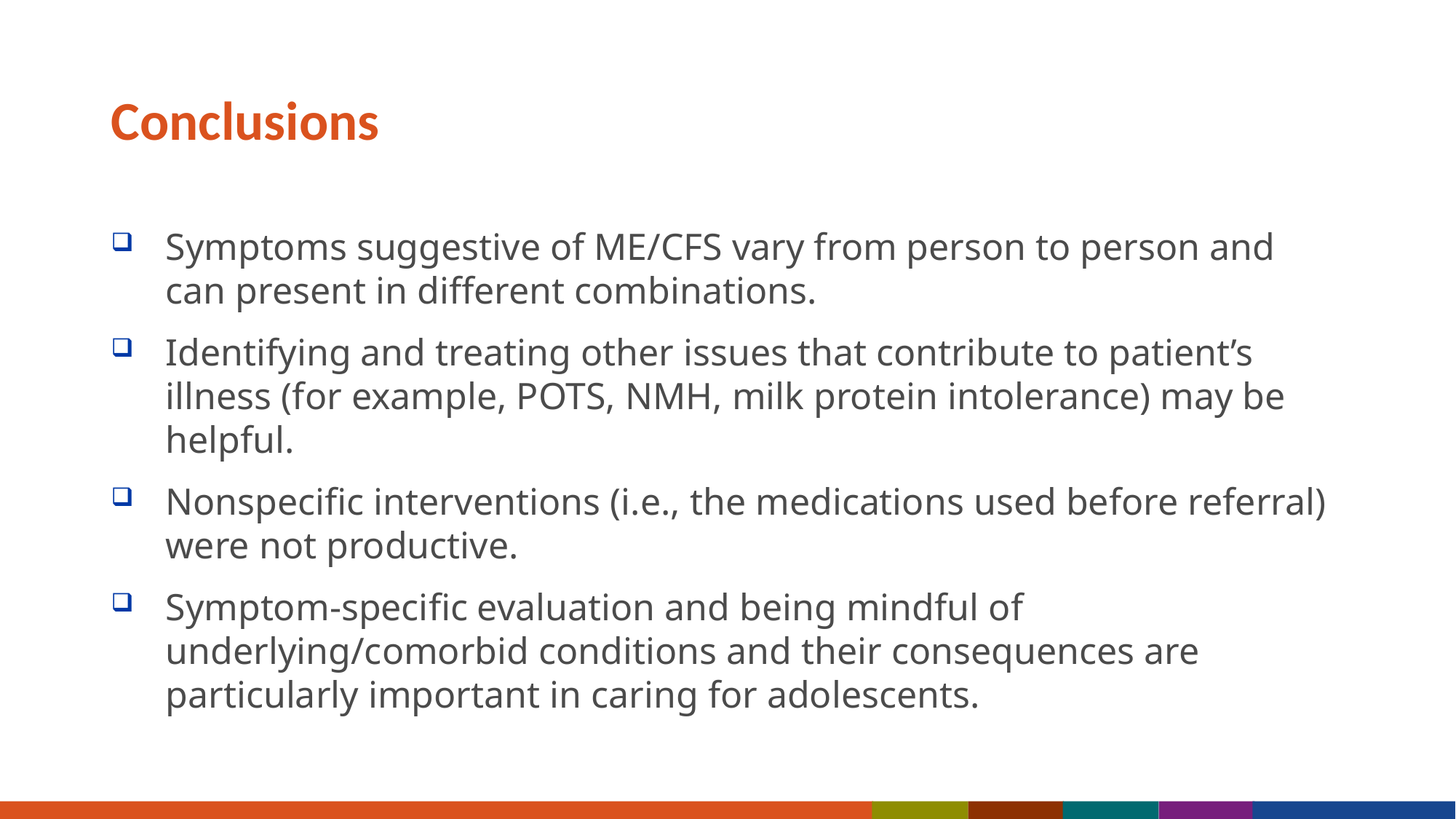

# Conclusions
Symptoms suggestive of ME/CFS vary from person to person and can present in different combinations.
Identifying and treating other issues that contribute to patient’s illness (for example, POTS, NMH, milk protein intolerance) may be helpful.
Nonspecific interventions (i.e., the medications used before referral) were not productive.
Symptom-specific evaluation and being mindful of underlying/comorbid conditions and their consequences are particularly important in caring for adolescents.

## Slide 41
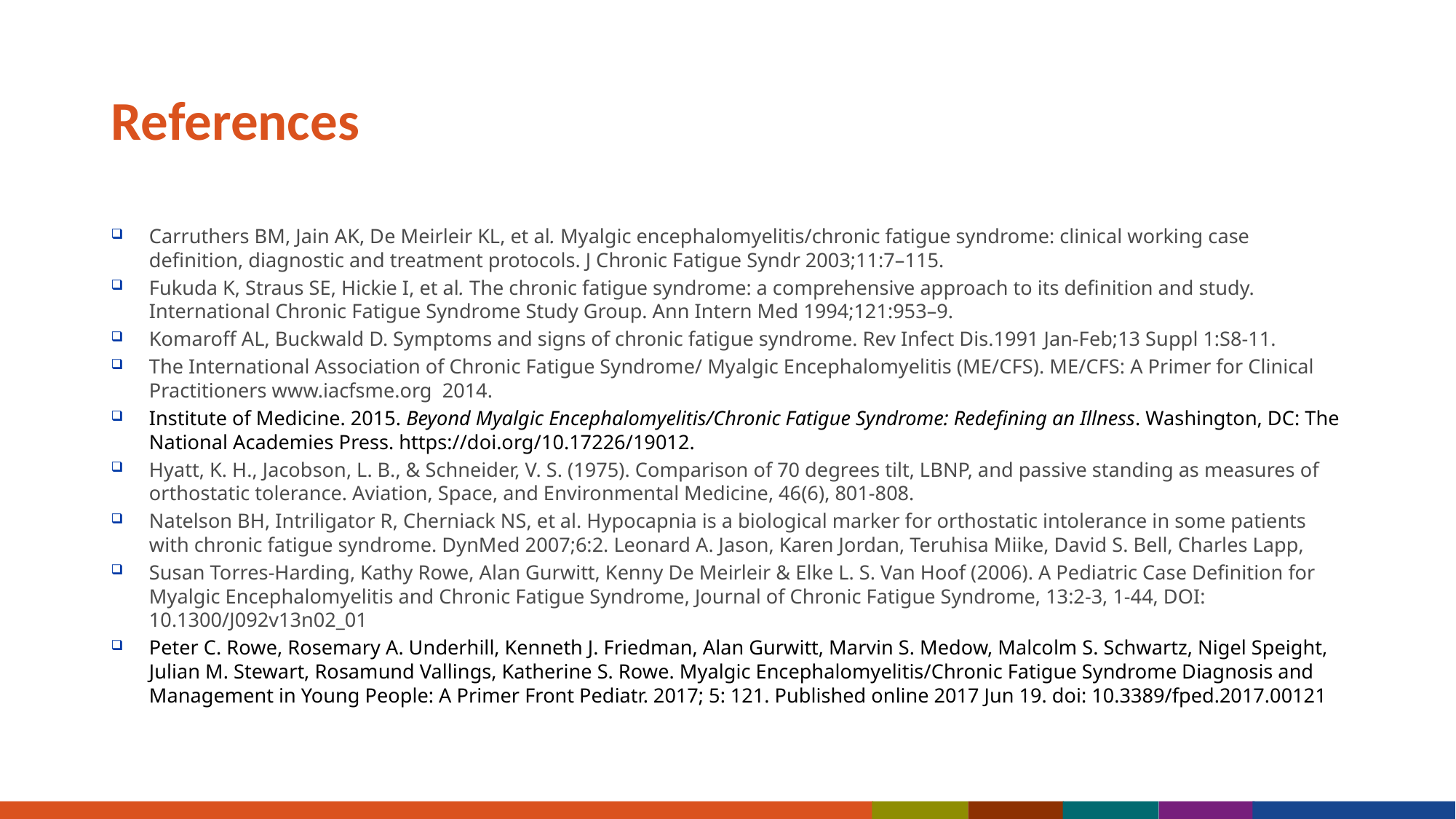

# References
Carruthers BM, Jain AK, De Meirleir KL, et al. Myalgic encephalomyelitis/chronic fatigue syndrome: clinical working case definition, diagnostic and treatment protocols. J Chronic Fatigue Syndr 2003;11:7–115.
Fukuda K, Straus SE, Hickie I, et al. The chronic fatigue syndrome: a comprehensive approach to its definition and study. International Chronic Fatigue Syndrome Study Group. Ann Intern Med 1994;121:953–9.
Komaroff AL, Buckwald D. Symptoms and signs of chronic fatigue syndrome. Rev Infect Dis.1991 Jan-Feb;13 Suppl 1:S8-11.
The International Association of Chronic Fatigue Syndrome/ Myalgic Encephalomyelitis (ME/CFS). ME/CFS: A Primer for Clinical Practitioners www.iacfsme.org 2014.
Institute of Medicine. 2015. Beyond Myalgic Encephalomyelitis/Chronic Fatigue Syndrome: Redefining an Illness. Washington, DC: The National Academies Press. https://doi.org/10.17226/19012.
Hyatt, K. H., Jacobson, L. B., & Schneider, V. S. (1975). Comparison of 70 degrees tilt, LBNP, and passive standing as measures of orthostatic tolerance. Aviation, Space, and Environmental Medicine, 46(6), 801-808.
Natelson BH, Intriligator R, Cherniack NS, et al. Hypocapnia is a biological marker for orthostatic intolerance in some patients with chronic fatigue syndrome. DynMed 2007;6:2. Leonard A. Jason, Karen Jordan, Teruhisa Miike, David S. Bell, Charles Lapp,
Susan Torres-Harding, Kathy Rowe, Alan Gurwitt, Kenny De Meirleir & Elke L. S. Van Hoof (2006). A Pediatric Case Definition for Myalgic Encephalomyelitis and Chronic Fatigue Syndrome, Journal of Chronic Fatigue Syndrome, 13:2-3, 1-44, DOI: 10.1300/J092v13n02_01
Peter C. Rowe, Rosemary A. Underhill, Kenneth J. Friedman, Alan Gurwitt, Marvin S. Medow, Malcolm S. Schwartz, Nigel Speight, Julian M. Stewart, Rosamund Vallings, Katherine S. Rowe. Myalgic Encephalomyelitis/Chronic Fatigue Syndrome Diagnosis and Management in Young People: A Primer Front Pediatr. 2017; 5: 121. Published online 2017 Jun 19. doi: 10.3389/fped.2017.00121

## Slide 42
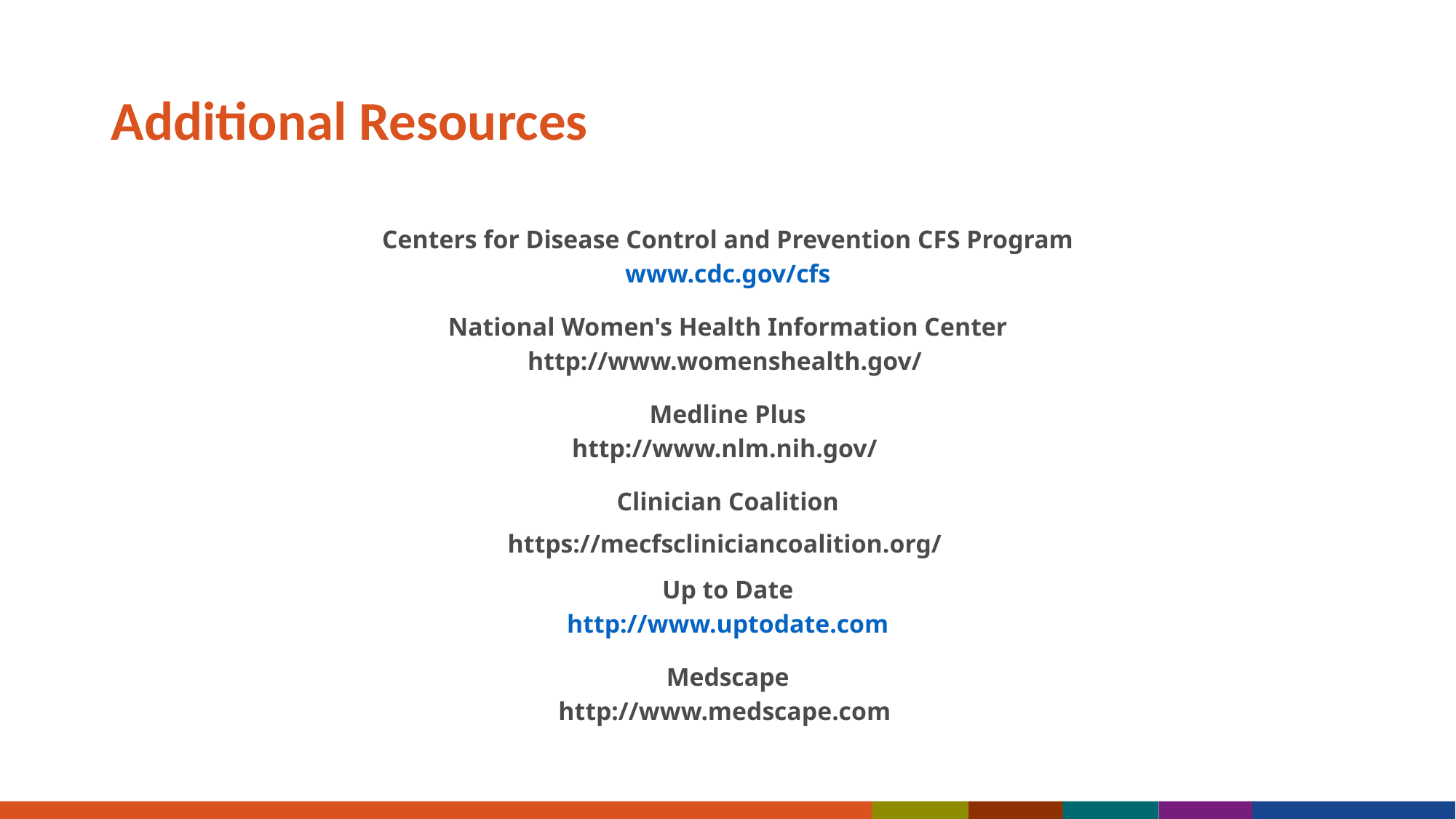

# Additional Resources
Centers for Disease Control and Prevention CFS Program
www.cdc.gov/cfs
National Women's Health Information Center
http://www.womenshealth.gov/
Medline Plus
http://www.nlm.nih.gov/
Clinician Coalition
https://mecfscliniciancoalition.org/
Up to Date
http://www.uptodate.com
Medscape
http://www.medscape.com

## Slide 43
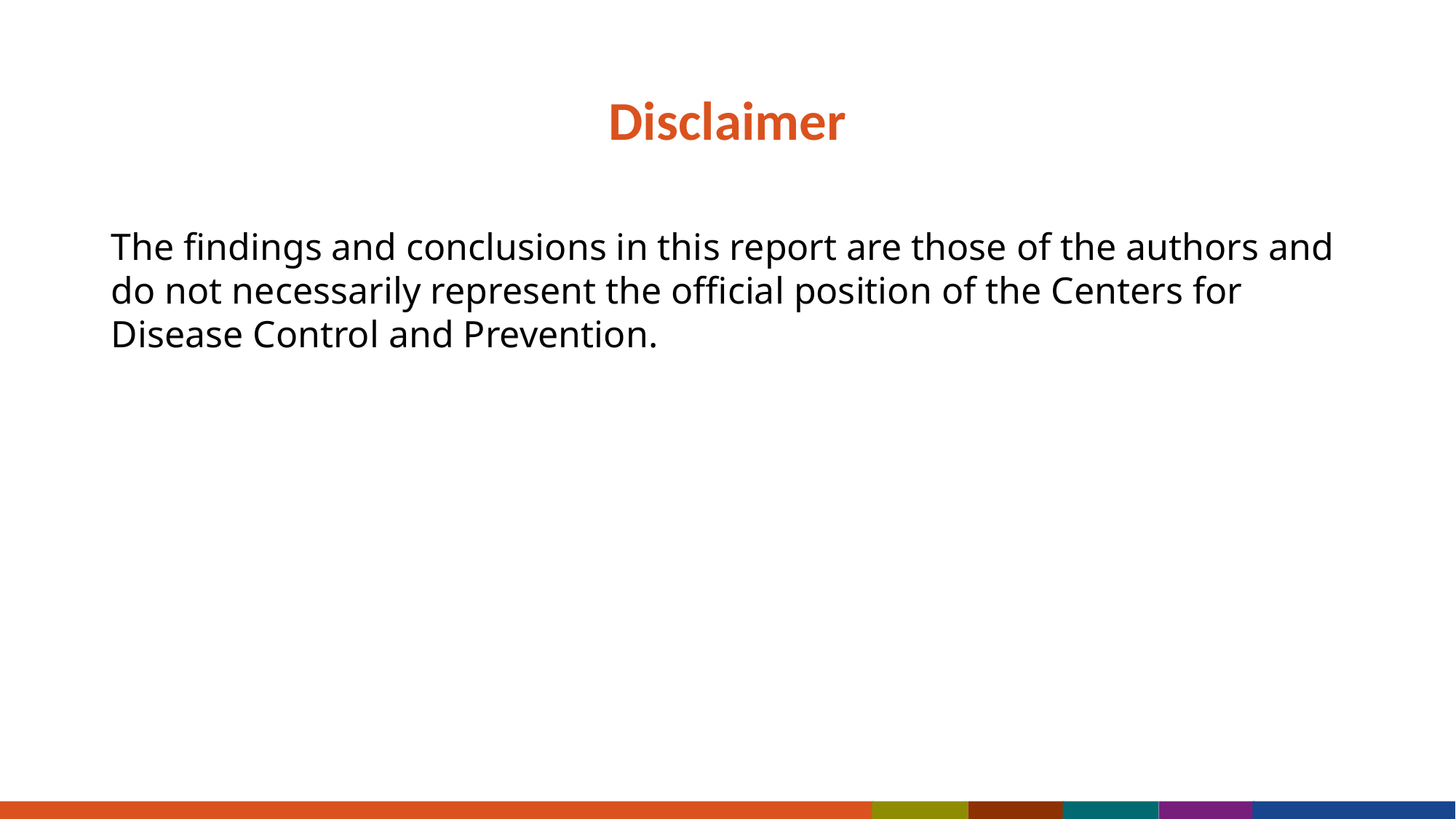

# Disclaimer
The findings and conclusions in this report are those of the authors and do not necessarily represent the official position of the Centers for Disease Control and Prevention.

## Slide 44
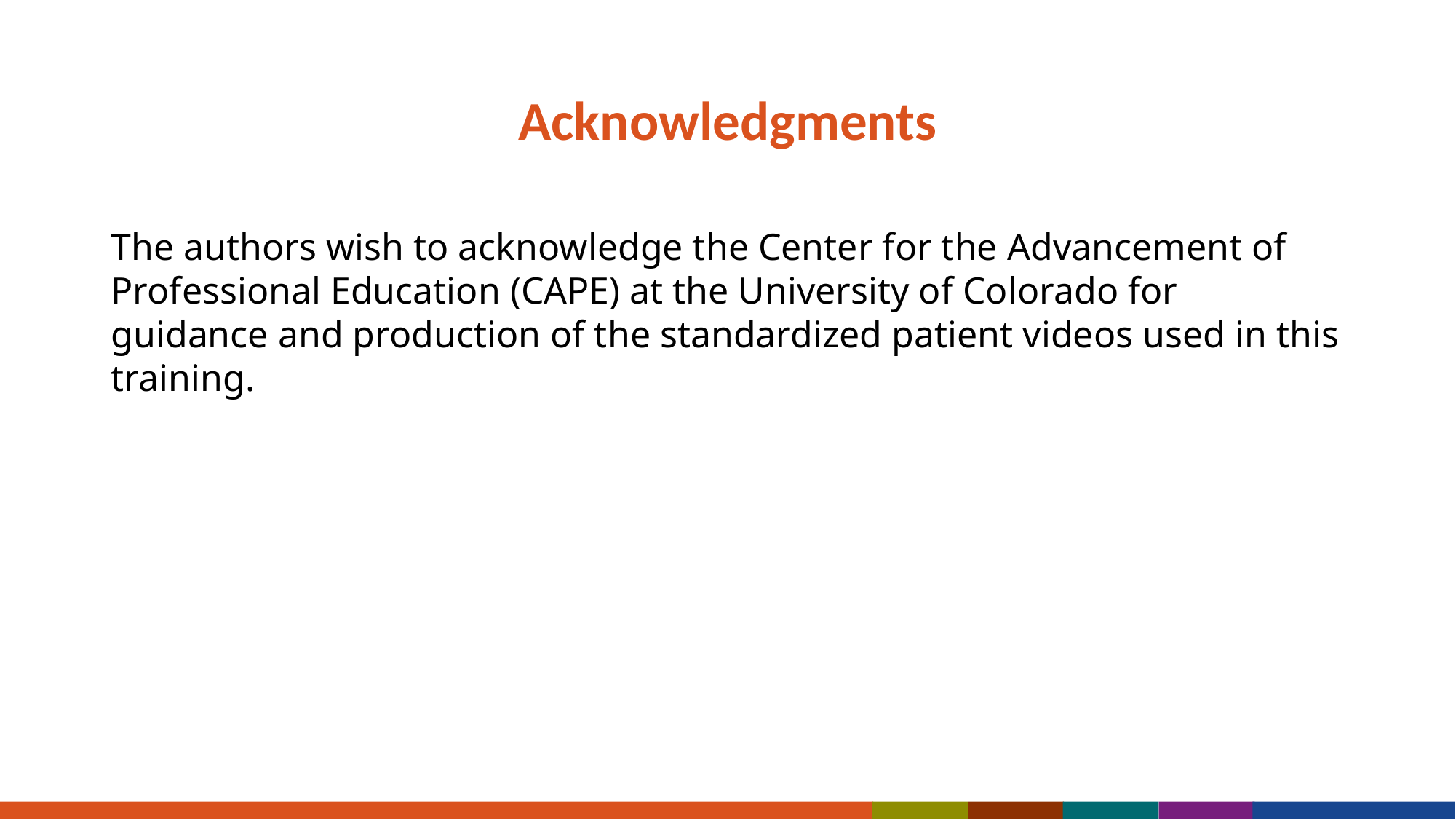

# Acknowledgments
The authors wish to acknowledge the Center for the Advancement of Professional Education (CAPE) at the University of Colorado for guidance and production of the standardized patient videos used in this training.

## Slide 45
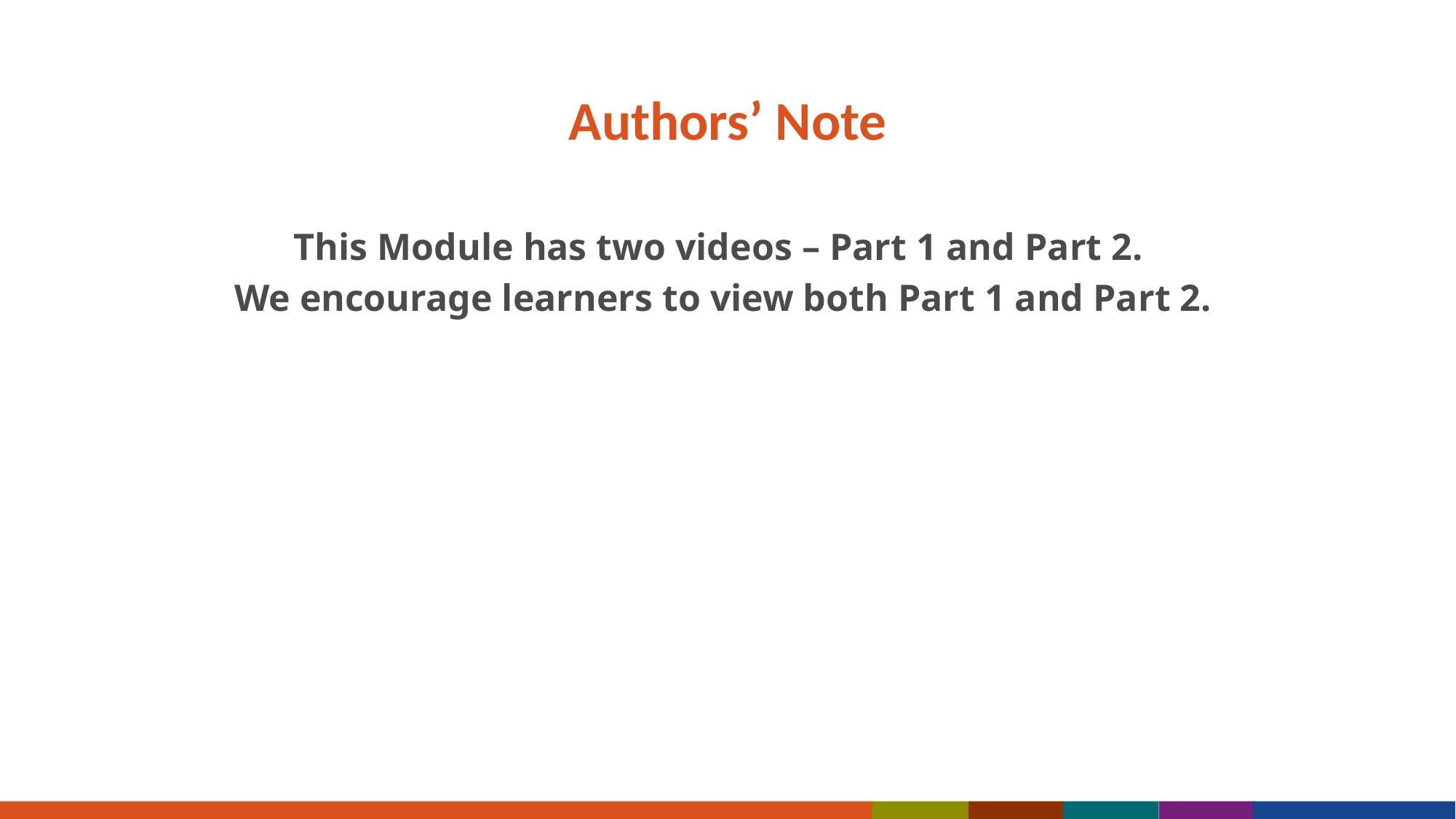

# Authors’ Note
This Module has two videos – Part 1 and Part 2.
We encourage learners to view both Part 1 and Part 2.

## Slide 46
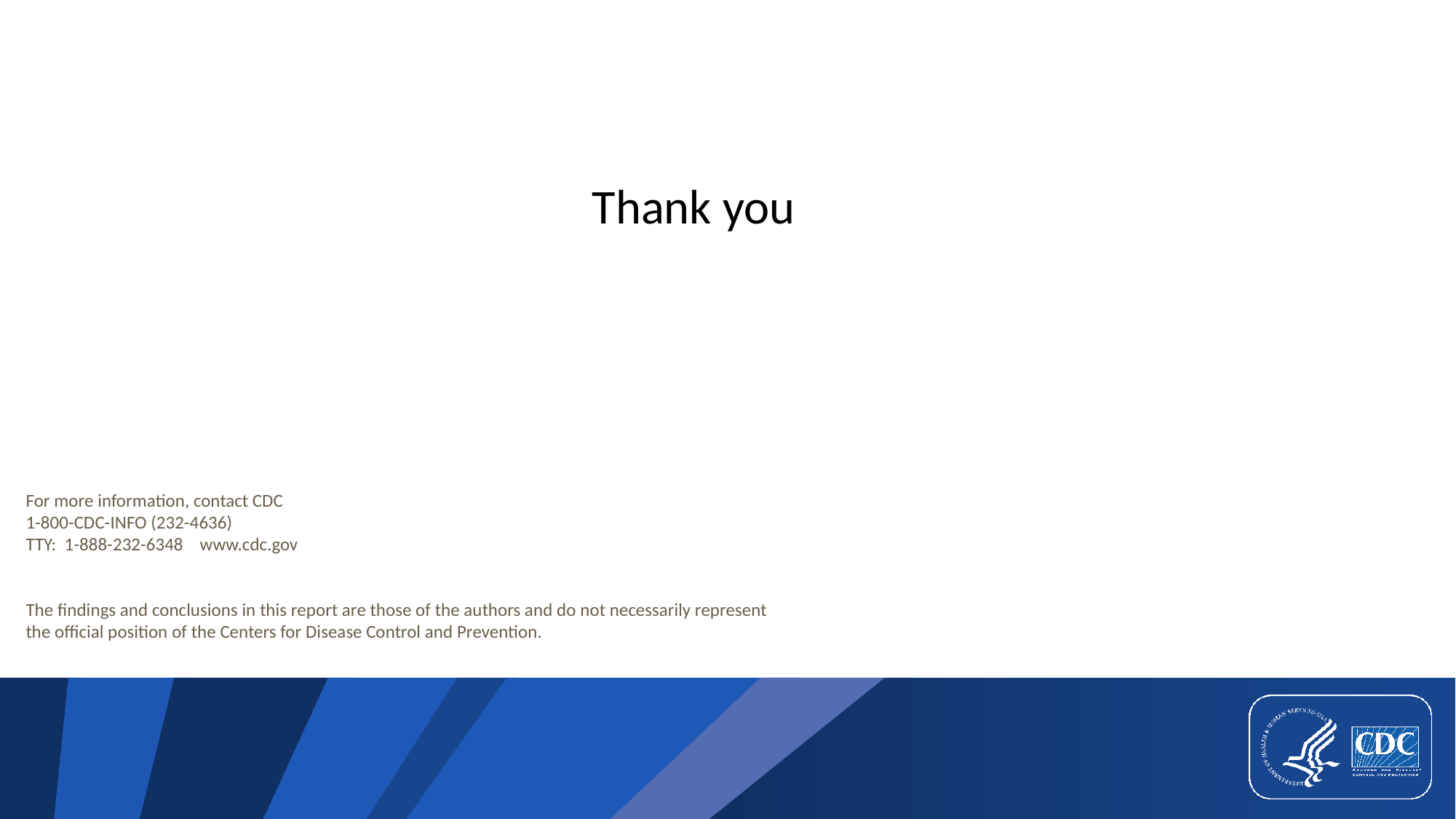

Thank you
