# Supplementary material for: Pediatric Myalgic Encephalomyelitis/Chronic Fatigue Syndrome (ME/CFS): A Diagnostic and Communication Case Study for Health Care Providers in Training
Source: MedEdPORTAL. 2025 Mar 14;21:11507. doi: 10.15766/mep_2374-8265.11507 (PMC11906784; doi:10.15766/mep_2374-8265.11507)
Supplement: Supplementary file 1 — MECFS Presentation.pptxMECFS Part 1.mp4MECFS Part 2.mp4Survey Questions.docx [file mep_2374-8265.11507-s001.zip › _Educational Summary Report_11507.pdf]

MedEdPORTAL® | The AAMC Journal of Teaching and Learning Resources

Original Publication

Open Access

Pediatric Myalgic Encephalomyelitis/Chronic Fatigue Syndrome (ME/CFS): A Diagnostic and Communication Case Study for Health Care Providers in Training

Dana J. Brimmer, PhD, MPH*, Jin-Mann S. Lin, PhD, Howard A. Selinger, MD, Anindita Issa, MD, Elizabeth A. Fall, MPH, Elizabeth R. Unger, PhD, MD

*Corresponding author: dyv4@cdc.gov

Abstract

Introduction: Myalgic encephalomyelitis/chronic fatigue syndrome (ME/CFS) is a chronic, complex illness. No diagnostic tests exist; illness evaluation relies on medical history, physical exam, and laboratory tests. While more is known about ME/CFS in adults, it can affect children and adolescents as a chronic condition. **Methods:** We implemented an ME/CFS pediatric educational activity (diagnosis, management, and communication) with medical, physician assistant, and nursing students at one university and with medical students at a second university. Pretests, two videos and slides, and posttests were completed in approximately 40 minutes. Evaluation included quantitative and qualitative measures for knowledge, attitudes, beliefs, confidence, and clinical information about ME/CFS. **Results:** The first group included 31 students who reported low familiarity and clinical exposure to ME/CFS. At posttest, 25 students (81%) recognized ME/CFS as a medical condition compared to seven (23%) at pretest. Using 0-5 scales, mean pretest-to-posttest ability to diagnose increased from 1.0 to 3.5, and confidence to communicate increased from 1.4 to 3.9. The second group, including 26 students pretest and 19 posttest, also reported low familiarity and clinical exposure The posttest showed increased self-rated ability to diagnose (pretest *M*: 0.6, posttest *M*: 3.3) and confidence to communicate (pretest *M*: 1.4, posttest *M*: 3.7). Qualitative feedback for this group showed understanding of pediatric ME/CFS symptoms, management, and communication. **Discussion:** This educational activity increased knowledge of ME/CFS as self-reported ability to make a diagnosis and increased confidence to communicate about pediatric ME/CFS. Participating students showed changes in attitudes towards ME/CFS as a medical condition.

Keywords
Myalgic Encephalomyelitis/Chronic Fatigue Syndrome, ME/CFS, Case-Based Learning, Communication Skills, Adolescent Medicine, Family Medicine, Pediatrics, Primary Care

Educational Objectives

By the end of this session, learners will be able to:

1. Recognize importance of a full medical and psychosocial evaluation for adolescents or children presenting with symptoms of myalgic encephalomyelitis/chronic fatigue syndrome (ME/CFS).
2. Identify specific symptoms of ME/CFS and possible abnormal physiologic and laboratory findings.
3. Describe the complexities of illness presentation in pediatric patients with ME/CFS.
4. Demonstrate confidence in communicating with pediatric patients with ME/CFS.

Citation:
Brimmer DJ, Lin JMS, Selinger HA, Issa A, Fall EA, Unger ER. Pediatric myalgic encephalomyelitis/chronic fatigue syndrome (ME/CFS): a diagnostic and communication case study for health care providers in training. *MedEdPORTAL.* 2025;21:11507.
https://doi.org/10.15766/mep_2374-8265.11507

Introduction

Myalgic encephalomyelitis/chronic fatigue syndrome (ME/CFS) is a debilitating, chronic, and complex illness that affects multiple body systems. People with ME/CFS experience significant limitations in their ability to participate in routine activities; these limitations last at least 6 months and are associated with profound fatigue, postexertional malaise (PEM), unrefreshing sleep, and at least one of the following: impaired memory or orthostatic intolerance.[1] No diagnostic tests exist to make a diagnosis; instead, clinicians must rely on evaluation, including

Copyright © 2025 Brimmer et al. This is an open-access publication distributed under the terms of the Creative Commons Attribution license.

medical history, physical exam, and laboratory tests, to assess for other conditions. Management focuses on treating the most disruptive and bothersome symptoms. ME/CFS affects people of all ages, races, ethnicities, and socioeconomic groups, and it is estimated that between 836,000 and 2.6 million Americans suffer from the illness.[1]

While more is known about ME/CFS in adults than in children, ME/CFS does affect adolescents and children, with current evidence suggesting it is more common in adolescents (12- to 18-year-olds) than younger school-age children. Estimates of pediatric ME/CFS prevalence vary in different studies from 0.10% to 0.75%.[2,3] The illness severely impacts a child's day-to-day life,[4] in part because pediatric patients with chronic illnesses like ME/CFS are at higher risk of chronic absenteeism from school and poor academic performance.[5] In cases of severe ME/CFS, students who are homebound or bedbound may not be able to attend school.[6]

In school settings, cognitive difficulties such as memory impairment, attention deficits, and concentration difficulties are commonly seen in children with ME/CFS.[2,7] As a result of ME/CFS, children often experience reduced participation in social, educational, and recreational activities, further diminishing their quality of life.[8] Chronic absenteeism is associated with poor educational outcomes and can have long-term implications for health and future income.[9,10] For example, asthma, psoriasis, and atopic dermatitis have all been associated with chronic absenteeism and poorer educational outcomes.[9,11-13] Moreover, when children miss school, often the parent or caregiver also misses work, leading to increased work absenteeism.[11] Family physicians play a vital role in identifying chronic conditions in children and can enhance their health outcomes by effectively communicating with schools.[9]

Despite the profound impact of ME/CFS on pediatric patients, medical student education is very limited in information regarding it.[14,15] Speight specifically highlighted the harms of mismanagement of pediatric patients with ME/CFS and concluded that medical training in this area is much needed.[16] After hearing from patient organizations and health care providers regarding the need for ME/CFS pediatric educational materials aimed at health care providers in training, the Centers for Disease Control and Prevention (CDC) developed pediatric ME/CFS educational information targeting practicing clinicians, parents/guardians, and educational professionals.[17] Nonetheless, there remains a need to enhance education directed at health care providers in training.

A case study published in *MedEdPORTAL* for ME/CFS assessment in adults showed positive outcomes in skills needed to make a diagnosis and better understanding of the illness.[18] Building on that prior work, this educational activity aims to educate medical, physician assistant, and nursing students about the key features of pediatric ME/CFS, including its clinical presentation, diagnosis, and management, while illustrating how the illness impacts young people's lives. Importantly, the case-based activity includes a parent's perspective and a component on how to communicate with school health providers.

Methods

We targeted medical, physician assistant, and nursing students in all years of training (medical school, 4 years; physician assistant program, 3 years; and nursing school, 2 years) to educate them about ME/CFS diagnostic criteria and how to recognize and manage ME/CFS in pediatric populations. The educational activity was implemented in two phases, with two different student groups. The first student group consisted of medical, physician assistant, and nursing students recruited through a health professional educational center at the University of Colorado. The second student group consisted of medical students at Quinnipiac University. Both universities' institutional review boards (IRBs) determined the educational evaluation fell under the category of exempt from review of human subject research as no personal identifying information was gathered (COMIRB protocol #15-0159 and Quinnipiac IRB protocol #09321). At the time of the activity, the term *chronic fatigue syndrome* was used for the two videos, but the name *myalgic encephalomyelitis/chronic fatigue syndrome* has since been adopted, and we refer to ME/CFS throughout.

Students were recruited during the spring semester of the school year through flyers and emails at each university. Coffee gift cards (University of Colorado) or $50 stipends (Quinnipiac University), funded through the educational center and medical school department, were offered upon full completion as an incentive for recruitment. The educational activity was designed to be flexible in terms of delivery format: either online or shown in a classroom group setting. We developed delivery of the activity to meet the needs of students who had limited time and might prefer to view the activity from a laptop. At the same time, we wanted teachers to be able to use the material in classroom settings. Students who agreed to participate in the educational activity received emails with links to the pre- and posttests, video, and slides.

Copyright © 2025 Brimmer et al. This is an open-access publication distributed under the terms of the Creative Commons Attribution license.

The educational activity followed a case study of an adolescent presenting with ME/CFS and included a PowerPoint presentation (Appendix A) and two accompanying videos (Appendices B and C). In one video (Appendix B), the adolescent made an outpatient visit with her mother, and in the other (Appendix C), the physician communicated with a school nurse regarding case management. Comparable to a previous learning activity published in *MedEdPORTAL,*[18] this one was developed by and was the product of the CDC.

The process of case study development has been previously described but included input from case studies provided by ME/CFS expert physicians, constructs from health behavior and education theory, and data from evidence-based literature, all of which guided development and review.[19] Briefly, the five-step process involved (1) developing case studies based on anonymous real-life patients, (2) using constructs from Bandura's social learning theory[20] and the theory of reasoned action[21] (which model behavior of physicians and use knowledge, attitudes, and beliefs to improve self-efficacy), (3) drafting scripts having both verbal and nonverbal components, (4) filming in studios to capture outpatient clinical rooms and a physician's office, and (5) finalizing the educational PowerPoint slides reinforcing the case study, diagnostic, and management guidelines.

For the assessment, all students were asked to complete a 21-item pretest before viewing the videos and slides, after which they completed a 19-item posttest (the posttest did not include two pretest questions asking about personal familiarity with ME/CFS and level of clinical exposure to ME/CFS as those questions captured a onetime measurement; Appendix D). The video and slides were made available once the pretest had been completed, and the posttest link was made available after the students had viewed the video and slides. Each video was 8 minutes in duration, while the slides took about 20 minutes to review. Since all students completed the activity online, they were instructed to do so at their own pace, in part to encourage completion of the educational activity.

Measures included ME/CFS knowledge, attitudes, and beliefs (e.g., personal familiarity with ME/CFS, clinical exposure to ME/CFS, communicating with patients with ME/CFS), as well as ME/CFS clinical diagnosis. In addition, we included open-ended questions to capture qualitative feedback. Measurements used 6-point Likert-type scales (0 = *not at all/never,* 5 = *highly*) and some multiple-choice questions. Qualitative responses were reviewed by four team members, and themes were discussed in the context of ME/CFS symptoms, complexities of the illness, and communication. Scale means were calculated for pre- and posttests.

Results

In 2018, 31 students participated in the first assessment, which included 15 medical students, seven physician assistant students, and nine nursing students. When asked about the year of study during which they learned about ME/CFS, 94% ($n = 29$) reported the first year, 74% ($n = 23$) the second year, and 19% ($n = 6$) the third year (note that students could respond for more than one year). No students reported learning about ME/CFS in their fourth year. Students had a mean personal familiarity with ME/CFS score of 1.4 (0 = *not at all familiar,* 5 = *highly familiar*) and a mean clinical exposure to ME/CFS score of 1.0 (0 = *never exposed,* 5 = *highly exposed*; Table 1).

On the pretest, 23% ($n = 7$) replied that ME/CFS is medical condition as compared to 81% ($n = 25$) on the posttest (Table 1). Conversely, 55% ($n = 17$) on the pretest thought ME/CFS was both a medical and psychiatric condition, while this changed to 16% ($n = 5$) on the posttest. When asked to rate their ability to diagnose a patient with ME/CFS on a 0-5 scale, the students' self-rating rose from a mean of 1.0 to 3.5 (Table 1). A similar increase was seen in the students' mean rating of their confidence to communicate with a patient with ME/CFS, rising from a mean of 1.4 to 3.9.

In 2022, 26 medical students participated in the second assessment and took the pretest. Of these, 19 completed the posttest. Of the total sample, 89% ($n = 23$) were in the 20-29 age range, and 77% ($n = 20$) were female. When medical students were asked on the pretest in which school year they learned about ME/CFS, 35% ($n = 9$) reported the first year, 42% ($n = 11$) the second year, and 27% ($n = 7$) the third year. Similar to the first group of students, no students reported learning about ME/CFS in their fourth year.

Students ($n = 26$) had a mean personal familiarity with ME/CFS score of 1.4 (0 = *not at all familiar,* 5 = *highly familiar*) and a mean clinical exposure to ME/CFS score of 0.5 (0 = *never exposed,* 5 = *highly exposed*; Table 1). On the pretest, 21% ($n = 4$) replied that ME/CFS is a medical condition as compared to 79% ($n = 15$) on the posttest (Table 1). Conversely, 58% ($n = 11$) on the pretest thought ME/CFS was both a medical and psychiatric condition, while this changed to 21% ($n = 4$) on the posttest. Students' self-rated ability on a 0-5 scale to diagnose (a measure of knowledge) a pediatric patient with ME/CFS rose from a mean of 0.6 to 3.3 ($n = 19$), and confidence in communicating

Copyright © 2025 Brimmer et al. This is an open-access publication distributed under the terms of the Creative Commons Attribution license.

Table 1. Pre- and Posttest Survey Questions

	Student Group One (*N* = 31)				Student Group Two (*N* = 19)			
	Pretest		Posttest		Pretest		Posttest	
Question	No. (%)	*M*	No. (%)	*M*	No. (%)	*M*	No. (%)	*M*
ME/CFS is:								
1. A medical condition	7 (23)		25 (81)		4 (21)		15 (79)	
2. Both medical and psychiatric condition	17 (55)		5 (16)		11 (58)		4 (21)	
3. A psychiatric condition or not sure	7 (23)		1 (3)		4 (21)		0 (0)	
Level of personal familiarity with ME/CFS[a]		1.4				1.4[b]		
Level of clinical exposure to ME/CFS[c]		1.0				0.5[b]		
Self-rated ability to diagnose ME/CFS[d]		1.0		3.5		0.6		3.3
Confidence to communicate with ME/CFS patient[e]		1.4		3.9		1.4		3.7

Abbreviation: ME/CFS, myalgic encephalomyelitis/chronic fatigue syndrome.
[a]Rated on a 6-point Likert-type scale (0 = *not at all familiar,* 5 = *highly familiar*).
[b]*N* = 26. Twenty-six medical students participated in Student Group Two; of these, 19 completed both the pre- and posttests.
[c]Rated on a 6-point Likert-type scale (0 = *never exposed,* 5 = *highly exposed*).
[d]Rated on a 6-point Likert-type scale (0 = *not capable,* 5 = *very capable*).
[e]Rated on a 6-point Likert-type scale (0 = *not confident,* 5 = *very confident*).

with a pediatric patient with ME/CFS rose from a mean of 1.4 to 3.7 ($n = 19$; Table 1).

In addition to knowledge, student attitudes towards ME/CFS changed after viewing the videos and slides. For example, qualitative responses indicated that after experiencing this activity, students were more aware of the definition of PEM. In the pretest, students generally had an incomplete understanding of PEM: “feeling tired or worn out after exerting oneself.” While this was not an inaccurate answer, in the posttest the descriptions were more detailed, such as “fatigue, pain, illness, or general discomfort that occurs after exercise, emotion, or other physical or mental exertion.” Qualitative responses also indicated that overall, students changed from a mindset of jumping to a differential diagnosis when a patient complained of fatigue to asking more probing questions about the fatigue and understanding the effect of fatigue on daily life (Table 2).

When asked in the posttest why they felt more confident in managing a pediatric patient with ME/CFS, medical students shared that they had “better understanding of the definition and shared symptoms,” were “more aware of specific diagnostic criteria,” “knew a bit more about the work up for ME/CFS,” and “understood the importance of communicating with school nurses/educators” (Table 2). When prompted for items to know about pediatric ME/CFS, they showed an understanding of the complexity of ME/CFS by mentioning the “variety of symptoms” and “how the condition can affect lifestyle” (Table 3). Some students pointed out how the video illustrated how to “be there for a patient even if all the answers are not yet known” and how to conduct “effective communication with another health professional and the school nurse who is part of the patient’s care team” (Table 3).

Discussion

Students who participated in the educational activity met the learning objectives and showed an increase in posttest scores demonstrating understanding of the ME/CFS diagnosis and confidence to communicate with pediatric patients. Before viewing the educational activity, students reported low personal familiarity and low levels of clinical exposure to ME/CFS, which supports the literature in terms of student exposure to ME/CFS. Before completing the educational activity, more students in both groups considered ME/CFS to be both a medical and psychiatric condition. After completion of the educational activity, results showed an increase in the number of students who considered ME/CFS a medical condition, as opposed to a combined medical and psychiatric condition. This change in belief that ME/CFS is a medical condition is important, as for far too long many patients with ME/CFS (including family members of pediatric patients) have commented that health care providers often do not take the illness seriously, with anecdotes of health care professionals commenting that it was “all in your head.”[14] An educational activity that impacts attitudes and beliefs is just as important as one that increases knowledge, as health care provider perspectives about patients impact the quality of care and management.

All students in both groups viewed the educational activity and took the pre- and posttests online. Although the activity was designed for potential use in classroom or group settings, its implementation online proved effective without any issues. Given students’ time constraints, this method may be preferred as it allows for flexibility in terms of completion. At the same time, facilitators could utilize the activity in the classroom as a participatory way to introduce pediatric ME/CFS. For example, instructors could adapt the pretest to measure where students

Copyright © 2025 Brimmer et al. This is an open-access publication distributed under the terms of the Creative Commons Attribution license.

Table 2. Qualitative Themes From Student Group Two: Confidence in Managing Pediatric ME/CFS

Theme	Quotations
Knowledge of ME/CFS	• "Understanding the diagnostic criteria, understanding treatments, understanding how to test."<br>• "I understand there are a variety of symptoms with some key symptoms."<br>• "I understand more of the symptoms associated with ME/CFS."<br>• "I know more of the signs and symptoms of ME/CFS."<br>• "More understanding of diagnostic requirements and knowing which symptoms to look for."<br>• "Recognition of common symptoms and common characteristics to each one."<br>• "Learned more about the symptoms and learned the criteria for diagnosis."<br>• "Knowing the symptoms and diagnostic criteria associated with ME/CFS."
Awareness towards ME/CFS	• "I am now more aware of the currently understood presentation of the condition, whereas before I did not have much of a clue about it. I feel comfortable knowing that, while the disease is still be studied, there is a way to manage symptoms, and affected children may be assisted also by requesting school accommodations. The videos allowed me to see an example of a doctor using all the tools he has to manage this patient."<br>• "The video of Dr. Brown handling the patient's questions despite uncertainty about CFS is a good example of being there for the patient even if all the answer are not yet known in medicine."<br>• "The module first taught me about ME/CFS, I was not aware of the diagnosis, but I now feel more confident that I will at least keep it on the differential. I am aware of things that can be done to treat some of the symptoms."<br>• "Watching the video was a good image to keep in mind when picturing my future patients."
Communication	• "Clear example of a patient presentation and interprofessional communication with a pediatric school nurse."<br>• "Awareness of diagnostic criteria and the thorough testing, treatment and communication required to help a patient experiencing a confusing disease."<br>• "I understand the importance of communicating with school nurses/educators."<br>• "I understand the condition more, I have references of good examples, and I am aware of important points of communications with that age group."<br>• "I understand how the condition can affect lifestyle."<br>• "I have seen which steps can be taken to improve their lifestyle."

Abbreviation: ME/CFS, myalgic encephalomyelitis/chronic fatigue syndrome.

Table 3. Qualitative Themes From Student Group Two: What to Know About Pediatric ME/CFS

Theme	Quotations
Diagnosis and symptoms	• "No definitive diagnostic test."<br>• "At least 3 months symptoms."<br>• "Symptoms include post-exertional malaise, joint and muscle pain, postural hypotension/tachycardia."<br>• "Symptoms can be different for different kids, there can be cognitive deficits, post-exertional malaise is the most common symptom."<br>• "Fatigue can be caused by mental or physical strain."<br>• "ME/CFS can be hard to diagnose and frustrating for patients and their families."<br>• "ME/CFS can be diagnosed in any patient and, to improve patient outcomes, should be diagnosed as early as possible."<br>• "Can happen after a physical illness (commonly infectious mononucleosis)."<br>• "It is primarily diagnosed by a period of unexplainable fatigue for at least 6 months that is exacerbated by exertion (physical or mental) that does not approve completely with rest *however chief complaint could be unrelated to fatigue (GI upset, confusion, etc.)."<br>• "Some common symptoms include lightheadedness, headache, difficulty with memory and concentration, sleep disturbance."<br>• "A large panel of testing may be required to rule-out similar diagnoses."<br>• "It is important to solicit all of the symptoms from the patient so that you can address and find exacerbating factors."<br>• "It involves post-exertional malaise, sleep problems or pain and its diagnosis is made by ruling out other medical problems."<br>• "Post-exertional malaise is a common feature, cognitive problems and trouble concentrating at school can be symptoms of ME/CFS."
Management	• "Can cause learning/school issues, can go away, can be managed in many ways."<br>• "Treatment should target specific symptoms."<br>• "Patients will likely need coordination between health care providers and the school in order to get accommodations."<br>• "Exercise regimens are not helpful to build tolerance."<br>• "Treatment options primarily focus on alleviating specific symptoms."<br>• "You must take a team approach to management."
Awareness and communication	• "Successful treatment requires collaboration with the child's school and other healthcare providers such as physical therapists."<br>• "The must communicate with the patient's family and school to ensure that the patient's support system."<br>• "ME/CFS is a debilitating and chronic illness."<br>• "It can have a severe impact on a patient's life."<br>• "It can be debilitating for patients which is why it is important to recognize the symptoms early."<br>• "That family and school faculty need to be included to approach the patient holistically."<br>• "It affects how they see themselves and their self confidence."

Abbreviations: GI, gastrointestinal; ME/CFS, myalgic encephalomyelitis/chronic fatigue syndrome.

Copyright © 2025 Brimmer et al. This is an open-access publication distributed under the terms of the Creative Commons Attribution license.

stand on ME/CFS pediatric familiarity. Next, showing the slides and videos could be accompanied by a group discussion. If the activity is to be part of an evaluation, the posttest, which is modifiable, could be completed. Facilitators are encouraged to modify demographic questions based on their needs and to use qualitative findings to assess not only knowledge and its application but also attitudes towards ME/CFS.

We used qualitative and quantitative measurements to triangulate data and strengthen findings in both depth and breadth. We also used qualitative process data to show that the best placement for this resource in the medical school curriculum may be second- and third-year medical student rotation as clinical knowledge in the class at these years corresponds to the content of the activity. For nursing and physician assistant students, placement in the second year of training is suggested.

In addition to ME/CFS, this education activity has the potential to affect knowledge, attitudes, and beliefs regarding other infection-associated chronic illnesses or conditions.[22,23] A meta-analysis of 21 studies of post-COVID conditions in children and adolescents reported a prevalence of 25.24%, with the most common symptoms being mood changes, fatigue, and sleep disturbances.[24] Long COVID has also been shown to impact school attendance, and this educational activity may help schools improve communication strategies. Learners taking this educational activity can extrapolate common management strategies for children with ME/CFS to those with other infection-associated illnesses, like long COVID.

The assessment of this activity had several limitations. First, the sample size for both student groups was small, and findings, therefore, may not be generalizable. However, outcomes from the posttest were similar among both groups in the West and East regions of the United States. Second, we did not have qualitative data and some demographics from the first student group. However, qualitative feedback for the second student group provided a range of responses. Students touched upon the themes in the educational activity, demonstrating their comprehension of the importance of attitudes towards patients. We had more medical students than physician assistant and nursing students participate, but feedback from all students was positive. Finally, self-reported qualitative measurements were used to assess understanding of the activity, which is common in educational and behavioral science learning. One challenge we faced was following up with students to complete the posttest. Despite the incentives and several email reminders, we had seven students (27%) in the second group who did not complete the posttest.

This pediatric ME/CFS educational activity is one tool that can help educate medical, physician assistant, and nursing students to recognize ME/CFS and communicate its management. Early identification of ME/CFS facilitates the development of management plans that can be coordinated with schools and families of affected patients. These plans could help lessen symptom burden and improve pediatric patients' ability to resume activities, such as attending school and socializing with friends. Viewing the educational module early in training years is a first step to broadening awareness of ME/CFS and potentially impacting future health care providers' knowledge, attitudes, and beliefs about this and related postinfectious syndromes.

Appendices

A. MECFS Presentation.pptx

B. MECFS Part 1.mp4

C. MECFS Part 2.mp4

D. Survey Questions.docx

All appendices are peer reviewed as integral parts of the Original Publication.

Dana J. Brimmer, PhD, MPH: Behavioral Visiting Scientist and Contractor, Chronic Viral Diseases Branch, Centers for Disease Control and Prevention; https://orcid.org/0000-0001-5975-9538

Jin-Mann S. Lin, PhD: Health Statistician and Team Leader, Chronic Viral Diseases Branch, Centers for Disease Control and Prevention; https://orcid.org/0000-0001-8286-6593

Howard A. Selinger, MD: Chair of Family Medicine, Frank H. Netter MD School of Medicine at Quinnipiac University

Anindita Issa, MD: Medical Officer, Chronic Viral Diseases Branch, Centers for Disease Control and Prevention

Elizabeth A. Fall, MPH: Associate Service Fellow, Chronic Viral Diseases Branch, Centers for Disease Control and Prevention

Elizabeth R. Unger, PhD, MD: Chief, Chronic Viral Diseases Branch, Centers for Disease Control and Prevention

Acknowledgments
The authors thank the Center for Advancing Professional Excellence at the University of Colorado, Quinnipiac University, and all students who participated in the educational activity assessment.

Copyright © 2025 Brimmer et al. This is an open-access publication distributed under the terms of the Creative Commons Attribution license.

Disclosures
None to report.

Funding/Support
Dr. Dana J. Brimmer received funding from the National Center for Emerging and Zoonotic Infectious Diseases.

Ethical Approval
The Quinnipiac University Institutional Review Board and the University of Colorado Institutional Review Board reviewed this project.

Disclaimer
The findings and conclusions in this publication are those of the authors and do not necessarily represent the official position of the Centers for Disease Control and Prevention.

References

1. Institute of Medicine. *Beyond Myalgic Encephalomyelitis/Chronic Fatigue Syndrome: Redefining an Illness*. National Academies Press; 2015. Accessed February 19, 2025. https://doi.org/10.17226/19012
2. Rowe PC, Underhill RA, Friedman KJ, et al. Myalgic encephalomyelitis/chronic fatigue syndrome diagnosis and management in young people: a primer. *Front Pediatr*. 2017;5:121. https://doi.org/10.3389/fped.2017.00121
3. Jason LA, Katz BZ, Sunnquist M, Torres C, Cotler J, Bhatia S. The prevalence of pediatric myalgic encephalomyelitis/chronic fatigue syndrome in a community-based sample. *Child Youth Care Forum*. 2020;49(4):563-579. https://doi.org/10.1007/s10566-019-09543-3
4. Ali S, Adamczyk L, Burgess M, Chalder T. Psychological and demographic factors associated with fatigue and social adjustment in young people with severe chronic fatigue syndrome/myalgic encephalomyelitis: a preliminary mixed-methods study. *J Behav Med*. 2019;42(5):898-910. https://doi.org/10.1007/s10865-019-00010-x
5. Crawley EM, Emond AM, Sterne JAC. Unidentified chronic fatigue syndrome/myalgic encephalomyelitis (CFS/ME) is a major cause of school absence: surveillance outcomes from school-based clinics. *BMJ Open*. 2011;1(2):e000252. https://doi.org/10.1136/bmjopen-2011-000252
6. Newton F. Improving academic success for students with myalgic encephalomyelitis/chronic fatigue. *Fatigue*. 2015;3(2):97-103. https://doi.org/10.1080/21641846.2015.1004831
7. Cornes O. Commentary: living with CFS/ME. *BMJ*. 2011;342: d3836. https://doi.org/10.1136/bmj.d3836
8. Parslow RM, Harris S, Broughton J, et al. Children's experiences of chronic fatigue syndrome/myalgic encephalomyelitis (CFS/ME): a systematic review and meta-ethnography of qualitative studies. *BMJ Open*. 2017;7(1):e012633. https://doi.org/10.1136/bmjopen-2016-012633
9. Allen CW, Diamond-Myrsten S, Rollins LK. School absenteeism in children and adolescents. *Am Fam Physician*. 2018;98(12): 738-744.
10. Balfanz R, Byrnes V. *Chronic Absenteeism: Summarizing What We Know From Nationally Available Data*. Johns Hopkins University Center for Social Organization of Schools; 2012.
11. Cheng BT, Silverberg JI. Association of pediatric atopic dermatitis and psoriasis with school absenteeism and parental work absenteeism: a cross-sectional United States population-based study. *J Am Acad Dermatol*. 2021;85(4):885-892. https://doi.org/10.1016/j.jaad.2021.02.069
12. Wang LY, Zhong Y, Wheeler L. Direct and indirect costs of asthma in school-age children. *Prev Chronic Dis*. 2005;2(1):A11.
13. Barnett SBL, Nurmagambetov TA. Costs of asthma in the United States: 2002-2007. *J Allergy Clin Immunol*. 2011;127(1): 145-152. https://doi.org/10.1016/j.jaci.2010.10.020
14. Peterson TM, Peterson TW, Emerson S, Regalbuto E, Evans MA, Jason LA. Coverage of CFS within U.S. medical schools. *Univers J Public Health*. 2013;1(4):177-179. https://doi.org/10.13189/ujph.2013.010404
15. Muirhead N, Muirhead J, Lavery G, Marsh B. Medical school education on myalgic encephalomyelitis. *Medicina (Kaunas)*. 2021;57(6):542. https://doi.org/10.3390/medicina57060542
16. Speight N. Severe ME in children. *Healthcare (Basel)*. 2020;8(3):211. https://doi.org/10.3390/healthcare8030211
17. ME/CFS in children. Centers for Disease Control and Prevention. May 13, 2024. Accessed February 19, 2025. https://www.cdc.gov/me-cfs/children/index.html
18. Brimmer DJ, Jones JF, Boneva R, Campbell C, Lin JMS, Unger ER. Assessment of ME/CFS (myalgic encephalomyelitis/chronic fatigue syndrome): a case study for health care providers. *MedEdPORTAL*. 2016;12:10527. https://doi.org/10.15766/mep_2374-8265.10527
19. Brimmer DJ, Campbell C, Bonner K, Lin JM. News from the CDC: chronic fatigue syndrome (CFS) and standardized patient videos—a novel approach to educating medical students about CFS. *Transl Behav Med*. 2013;3(4):338-339. https://doi.org/10.1007/s13142-013-0229-9
20. Bandura A. *Social Learning Theory*. General Learning Press; 1971.
21. Glanz K, Lewis FM, Rimer BK, eds. *Health Behavior and Health Education: Theory, Research, and Practice*. 2nd ed. Jossey-Bass; 1997.
22. Buonsenso D, Di Gennaro L, De Rose C, et al. Long-term outcomes of pediatric infections: from traditional infectious diseases to long Covid. *Future Microbiol*. 2022;17(7):551-571. https://doi.org/10.2217/fmb-2022-0031
23. National Academies of Sciences, Engineering, and Medicine. *Toward a Common Research Agenda in Infection-Associated*

Copyright © 2025 Brimmer et al. This is an open-access publication distributed under the terms of the Creative Commons Attribution license.

Chronic Illnesses: Proceedings of a Workshop. National Academies Press; 2024. Accessed February 19, 2025. https://doi.org/10.17226/27462

24. Lopez-Leon S, Wegman-Ostrosky T, Ayuzo del Valle NC, et al. Long-COVID in children and adolescents: a systematic review and meta-analyses. *Sci Rep*. 2022;12:9950. https://doi.org/10.1038/s41598-022-13495-5

Received: July 24, 2024
Accepted: January 27, 2025
Published: March 14, 2025

Copyright © 2025 Brimmer et al. This is an open-access publication distributed under the terms of the Creative Commons Attribution license.
